# MCM7 promotes liver fibrosis by transcriptionally regulating IL11 via the SHCBP1-RACGAP1-STAT3 axis

**DOI:** 10.1038/s41419-025-07937-x

**Published:** 2025-08-11

**Authors:** Cheng Wang, Mengling Ye, Tian Xu, Jiayi Feng, Jiaxi Zhang, Wenjuan Yang, Qian Fang, Guangbo Mei, Xuejun Zhao, Kejun Liu, Huiqin Zhou, Yaru Yu, Yujun Peng, Na Kuang, Xuebing Qiu, Qinping Zhong, Hongying Zong, Huifen Dong, Zhenping Ming, Yan Xiong, Rui Zhou

**Affiliations:** 1 Hubei Province Key Laboratory of Allergy and Immunology, School of Basic Medical Sciences, Wuhan University, Wuhan, Hubei China; 2 Department of Medical Parasitology, School of Basic Medical Sciences, Wuhan University, Wuhan, Hubei China; 3 Department of Research, Guangxi Medical University Cancer Hospital, Nanning, Guangxi China; 4 Department of Clinical Laboratory, Fuwai Yunnan Cardiovascular Hospital, Kunming, Yunnan China; 5 Department of Clinical Laboratory, Guizhou Provincial People’s Hospital, Guiyang, Guizhou China; 6 Ecological Station of Oncomelania hupensis in Gong’an County, Jingzhou, Hubei China; 7 Kindstar Diagnostics, Wuhan, Hubei China; 8 Teaching Center for Basic Medical Experiments, School of Basic Medical Sciences, Wuhan University, Wuhan, Hubei China; 9 Zhongnan Hospital of Wuhan University, Institute of Hepatobiliary Diseases of Wuhan University, Transplant Center of Wuhan University, National Quality Control Center for Donated Organ Procurement, Hubei Key Laboratory of Medical Technology on Transplantation, Hubei Provincial Clinical Research Center for Natural Polymer Biological Liver, Wuhan, Hubei China

**Keywords:** Interleukins, Parasitic infection

## Abstract

Liver fibrosis is driven by the persistent activation of hepatic stellate cells (HSCs) through inflammatory factors released from various cell types, including stressed hepatocytes, yet the underlying mechanisms are not fully understood. Here, we show that minichromosome maintenance complex component 7 (MCM7) is predominantly upregulated in hepatocytes of liver fibrosis mouse models and in liver cirrhosis patients. Hepatocyte-specific overexpression of MCM7 accelerates fibrosis progression, while its knockdown mitigates it in *Schistosoma japonicum*- and CCl4-induced fibrosis models. Mechanistically, MCM7 interacts with SHCBP1, promoting *IL11* transcription via the SHCBP1-RACGAP1-STAT3 axis. Moreover, neutralizing IL11 significantly attenuated the enhanced activation of HSCs induced by MCM7 overexpression in vitro. Additionally, recombinant human IL11 (rhIL11), which effectively inhibits endogenous IL11 signaling, significantly attenuated the exacerbation of liver fibrosis driven by MCM7 overexpression in vivo. These findings identify MCM7 in hepatocytes as a key regulator of HSC activation through IL11 and highlight its potential as a therapeutic target for liver fibrosis treatment.

**Figure:**
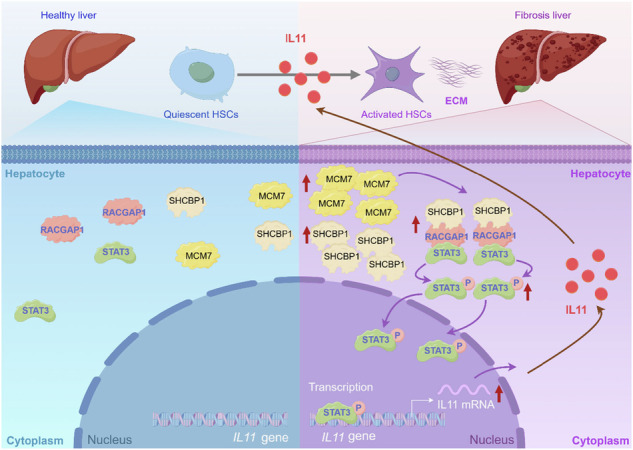
Liver fibrosis conditions induce upregulation of MCM7 and SHCBP1 in hepatocytes. Elevated MCM7 promotes the interaction between SHCBP1 and RACGAP1, which in turn facilitates the binding of RACGAP1 to STAT3 and induces its phosphorylation. Phosphorylated STAT3 translocates to the nucleus, activating transcription of the *IL11* gene. Secreted IL11 acts in a paracrine manner to enhance hepatic stellate cell activation, further exacerbating liver fibrosis.

Liver fibrosis conditions induce upregulation of MCM7 and SHCBP1 in hepatocytes. Elevated MCM7 promotes the interaction between SHCBP1 and RACGAP1, which in turn facilitates the binding of RACGAP1 to STAT3 and induces its phosphorylation. Phosphorylated STAT3 translocates to the nucleus, activating transcription of the *IL11* gene. Secreted IL11 acts in a paracrine manner to enhance hepatic stellate cell activation, further exacerbating liver fibrosis.

## Introduction

Liver fibrosis, a chronic liver injury arising from diverse factors such as alcoholic liver disease, viral hepatitis, drug-induced liver injury, autoimmune diseases, and schistosomiasis [1, 2], affects an estimated 844 million individuals globally and is associated with an annual mortality rate of 2 million [3]. Without effective intervention, this condition can progress to liver cirrhosis or even liver cancer [4]. During the progression of liver fibrosis, hepatocytes play a pivotal role by altering their gene expression and secretion profiles in response to injury [5]. Stressed or injured hepatocytes can trigger the release of signaling molecules, thereby igniting the hepatic inflammatory response and activating hepatic stellate cells (HSCs), which further promotes fibrogenesis [6]. Although there is a preliminary understanding of the specific roles and mechanisms of hepatocytes in liver fibrosis, the details and full picture of these mechanisms still need further clarification.

The minichromosome maintenance (MCM) complex, crucial for DNA replication in eukaryotes and archaea, consists of MCM2–MCM7 [7]. Studies have found that, in mammalian cells, the amount of MCM protein far exceeds what is actually needed at potential replication initiation sites (approximately 10–100 times). Moreover, most MCM proteins have been shown not to co-localize with DNA synthesis sites [8, 9]. This phenomenon, known as the “MCM paradox,” has sparked widespread speculation about the possible additional biological functions of MCM proteins [10]. Minichromosome Maintenance Protein 7 (MCM7) is a key component of the pre-replication complex, primarily functioning at the initiation sites of DNA replication, regulating the formation of replication forks and the activity of DNA helicases [11]. Over the past two decades, most MCM7-based studies have focused on its function in cancer, consistently identified as upregulated in various malignancies [12–17]. Previous research has shown that MCM7 promotes hepatocellular carcinoma (HCC) progression via the MCM7-cyclin D1 signaling pathway, suggesting its potential as a target for therapeutic intervention in hepatocellular carcinoma [18]. Notably, MCM7 has been found to bind to the SF3B3 subunit of the RNA splicing complex, thereby regulating the expression of fibrosis-related factor receptors EGFR and PDGFR at the splicing level in prostate cancer [19]. Previous studies have revealed that EGFR signaling activation induces hepatocyte epithelial-mesenchymal transition and PDGFR activation is essential for hepatic stellate cell activation, contributing to the development of liver fibrosis [20, 21]. However, the specific role of MCM7 in liver fibrosis remains unknown.

In this study, we observed significant upregulation of MCM7 expression in hepatocytes in liver fibrosis models induced by *Schistosoma japonicum* and carbon tetrachloride (CCl4), as well as in patients with liver cirrhosis. Hepatocyte-specific MCM7 deficiency protected against liver fibrosis in a *S. japonicum*-and CCl4-induced mouse model. Overexpression of MCM7 in the liver exacerbated liver fibrosis. Mechanistically, our findings demonstrated that MCM7 activates IL11 expression via the SHCBP1-RACGAP1-STAT3 signaling pathway, thereby promoting the hepatic stellate cell activation and driving liver fibrosis progression. This interaction between MCM7 and SHCBP1 regulates IL11 expression through the RACGAP1-STAT3 pathway, underscoring a novel mechanism for liver fibrosis progression. Our data indicate that targeting MCM7 or its associated signaling pathway may represent a promising therapeutic strategy for the treatment of liver fibrosis.

## Materials and methods

### Human patient samples

Human liver tissue and serum samples from healthy individuals and patients with liver cirrhosis were provided by the Zhongnan Hospital of Wuhan University. The tissue samples were immediately snap-frozen in liquid nitrogen upon collection to preserve their integrity. Blood samples were centrifuged to separate the serum, which was then aliquoted and stored at −80 °C for future analysis. All subjects provided written informed consent. The study, which included both liver tissue and serum samples, complied with the Declaration of *Helsinki and Declaration of Istanbul* and was approved by the Ethics Committee of Wuhan University (NO.WHU-LFMD-IRB2024036).

### Establishment of the hepatic fibrosis model using *S. japonicum* or CCl4

Male BALB/c mice purchased from the Hubei Provincial Center for Disease Control and Prevention (Wuhan, China), 7–8 weeks old, weighing 22–25 g, were used. *Oncomelania hupensis* snails were provided by the Snail Ecological Station of Gongan County (Jingzhou, China). Liver fibrosis models were established in BALB/c mice through either percutaneous infection with 16 *S. japonicum* cercariae or intraperitoneal (i.p.) injection of CCl4 (Sigma-Aldrich; 10 ml/kg, diluted 1:4 in olive oil, filtered, twice weekly for 6 weeks). Recombinant human IL11 (rhIL11; UniProtKB: P20809, Genscript) was administered i.p. to mice at a dose of 100 mg/kg, once every two days. Mice were randomly assigned to groups (*n* = 6 per group) and included if healthy, with exclusion for severe distress or mortality during treatment. The investigators were blinded to the group allocation during the experiment. All animal experiments were approved by the Institutional Animal Care and Use Committee of Wuhan University (IACUC-AF048).

### Construction and stereotactic injection of vectors for MCM7-knockdown or overexpression

Recombinant adeno-associated virus (AAV) vectors were obtained from Dr. Y.C. Xia (Wuhan University). An AAV-CMV vector (Addgene; Cat No.105530) was constructed to express the complete coding sequence of *Mcm7* (AAV-MCM7 for Mcm7 overexpression), while empty vectors were used as negative controls. The recombinant plasmids were produced using a triple-transfection helper-free method and purified according to a previously published protocol [22]. Specifically, HEK293T cells (ATCC) were transfected with the AAV-CMV, AAV8 (Addgene; Cat No.112864), and AAV-helper (Addgene; Cat No.81070) plasmids in 150-mm dishes at 80% confluence. For Mcm7 knockdown, AAV-shCtrl (Addgene; Cat No.60958) vectors expressing a short hairpin RNA targeting *Mcm7* mRNA (AAV-shMcm7) were constructed and verified by sequencing. AAV-shCtrl vectors served as negative controls. HEK293T cells were transfected with the AAV-shCtrl, AAV-DJ (Addgene; Cat No.130878), and AAV-helper (Addgene; Cat No.81070) plasmids. GFP was used as a reporter. Similarly, AAV-shShcbp1 and AAV-SHCBP1 vectors were constructed for *Shcbp1* mRNA knockdown and overexpression, respectively, using the same methods described above for the *Mcm7* experiments. Viral titers were determined by quantitative PCR. The final viral preparations were resuspended in sterile saline solution and had a titer of 1 × 10^12^ viral particles/ml.

### Isolation of primary hepatocytes and HSCs

Primary hepatocytes and HSCs were isolated from the indicated mice through the portal vein after anesthesia, as previously described [23]. Briefly, the liver was perfused in situ with Ca^2^^+^-free Hank’s Balanced Salt solution for 15 min, followed by sequential perfusion with 100 mL of 0.2% pronase solution and 0.2% collagenase type IV (BioFroxx, Einhausen, Germany) until the liver appeared digested and pale in color. The resulting cell suspension was filtered through a 100 μm pore size nylon mesh, and then centrifuged at 50 × *g*. The pellet was collected for primary hepatocyte isolation, while the superannuate was further processed for primary HSC isolation using density gradient centrifugation. The isolated primary hepatocytes and HSCs were cultured in DMEM medium supplemented with 10% heat-inactivated fetal bovine serum (FBS).

### Cell culture and treatment

The human hepatocyte cell line HepG2 and the human embryonic kidney cell line HEK293T were obtained from the American Type Culture Collection (ATCC). The human hepatic stellate cell line LX-2 was provided by Dr. Yong Wang (Nanjing Medical University), with its original source being ATCC. Additionally, the mouse hepatocyte cell line AML12 was utilized in this study. All the cell lines were authenticated by STR profiling and tested clean for mycoplasma contamination.

Cells were cultured in DMEM with 10% FBS. Recombinant human IL-1β (PeproTech, Rocky Hill, NJ, USA) and recombinant mouse IL-6 (PeproTech, Rocky Hill, NJ, USA) at a dose of 10 ng/ml were added to stimulate cells. Additionally, 10 μM XMU-MP-1 (MedChemExpress, Monmouth Junction, NJ, USA) was used to inhibit the Hippo signaling pathway, and 10 μM C188-9 (MedChemExpress, Monmouth Junction, NJ, USA) was used to inhibit the STAT3 signaling pathway. LX-2 cells and primary HSCs were treated with 10 μg/ml IL11 neutralizing antibody or IgG control (R&D Systems, Minneapolis, MN, USA) for 24 h.

### Co-culture of HSCs with hepatocytes

Primary HSCs were isolated from normal mice and cultured overnight, while primary hepatocytes from liver fibrosis mice, with or without MCM7 overexpression, were isolated and cultured for 24 h. The conditioned medium from these primary hepatocyte cultures was collected, filtered through a 0.2-μm strainer, and then added to the primary HSC cultures. Additionally, HepG2 cells were transfected with an MCM7 overexpression vector or a control vector, then stimulated with IL-1β. The conditioned medium from these HepG2 cell cultures was co-cultured with the human HSC cell line LX-2 for 24 h. Lysates from the primary HSCs and LX-2 cells were then harvested for further experiments.

### Quantitative reverse transcriptase PCR (qRT-PCR)

Total RNA was isolated from the cells using TRIzol reagent (Life, USA), following the manufacturer’s instructions. cDNA synthesis was performed using the HiScript II Q RT Super Mix for qPCR (Vazyme Biotech, Nanjing, China). The expression levels of target genes were measured by quantitative real-time PCR (qRT-PCR) using SYBR Green (Vazyme Biotech, Nanjing, China) on the Applied Biosystems 7300 Fast Real-time PCR System (USA). The relative RNA expression levels were calculated using the 2^−ΔΔCt^ method, with GAPDH as the internal control. The primer sequences are listed in Supplemental Table 1.

### RNA-Seq and data analysis

Total RNA was extracted from the samples using the RNeasy Kit (Qiagen, Hilden, Germany), and the RNA quality was assessed using an Agilent Bioanalyzer 2100. The mRNA was then purified using the KAPA mRNA Capture Kits (Roche, Basel, Switzerland), and cDNA libraries were prepared using the KAPA RNA HyperPrep Kits (Roche, Basel, Switzerland) at Kindstar Global (Wuhan, China). Equal amounts of cDNA library from each sample were pooled and sequenced on an Illumina HiSeq × platform, with 150-bp paired-end sequencing. The sequencing depth ranged from 85.567278 to 130.156120 million reads per sample, with a median of 113.913107 million reads. The sequencing reads were mapped to the GRCh38/GRCm39 genome assemblies using Hisat2 v2.1.0 with the default settings. The aligned reads were then converted to bigwig coverage files using reads per million. Genome annotations were extracted from the Ensembl GRCh38/GRCm39 Ens_96 database and used to count the reads with htseq-count v0.13.5. Significantly differentially expressed genes (DEGs) were identified using R (version 3.6.0) and the DESeq2 package, with criteria set at an adjusted *p*-value (padj) <0.05 and an absolute log2 fold change (log2FC) ≥1. Functional enrichment analysis of the identified DEGs was performed using the Gene Ontology and Kyoto Encyclopedia of Genes and Genomes databases.

### Immunoprecipitation mass spectrometry assay

Stable HL7702 hepatocyte cell lines expressing MCM7 (pHAGE-MCM7) were cultured for 48 h. The cells were then dissolved in a cell suspension containing: 10 mM HEPES (pH 7.9), 10 mM KCl, 1.5 mM MgCl_2_, 340 mM sucrose (~12% w/v), 10% (v/v) glycerol, 0.5 mM DTT, protease inhibitor cocktail (1:100), and 10 mM sodium butyrate (HDAC inhibitor from a 1 M stock solution). The samples were treated with an equal volume of 0.2% Triton, resulting in a final Triton concentration of 0.1%, and a final CaCl_2_ concentration of 1 mM. Anti-MCM7 antibodies were added to the samples, which were then incubated overnight at 4 °C. Subsequently, A/G-agarose beads were added and incubated for 4 h. The beads were washed twice with PBST, and 100 μl of elution buffer mixed with SDS loading buffer was added to each sample. Subsequently, 15 μl of supernatant was collected for gel running, and silver staining was used for detection, whereas the remaining samples were stained with Coomassie blue. The whole gel was cut into pieces, washed three times with H_2_O, and destained using a solution containing 35% ACN and 50 mM NH_4_HCO_3_. After dehydration, reduction and alkylation of cysteines, the gel was digested in 50 mM NH_4_HCO_3_ solution with modified trypsin at 37 °C overnight. The resulting tryptic peptides were analyzed on a Q Exactive HF-X mass spectrometer coupled with an Easy-nLC 1200 system (Thermo Scientific, USA) with a 60 min gradient. The mass spectrometer was operated in data-dependent acquisition mode with full scans (*R* = 60 K, AGC = 3e6, max IT = 20 ms, scan range = 300–1800 m/z) followed by 25 MS/MS scans (*R* = 15 K, AGC = 2e5, max IT = 50 ms). All MS/MS spectra were analyzed by pFind software (version 3.0.11) 2 against the human protein database combined with the reverse decoy database and common contaminants. Trypsin digestion allowed up to two missed cleavages, and an open-search algorithm implemented in pFind was employed for data analysis. The precursor and fragment ion mass tolerances were 20 ppm and 20 ppm, respectively. Minimum peptide length was set at 6, while the estimated false discovery rate threshold for peptide and protein was specified at maximum 1%. The mass spectrometry analysis for SHCBP1 was performed using the same protocol. The antibodies used in this study are listed in Supplemental Table 3.

### Western blot

Total proteins were extracted from the tissues or cells using pre-chilled RIPA buffer (Beyotime, Shanghai, China) supplemented with protease inhibitors (Thermo Scientific, USA). The protein concentration was determined using a Bicinchoninic Acid Protein Assay Kit (Thermo Scientific, USA). Equal amounts of the protein samples were separated by 10% SDS-PAGE and then transferred to 0.45 μm PVDF membranes (Millipore, USA). The membranes were blocked with 5% non-fat milk in TBST for 1 h, and then incubated with the corresponding primary antibodies overnight at 4 °C. After three washes with TBST, the membranes were incubated with HRP-conjugated secondary antibodies for 1 h at room temperature. Following three additional TBST washes, the membranes were incubated with ECL Western blot Substrate (Bio-Rad, Hercules, CA, USA) and exposed to X-Ray Super RX Films (Fujifilm, Tokyo, Japan) for detection. The antibodies used in this study are listed in Supplemental Table 3.

### Co-Immunoprecipitation (Co-IP)

Cells were lysed in cold IP buffer (150 mM NaCl, 25 mM Tris-HCl, pH 8.0, 1 mM EDTA, 1% Triton X-100, 0.5% Nonidet P-40) containing a protease inhibitor cocktail. The cell lysates were then centrifuged at 12,000 × *g* for 30 min. The obtained cell lysates were first pre-cleared with protein A/G-agarose beads (Smart-Lifesciences, Changzhou, China) at 4 °C for 6 h. Subsequently, the pre-cleared lysates were incubated with an anti-MCM7 antibody or an isotype-matched control IgG overnight at 4 °C. MCM7 and its associated proteins were then precipitated by incubating the lysates with pre-equilibrated protein A/G-agarose beads at 4 °C for 6 h. The immunoprecipitates were thoroughly washed with cold PBST, heated in 1× loading buffer at 95 °C for 5 min, and then analyzed by immunoblotting. The precipitates were then washed and analyzed by immunoblotting. The antibodies used in this study are listed in Supplemental Table 3.

### GST/His pull-down assay

Expression of recombinant GST-tagged truncated MCM7 fragments and His-tagged serial truncations of SHCBP1 proteins were induced in BL21 cells at 28 °C for 24 h by 0.1 mM isopropyl β-D-1-thiogalactopyranoside. Bacterial cells were lysed using a lysis buffer containing 50 mM Tris-HCl (pH 7.4), 150 mM NaCl, and 0.5% Triton X-100. Recombinant proteins were then purified using Glutathione Sepharose 4B beads (Smart-Lifesciences, Changzhou, China) and HisPur™ Ni-NTA Resin (ThermoFisher, Waltham, MA, USA). Protein concentrations were determined by measuring their optical density absorbance at 280 nm. The 100 µl GST-tagged truncated MCM7 fragments were mixed with 100 µl His-tagged full-length SHCBP1 as well as His-tagged serial truncations of SHCBP1 with GST-tagged MCM7 in 0.5 ml binding buffer (50 mM Tris-HCl, pH 7.4, 150 mM NaCl, and 0.5% Triton X-100). The binding reaction was performed with Glutathione Sepharose 4B beads and HisPur™ Ni-NTA Resin overnight at 4 °C, and the beads were subsequently washed three times with the binding buffer. Proteins were analyzed by Western blot using GST and His antibodies. The antibodies used in this study are listed in Supplemental Table 3.

### Chromatin Immunoprecipitation (ChIP)

ChIP was performed as previously described [24]. Briefly, the cells were cross-linked with 1% paraformaldehyde for 10 min, and the reaction was quenched with glycine for 5 min. After cell lysis, the fixed chromatin was fragmented to 100–500 base pair DNA fragments using Diagenode Bioruptor Plus non-contact sonication device (Diagenode, Liège, Belgium). The DNA fragments were then immunoprecipitated using a STAT3 antibody and Protein A/G beads. The precipitated DNA samples were subsequently analyzed by quantitative PCR (qPCR). The antibodies used in this study are listed in Supplemental Table 3.

### Luciferase reporter assay

HepG2 cells were seeded in a 24-well plate at a density of 1 × 10^5^ cells per well, 24 h prior to transfection. The pGL3-basic reporter vectors containing the *IL11* promoter fragment were then transfected using 1 μL of Neofect™ DNA Transfection Reagent (Neofect, Beijing, China), following the manufacturer’s instructions. After 24 h of transfection, the cells were harvested and lysed using reporter lysis buffer (Promega, Madison, WI, USA). Luciferase activities were measured using a Luciferase Assay System (Promega, Madison, WI, USA), and the firefly luciferase activities were normalized to the Renilla luciferase activities.

### Histology and immunohistochemistry (IHC)

The liver samples were fixed with 4% paraformaldehyde and embedded in paraffin. The sectioned tissues were stained with Mayer’s hematoxylin and eosin (H&E) to measure the size of hepatic granulomas using a calibrated measuring eyepiece. Additionally, the tissues were stained with Masson’s trichrome to determine the extent of fibrosis. For the immunohistochemistry analysis, the liver sections (4 μm) were deparaffinized in xylene and hydrated using a graded ethanol-deionized water series. Antigen retrieval was performed using an Ethylene Diamine Tetraacetic Acid (EDTA) repair solution via microwave treatment. The sections were subsequently incubated with 3% hydrogen peroxide (H_2_O_2_) for 10 min at room temperature, followed by incubation with primary antibodies against MCM7, SHCBP1, COL1A1, IL11, and α-SMA. Next, the sections were incubated with a horseradish peroxidase-labeled secondary antibody at room temperature for 1 h. The immunoreactive signals were developed using a DAB reagent solution, and the nuclei were counterstained with hematoxylin solution. The stained sections were scanned and visualized using an Aperio Versa 8 system (Leica Biosystems, Wetzlar, Germany). For each group, whole-slide imaging was conducted on distinct tissue sections, with random fields of view selected for analysis. The positive areas were calculated and quantified using ImageJ software. The antibodies used in this study are listed in Supplemental Table 3.

### Immunofluorescence histochemical analysis

Liver tissues were fixed overnight in 4% paraformaldehyde, embedded in paraffin, and sectioned at a thickness of 4 μm. The liver sections were then blocked in a solution containing 10% FBS and 0.1% Triton-100 at 4 °C for 2 h. Subsequently, the sections were incubated with primary antibodies against MCM7, Albumin, α-SMA, and F4/80 at 4 °C for 24 h. After washing, the sections were incubated with fluorescent-labeled secondary antibodies at 4 °C for 2 h. The stained sections were then scanned using a TCS SP8 STED CW confocal microscope (Leica Microsystems, Wetzlar, Germany). The immunofluorescence signals were quantified using ImageJ software. The antibodies used in this study are listed in Supplemental Table 3.

### Enzyme-linked immunosorbent assay (ELISA)

The levels of IL11 in human and mouse serum samples were determined using the human IL11 ELISA Kit (CHE0010-096, 4A Biotech, Beijing, China) and the mouse IL11 ELISA Kit (CME0007-096, 4A Biotech, Beijing, China), respectively. The analyses were performed in accordance with the manufacturers’ instructions provided with the ELISA kits.

### Measurement of hepatic hydroxyproline content

The hepatic hydroxyproline content was quantified using a hydroxyproline assay kit (A030-2, Jiancheng Bioengineering Institute, Nanjing, China), following the manufacturer’s instructions. Data were provided as μg·100 mg^−1^ wet liver tissue.

### Statistics

All data are presented as the mean ± standard deviation (SD) from at least three independent experiments. The investigators were blinded to the group allocation during when assessing the outcome. The sample size was chosen to ensure adequate power to detect a prespecified effect size. Statistical analysis was performed using a 2-tailed Student’s *t*-test or 1-way or 2-way ANOVA, as appropriate. The correlation coefficient (r) was determined using Spearman’s rank correlation analysis. A *p*-value less than 0.05 was considered statistically significant.

## Results

### Upregulation of hepatic MCM7 expression by YAP in liver fibrosis

**Fig. 1:**
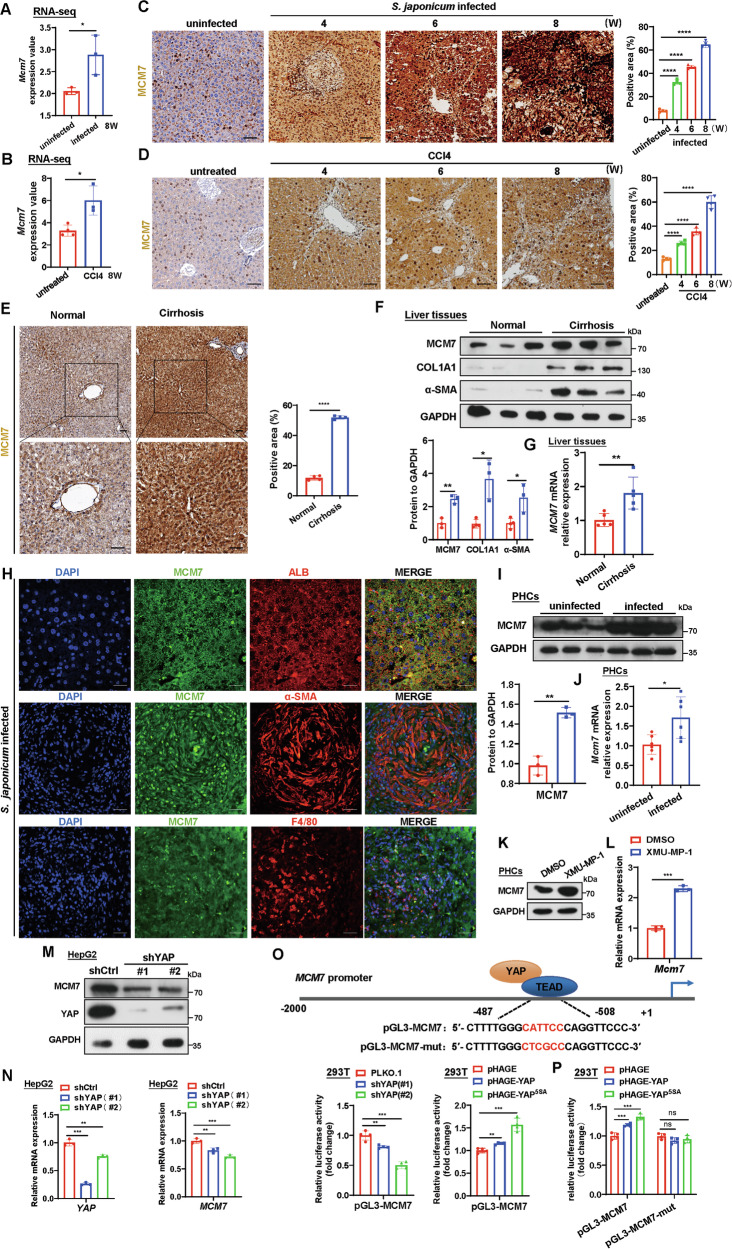
Upregulation of hepatic MCM7 expression driven by YAP activation in liver fibrosis. **A**, **B***Mcm7* transcript levels were assessed using two in-house RNA-seq data obtained from fibrotic mouse liver tissues following 8-week *S. japonicum* infection (**A**) or 8-week CCl4 treatment (**B**). **C**, **D** Immunohistochemical (IHC) analysis was performed to evaluate MCM7 expression were assessed in fibrotic liver tissues from mice infected with *S. japonicum* (**C**) or treated with CCl4 (**D**) for 4, 6, and 8 weeks (scale bar: 50 μm), with quantification of MCM7^+^ staining shown in the corresponding graphs. **E**–**G** MCM7 expression levels in human liver tissue samples from normal (*n* = 3) and cirrhosis (*n* = 3) groups, were assessed by IHC (**E**) (scale bar: 100 μm), with the graph showing the area of MCM7^+^ staining, Western blot (**F**) displaying protein levels, and qRT-PCR (**G**) determining mRNA expression. **H** Immunofluorescence (IF) analysis was performed to assess the colocalization of MCM7 in fibrotic liver sections from mice infected with *S. japonicum* for 8 weeks, with albumin (ALB), α-SMA, and F4/80 used to mark hepatocytes (HCs), hepatic stellate cells (HSCs), and Kupffer cells (KCs), respectively. Nuclei were stained with DAPI (scale bar: 50 μm). **I**, **J** MCM7 expression levels were assessed in primary hepatocytes (PHCs) isolated from uninfected mice and mice infected with *S. japonicum* for 8 weeks by Western blot (**I**), with the graph displaying protein levels, and by qRT-PCR (**J**). **K**, **L** Detection of protein (**K**) and mRNA (**L**) levels of MCM7 in PHCs after XMU-MP-1 stimulation for 24 h. **M**, **N** Detection of protein (**M**) and mRNA (**N**) levels of YAP and MCM7 in HepG2 cells transfected with shYAP. **O** The schematic diagram shows the *MCM7* promoter region with YAP binding sites, and relative luciferase activity of the *MCM7* promoter in HEK293T cells transfected with shYAP and pHAGE-YAP or pHAGE-YAP^5SA^. **P** Relative luciferase activity of the *MCM7* promoter in HEK293T cells transfected with pHAGE-YAP or pHAGE-YAP^5SA^, with or without mutations in the YAP binding site. Data are expressed as the mean ± SD of 3–6 mice per group and are representative of three independent experiments. Statistical analyses were performed using unpaired Student’s *t*-test or one-way ANOVA. *P < 0.05; **P < 0.01; ***P < 0.001; ****P < 0.0001; ns not significant.

To explore the potential role of MCM7 in liver fibrosis, we first analyzed *MCM7* transcript levels in normal and fibrotic liver samples from both patients and murine models. We utilized two independent microarray datasets from the Gene Expression Omnibus (GEO) database (GSE61376, GSE25713, and GSE55747), which include liver samples from patients infected with *S. japonicum* and from murine fibrosis models induced by *S. japonicum* and CCl4. Data analysis showed that *MCM7* mRNA expression was significantly upregulated in liver fibrosis tissues compared to normal tissues (Fig. S1A–C). Two in-house RNA-seq datasets generated by our research group-derived from fibrotic mouse liver tissues at advanced fibrosis stages induced by 8 weeks of *S. japonicum* infection or 8 weeks of CCl4 treatment-further confirmed the upregulation of *MCM7* mRNA expression in fibrotic liver tissues (Fig. 1A, B). Notably, immunohistochemistry, Masson’s trichrome staining, Western blot, and qRT-PCR analysis collectively demonstrated that MCM7 expression commenced increasing in *S. japonicum*-infected and CCl4-treated mouse livers at 4 weeks post-treatment and continued to rise up to 8 weeks, exhibiting a positive correlation with the progression of hepatic fibrosis (Figs. 1C, D, and S1D–H). To validate the clinical relevance of our findings, we analyzed cirrhotic human tissue samples and observed that both MCM7 mRNA and protein levels were significantly elevated in cirrhosis patients compared to healthy individuals (Fig. 1E–G). In addition, we isolated hepatocytes (HCs), HSCs, and Kupffer cells (KCs) from normal mouse livers and found that MCM7 is highly expressed in primary HCs compared to HSCs and KCs (Fig. S1I). Subsequently, double immunofluorescence staining of liver sections revealed that MCM7 primarily colocalized with albumin (a hepatocyte-specific marker) in both *S. japonicum*-infected (8 weeks post-infection) and CCl4-treated (6 weeks post-treatment) samples, indicating its predominant localization within hepatocytes during fibrosis (Figs. 1H and S1J). In contrast, MCM7 was also detected in α-SMA-positive HSCs (Figs. 1H and S1J). Moreover, we isolated primary hepatocytes from *S. japonicum*- and CCl4-induced mouse fibrotic livers to assess MCM7 expression at both mRNA and protein levels. Consistent with our immunofluorescence data, we observed significant upregulation of both MCM7 mRNA and protein levels in hepatocytes from mice treated with *S. japonicum* or CCl4 compared to controls (Figs. 1I, J and S1K, L). These findings collectively suggest a significant upregulation of MCM7 within hepatocytes during the progression of liver fibrosis.

Having confirmed the upregulation of MCM7 in liver fibrosis, we next aimed to identify potential extracellular activators of *MCM7* gene transcription. Previous studies have shown that during the progression of liver fibrosis, inactivation of the Hippo signaling pathway leads to the persistent upregulation of YAP expression [25]. Additionally, YAP activates the transcription of *MCM7* in non-small cell lung cancer [14]. To explore whether YAP activation drives the increased expression of MCM7 in liver fibrosis, we treated primary hepatocytes and HepG2 cells with XMU-MP-1, an inhibitor of the Hippo signaling pathway. XMU-MP-1 treatment significantly increased both mRNA and protein levels of MCM7 in these cells (Figs. 1K, L, and S2A, B). Subsequently, we observed that YAP knockdown reduced, whereas overexpression of YAP and its constitutively active mutant YAP^5SA^ [26] significantly elevated MCM7 mRNA and protein levels in HepG2 cells (Figs. 1M, N and S2C, D). To further investigate the potential transcriptional regulation of *MCM7* by YAP, we generated the wild-type *MCM7* promoter-luciferase reporter construct covering the potential YAP binding and a mutated version. YAP knockdown inhibited *MCM7* promoter activity, while overexpression of YAP and the YAP^5SA^ mutant significantly enhanced it (Fig. 1O). Luciferase reporter assays showed that mutating the YAP binding sites in the *MCM7* promoter blocked the upregulation of promoter activity induced by both YAP overexpression and the YAP^5SA^ mutant (Fig. 1P). These findings indicate that YAP activates the transcription of *MCM7* in hepatocytes.

### Hepatocyte MCM7 knockdown alleviates liver fibrosis in both *S. japonicum*- and CCl4-induced mice

**Fig. 2:**
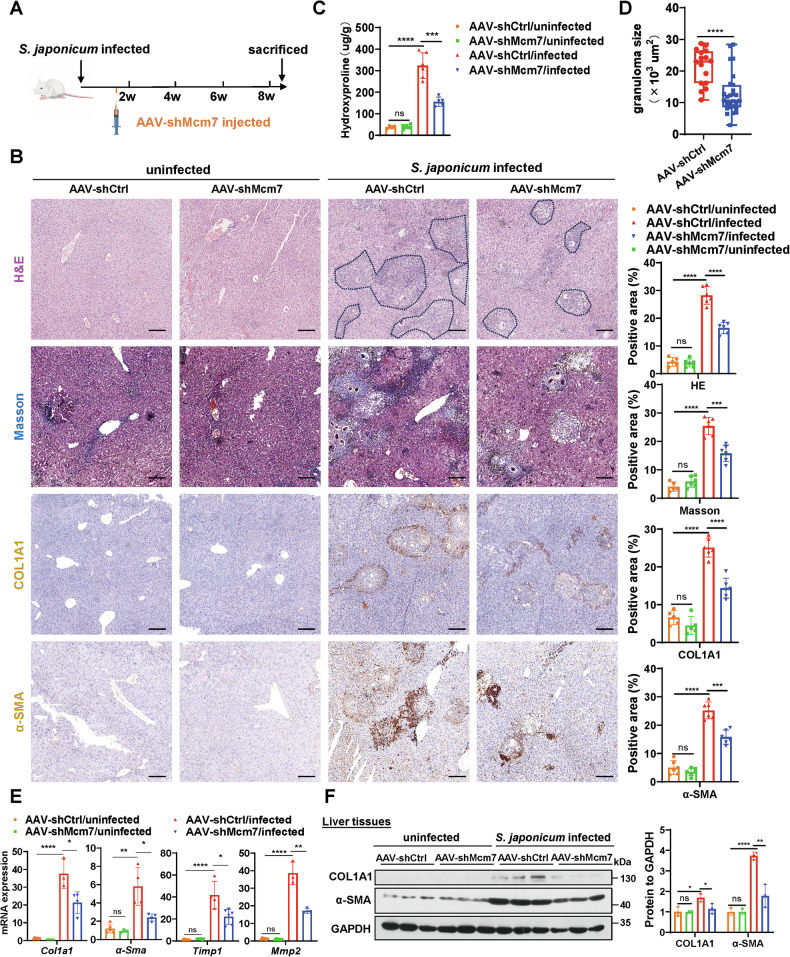
Hepatocyte MCM7 knockdown attenuates liver fibrosis in *S. japonicum*-induced mice. **A** Experimental design schematic: mice were infected with *S. japonicum* and received intravenous injections of either shCtrl or AAV-shMcm7 on day 10 post-infection. Liver samples were collected at 8 weeks post-infection for analysis. **B** H&E staining (areas positive for liver fibrosis are delineated by black dashed lines), Masson’s trichrome staining, COL1A1 staining, and α-SMA staining (all scale bars: 100 μm) of liver sections from the indicated groups (AAV-shCtrl/uninfected, AAV-shMcm7/uninfected, AAV-shCtrl/infected, AAV-shMcm7/infected). Graphs show the quantified positive areas for each stain, determined using ImageJ software from multiple randomly selected fields across distinct tissue sections. **C** Hydroxyproline content in liver tissues was determined. **D** The size of the granuloma area in *S. japonicum*-induced mice (AAV-shCtrl/infected, AAV-shMcm7/infected) was measured and calculated. **E**, **F** qRT-PCR (**E**) was used to assess the expression levels of *Col1a1*, *α-Sma*, *Timp1*, and *Mmp2*, while Western blot (**F**) analysis focused on COL1A1 and α-SMA in liver tissues from the indicated groups, with the graph displaying protein levels. Data are presented as the mean ± SD of 3–6 mice per group and are representative of three independent experiments. Statistical analyses were performed using an unpaired Student’s *t*-test or one-way ANOVA. *P < 0.05; **P < 0.01; ***P < 0.001; ****P < 0.0001; ns not significant.

To explore the potential impact of targeting MCM7 in hepatocytes on established liver fibrosis, we employed an adeno-associated virus serotype 8 (AAV8) vector to knockdown MCM7 in *S. japonicum*-induced liver fibrosis models. AAV8 vectors exhibited high transduction efficiency in hepatocytes, demonstrating preferential targeting of hepatocytes (Fig. S3A). As shown in Fig. 2A, mice were first infected with a mild dose of *S. japonicum* cercariae, followed by AAV-shMcm7 or AAV-shCtrl injection at day 10 post-infection. MCM7 knockdown was confirmed by qRT-PCR and Western blot analysis (Fig. S3B). Additionally, the health status of the mice post-treatment showed that they tolerated MCM7 targeting well (Supplementary Table 4). Hematoxylin and eosin (H&E), Masson’s trichrome staining, and immunohistochemistry revealed that hepatocyte MCM7 knockdown in *S. japonicum*-induced mice significantly attenuated hepatic fibrosis compared to controls (Fig. 2B). This was further confirmed by a significant decrease in the level of hydroxyproline and the area of egg granuloma (Fig. 2C, D). Although granuloma quantification is specific to *S. japonicum*-induced fibrosis, the size of hepatic granulomas following *schistosome* infection is closely correlated with the progression of hepatic fibrosis [27]. Furthermore, qRT-PCR and Western blot analysis revealed reduced expression of fibrosis markers, including alpha-smooth muscle actin (α-SMA), collagen type I alpha 1 (COL1A1), tissue inhibitor of metalloproteinase 1 (TIMP1), and matrix metalloproteinase-2 (MMP2) in the livers of MCM7 knockdown mice following *S. japonicum* infection, compared to controls (Fig. 2E, F).

**Fig. 3:**
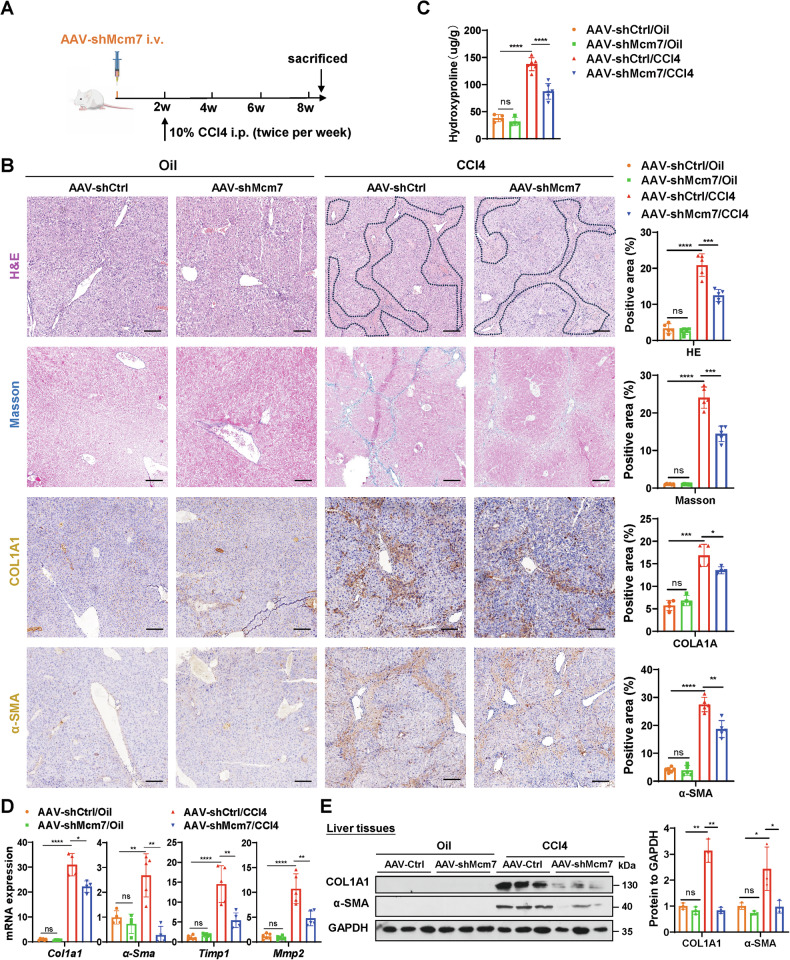
Hepatocyte MCM7 knockdown attenuates liver fibrosis in CCl4-treated mice. **A** Experimental design schematic: mice were intravenously injected with shCtrl or AAV-shMcm7. Two weeks after the AAV injection, mice began receiving intraperitoneal injections of 10% CCl4 twice per week. Liver samples were collected at 6 weeks post-CCl4 treatment for analysis. **B** H&E staining (areas positive for liver fibrosis are delineated by black dashed lines), Masson’s trichrome staining, COL1A1 staining, and α-SMA staining (all scale bars: 100 μm) of liver sections from the indicated groups (AAV-shCtrl/Oil, shMcm7/Oil, AAV-shCtrl/CCl4, AAV-shMcm7/CCl4). Graphs show the quantified positive areas for each stain, determined using ImageJ software from multiple randomly selected fields across distinct tissue sections. **C** Hydroxyproline content in liver tissues was determined. **D**, **E** qRT-PCR (**D**) assessed the expression levels of *Col1a1*, *α-Sma*, *Timp1*, and *Mmp2*, while Western blot (**E**) analysis focused on COL1A1 and α-SMA in liver tissues from the indicated groups, with the graph displaying protein levels. Data are presented as the mean ± SD of 3–6 mice per group and are representative of three independent experiments. Statistical analyses were performed using an unpaired Student’s *t*-test or one-way ANOVA. *P < 0.05; **P < 0.01; ***P < 0.001; ****P < 0.0001; ns not significant.

Additionally, the CCl4-induced liver fibrosis model, a widely used model, was employed to further investigate the role of hepatocyte MCM7 in liver fibrosis. Results showed that hepatocyte MCM7 knockdown in CCl4-induced mice exhibited reduced fibrosis, as indicated by histopathology (H&E and Masson’s trichrome staining), immunohistochemistry, hydroxyproline content, and hepatic fibrosis marker gene quantification (Figs. 3A–E and S3C). These data suggest that MCM7 knockdown alleviates liver fibrosis in both *S. japonicum*- and CCl4-induced mouse models.

### Hepatocyte overexpression of MCM7 aggravates *S. japonicum*- and CCl4-induced liver fibrosis in mice

We further utilized AAV8 vectors to drive MCM7 overexpression (AAV-MCM7), thereby generating hepatocyte-specific MCM7 overexpression in *S. japonicum*-induced liver fibrosis mice. The experimental design is illustrated in Fig. S4A. MCM7 overexpression was confirmed in AAV-MCM7-injected mice by qRT-PCR and Western blot analysis (Fig. S3D). H&E staining, Masson’s trichrome staining, immunohistochemistry, hydroxyproline content, the area of egg granuloma, qRT-PCR, and Western blot analysis of hepatic fibrosis markers collectively revealed that hepatocyte MCM7 overexpression significantly aggravated hepatic fibrosis in *S. japonicum*-infected mice (Fig. S4B–F). Similar results were also observed in the CCl4-induced liver fibrosis model (Figs. S3E and S5A–E). Taken together, these results indicate that hepatocyte MCM7 overexpression aggravates liver fibrosis in both *S. japonicum*- and CCl4-induced mouse models.

### MCM7 physically interacts with SHCBP1

**Fig. 4:**
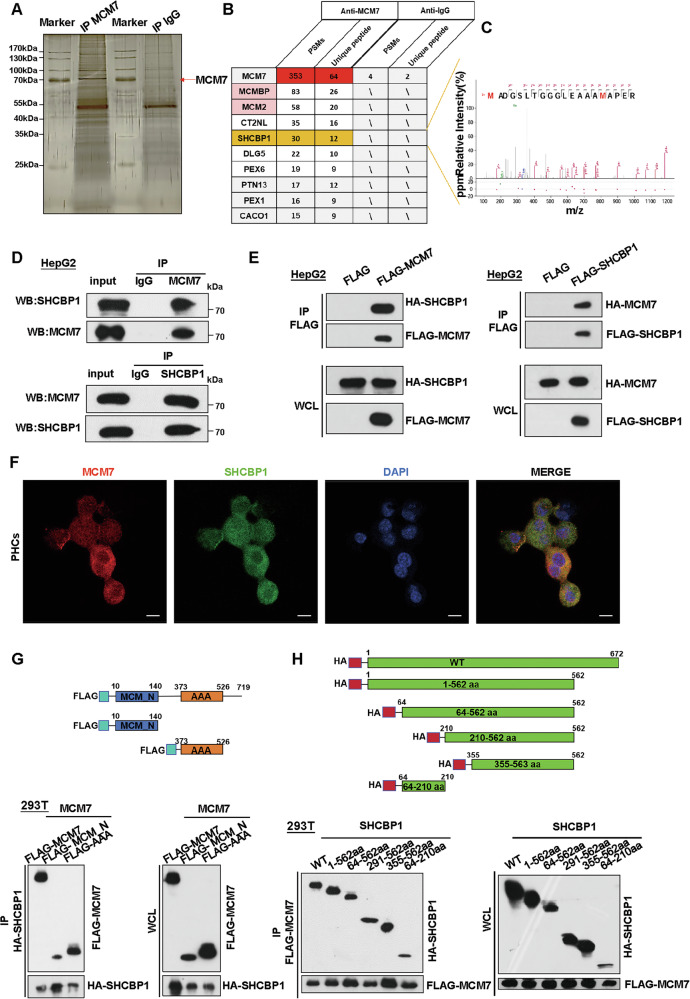
Interaction analysis of MCM7 and SHCBP1. **A** Silver staining was used to visualize interacting proteins with MCM7, which were enriched using an MCM7 antibody. The arrowhead indicates the predicted size of MCM7. **B** Distinct protein bands from the gel were subjected to mass spectrometry, and the top ten interacting partners are shown. **C** The peptide map of the marked protein band identifies it as SHCBP1. **D** Endogenous interaction between MCM7 and SHCBP1 was detected by Co-IP in HepG2 cells. **E** Exogenous interaction between FLAG/HA-tagged MCM7 and FLAG/HA-tagged SHCBP1 was validated by Co-IP in HepG2 cells. **F** Immunofluorescence (IF) analysis showing the colocalization of MCM7 and SHCBP1 in primary hepatocytes (PHCs) (scale bar: 5 μm). **G**, **H** Schematic diagrams of MCM7 (**G**) and SHCBP1 (**H**) truncations (top), and Co-IP assays analyzing the interaction domains of MCM7 and SHCBP1 (bottom).

To investigate the mechanism by which hepatic MCM7 influences liver fibrosis pathogenesis, we performed immunoprecipitation using an anti-MCM7 antibody in a hepatocyte cell line. The immunoprecipitated complexes were subsequently subjected to silver staining to visualize protein bands (Fig. 4A). After visualization, the samples were analyzed by mass spectrometry to identify potential binding partners of MCM7. This analysis identified 152 protein candidates showing at least a twofold enrichment compared to the IgG control (Supplementary Table 5), and the top ten differentially expressed proteins are shown in Fig. 4B. Among these, SHCBP1 emerged as a key candidate, acting as a SHC SH2-domain binding protein that regulates multiple signaling pathways, including FGF, NF-κB, MAPK/ERK, PI3K/AKT, TGF-β1/Smad, and Wnt/β-catenin, suggesting its crucial and consistent role in liver fibrosis pathogenesis [28]. The peptide spectrum reflects the abundance of SHCBP1 peptides (Fig. 4C). Western blot analysis was conducted to validate its expression in hepatic tissues and hepatocyte cell lines (Fig. S6A, B). Additionally, Western blot analysis demonstrated that knockdown of either MCM7 or SHCBP1 had no effect on the expression of the other protein in HepG2 cells (Fig. S6C). To validate the interaction between MCM7 and SHCBP1, we first performed an endogenous Co-IP assay in HepG2 cells, which confirmed the interaction (Fig. 4D). We then performed Co-IP assays in HepG2 and HEK293T cells overexpressing MCM7 or SHCBP1, further supporting the protein–protein interaction (Figs. 4E and S6D). Confocal analysis further demonstrated the colocalization of MCM7 and SHCBP1 in primary hepatocytes (Fig. 4F). Furthermore, to map the interaction domains, truncated MCM7 fragments containing the N-terminal domain (amino acids 10–140) and the AAA domain (amino acids 373–526) demonstrated binding affinity for SHCBP1 (Fig. 4G). Similarly, serial truncations of SHCBP1 (amino acids 1–672, 1–562, 64–562, 291–562, 355–562, and 64–210) and MCM7 (full length) were generated and co-expressed in HEK293T cells. The results show that all truncated SHCBP1 fragments exhibited binding capability with MCM7 (Fig. 4H). All truncated fragments of MCM7 and SHCBP1 exhibited mutual binding affinity, further confirmed by GST/His pull-down assays, which supports their direct interaction in an in vitro system (Fig. S6E, F). Collectively, these results validate the physical interaction between MCM7 and SHCBP1.

### Hepatocyte SHCBP1 promotes liver fibrosis

Given the critical role of MCM7 in liver fibrosis pathogenesis and its interaction with SHCBP1, we analyzed gene expression datasets (GSE61376, GSE25713, and GSE55747) and our RNA-seq data from *S. japonicum*- and CCl4-induced liver fibrosis models. The analysis revealed significant upregulation of *SHCBP1* mRNA (Fig. S7A–E). qRT-PCR and Western blot analyses showed a significant upregulation of SHCBP1 expression in liver fibrosis in both patients and mouse models (Fig. S7F–J). The upregulation of SHCBP1 mRNA and protein expression was also confirmed in primary hepatocytes isolated from liver fibrosis models (Fig. S7K–N). Additionally, we found that increased hepatic *SHCBP1* mRNA expression is significantly correlated with elevated *MCM7* mRNA levels in liver fibrosis (Fig. S7O, P). In conclusion, our findings indicate that SHCBP1 is significantly upregulated in liver fibrosis and correlates with increased MCM7 expression.

**Fig. 5:**
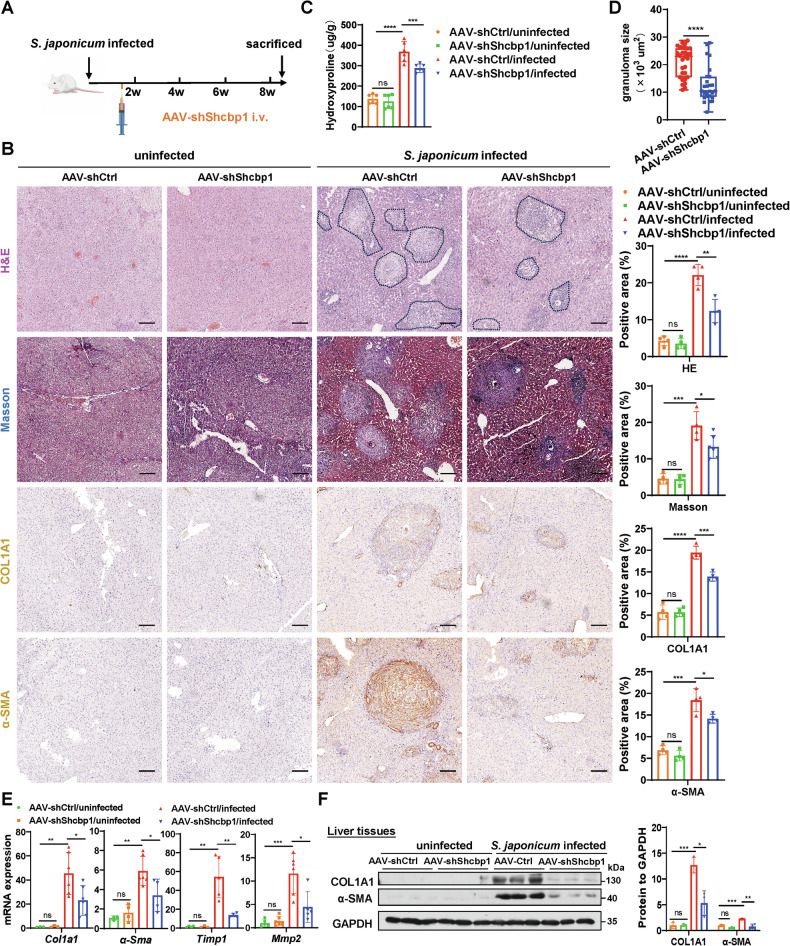
Hepatocyte SHCBP1 knockdown attenuates liver fibrosis in *S. japonicum* -induced mice. **A** Experimental design schematic: mice were infected with *S. japonicum* and received intravenous injections of either AAV-shCtrl or AAV-shShcbp1 on day 10 post-infection. Liver samples were collected at 8 weeks post-infection for analysis. **B** H&E staining (areas positive for liver fibrosis are delineated by black dashed lines), Masson’s trichrome staining, COL1A1 staining, and α-SMA staining (all scale bars: 100 μm) of liver sections from the indicated groups (AAV-shCtrl/uninfected, AAV-shShcbp1/uninfected, AAV-shCtrl/infected, AAV-shShcbp1/infected). Graphs show the quantified positive areas for each stain, determined using ImageJ software from multiple randomly selected fields across distinct tissue sections. **C** Hydroxyproline content in liver tissues was determined. **D** The size of the granuloma area in *S. japonicum*-induced mice (AAV-shCtrl/infected, AAV-shShcbp1/infected) was measured and calculated. **E**, **F** qRT-PCR (**E**) was used to assess the expression levels of *Col1a1*, *α-Sma*, *Timp1*, and *Mmp2*, while Western blot (**F**) analysis focused on COL1A1 and α-SMA in liver tissues from the indicated groups, with the graph displaying protein levels. Data are presented as the mean ± SD of 3–6 mice per group and are representative of three independent experiments. Statistical analyses were performed using an unpaired Student’s *t*-test or one-way ANOVA. *P < 0.05; **P < 0.01; ***P < 0.001; ****P < 0.0001; ns not significant.

To investigate the function of SHCBP1 in liver fibrosis, we constructed an AAV8 vector to knock down SHCBP1 in hepatocytes of liver fibrosis models. SHCBP1 knockdown was confirmed in AAV-shShcbp1-injected mice (Fig. S8A, B). Histological examination, hydroxyproline content analysis, egg granuloma area measurement, qRT-PCR, and Western blot results all indicated that fibrosis severity was ameliorated following AAV-shShcbp1 treatment (Figs. 5A–F and S9A–E). Conversely, in hepatocyte-specific SHCBP1 overexpression liver fibrosis models, we observed contrasting results (Figs. S8C, D, S10A–F, and S11A–E). Collectively, these findings demonstrate that hepatocyte-derived SHCBP1 promotes liver fibrosis progression in mice.

### MCM7 activates IL11 expression through interaction with SHCBP1

**Fig. 6:**
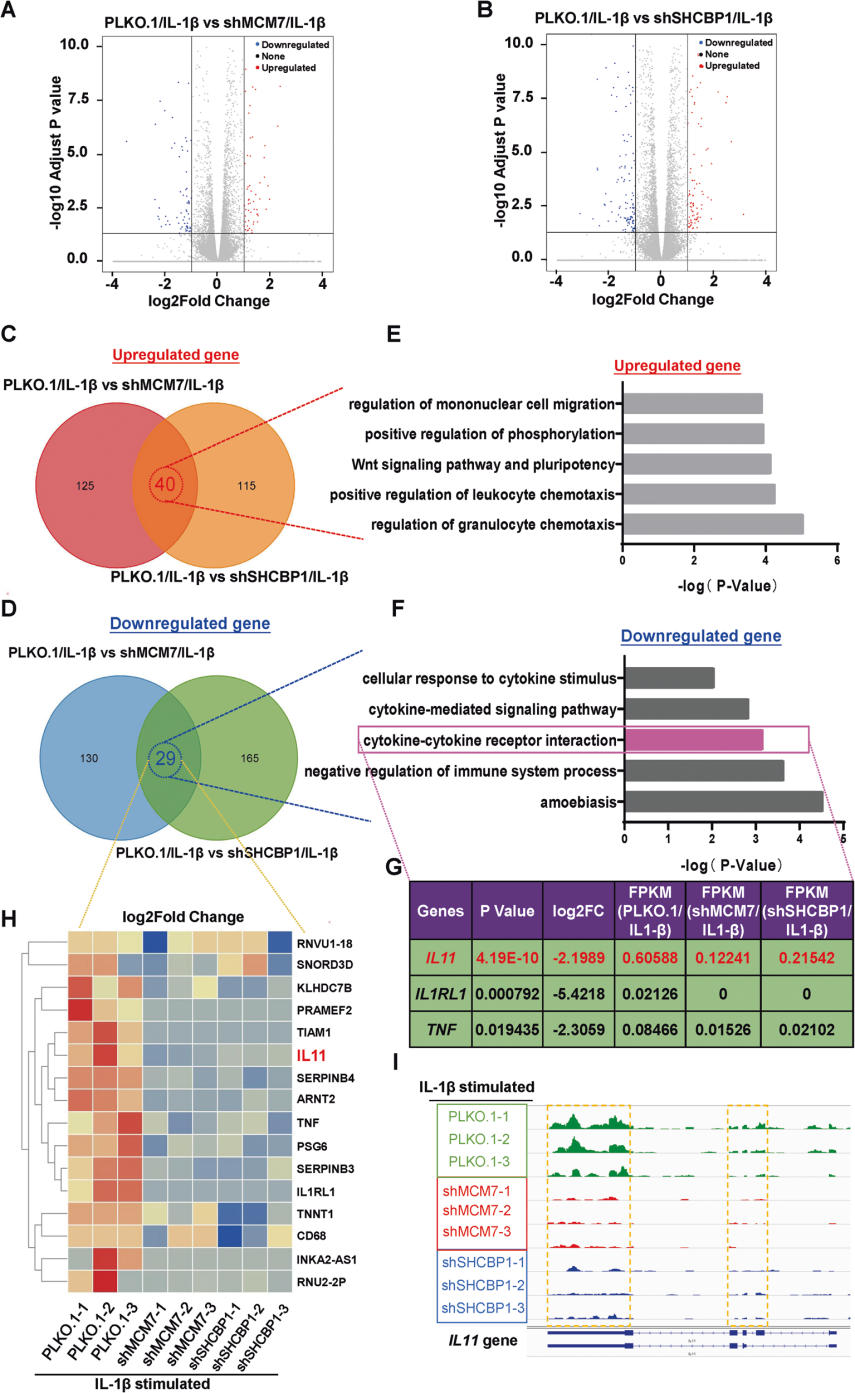
RNA-seq analysis in MCM7 and SHCBP1 knockdown cells upon IL-1β stimulation. **A**, **B** Volcano plots of differentially expressed genes in MCM7-knockdown (**A**) or SHCBP1-knockdown (**B**) cells treated with IL-1β. Upregulated genes are highlighted in red, and downregulated genes in blue. **C**, **D** Venn diagrams illustrating the overlap of upregulated (**C**) and downregulated (**D**) differentially expressed genes between MCM7-knockdown and SHCBP1-knockdown cells upon IL-1β treatment. **E**, **F** Pathway analysis of the upregulated overlapping genes (**E**) and downregulated overlapping genes (**F**). **G** Table listing significantly differentially expressed genes from the cytokine-cytokine receptor interaction network. **H** Heatmap depicting the downregulated expression of genes in both MCM7-knockdown and SHCBP1-knockdown cells upon IL-1β treatment. **I** Genome browser tracks of RNA-Seq signals at the *IL11* gene in PLKO.1, MCM7-knockdown, and SHCBP1-knockdown cells after IL-1β treatment.

To investigate the potential regulatory network of MCM7 and SHCBP1 in liver fibrosis pathogenesis, RNA-seq was performed on stable MCM7 or SHCBP1 knockdown hepatocytes treated with IL-1β, an inflammatory stimulus known to be highly expressed in liver fibrosis [29]. The knockdown of MCM7 or SHCBP1 was confirmed using qRT-PCR and Western blot (Fig. S12A, B). It has been reported that MCM7 regulates the splicing of EGFR and PDGFR in human prostate cancer cells [19]. However, our splicing analysis revealed that knockdown of MCM7 had no impact on RNA splicing (Fig. S12C). After knockdown of MCM7 or SHCBP1, the expression of 165 and 155 genes, respectively, was significantly increased in cells following IL-1β stimulation, whereas that of 159 and 194 mRNAs, respectively, was decreased (Fig. 6A, B). Interestingly, 40 of the genes upregulated by MCM7 knockdown also showed increased expression in SHCBP1 knockdown cells. In addition, the expression of 29 genes was decreased in both MCM7- and SHCBP1-knockdown cells after IL-1β treatment (Fig. 6C, D). Pathway analysis of DEGs revealed that upregulated genes were primarily enriched in regulation of granulocyte chemotaxis, positive regulation of leukocyte chemotaxis, Wnt signaling pathway and pluripotency, positive regulation of phosphorylation, and regulation of mononuclear cell migration (Fig. 6E), whereas the downregulated genes were enriched in functions such as cytokine-cytokine receptor interaction, cytokine-mediated signaling pathway, cellular response to cytokine stimulus, amoebiasis, and negative regulation of immune system process (Fig. 6F). Cytokine-cytokine receptor interaction is known to be associated with pathogenesis of liver fibrosis [5]. Among the components of the cytokine-cytokine receptor interaction, IL11, TNF, and IL1RL1 were identified, with IL11 showing the most significant decrease (Fig. 6G). Previous studies have shown that IL11 released from hepatocytes plays an important role in the development of liver fibrosis [6, 30]. Interestingly, we found that IL11 expression was elevated in liver tissues and hepatocytes during liver fibrosis induced by *S. japonicum* or CCl4 (Fig. S13A–I). Additionally, both the heatmap and Integrative Genomics Viewer showed the downregulation of *IL11* gene expression following knockdown of MCM7 or SHCBP1 (Fig. 6H, I). Consistently, we found decreased IL11 levels in liver tissues from MCM7 or SHCBP1 knockdown mice following *S. japonicum* or CCl4 treatment (Fig. S14A). Conversely, overexpression of MCM7 or SHCBP1 significantly elevated IL11 levels (Fig. S14B). Furthermore, in liver cirrhosis patients, a positive correlation was observed between the expression of *MCM7* or *SHCBP1* mRNA and increased IL11 expression (Fig. S14C). To investigate whether the *IL11* gene is transcriptionally regulated by MCM7 or SHCBP1, we constructed a luciferase reporter with a 2 kb upstream sequence of the *IL11* gene promoter. MCM7 knockdown led to a significant decrease in *IL11* promoter activity, while MCM7 overexpression increased it (Fig. S14D). Similarly, SHCBP1 knockdown also decreased *IL11* promoter activity, and SHCBP1 overexpression increased it (Fig. S14E). Notably, SHCBP1 overexpression rescued the decrease in *IL11* promoter activity induced by MCM7 knockdown (Fig. S14F). Collectively, these results demonstrate that MCM7 activates *IL11* expression via its interaction with SHCBP1.

To further validate that MCM7 activates *IL11* gene expression, we performed RNA-seq on primary hepatocytes isolated from AAV-shMcm7-injected mice. Specifically, the mice were treated with AAV-shMcm7 or AAV-shCtrl for 2 weeks via tail-vein injection. The isolated hepatocytes were then treated with recombinant mouse IL-6 to mimic the inflammatory environment characteristic of liver fibrosis. We observed an increase in the expression of 3327 genes and a decrease in the expression of 5290 genes following MCM7 knockdown upon IL-6 stimulation (Fig. S15A). Pathway analysis of the DEGs in primary hepatocytes showed significant enrichment of downregulated cytokine-cytokine receptor interaction, which is consistent with our previous findings in stable MCM7 knockdown cells treated with IL-1β (Fig. S15B, C). Notably, heatmap analysis revealed a significant decrease in *IL11* gene expression in MCM7 knockdown primary hepatocytes (Fig. S15D). Further validation by qRT-PCR demonstrated that *Il11* mRNA expression was significantly decreased in MCM7 knockdown primary hepatocytes and increased in MCM7 overexpression primary hepatocytes following IL-6 treatment (Fig. S15E, F). In conclusion, these findings confirm that MCM7 drives *IL11* gene expression.

### MCM7 activates hepatic IL11 expression via the SHCBP1-RACGAP1-STAT3 signaling axis

**Fig. 7:**
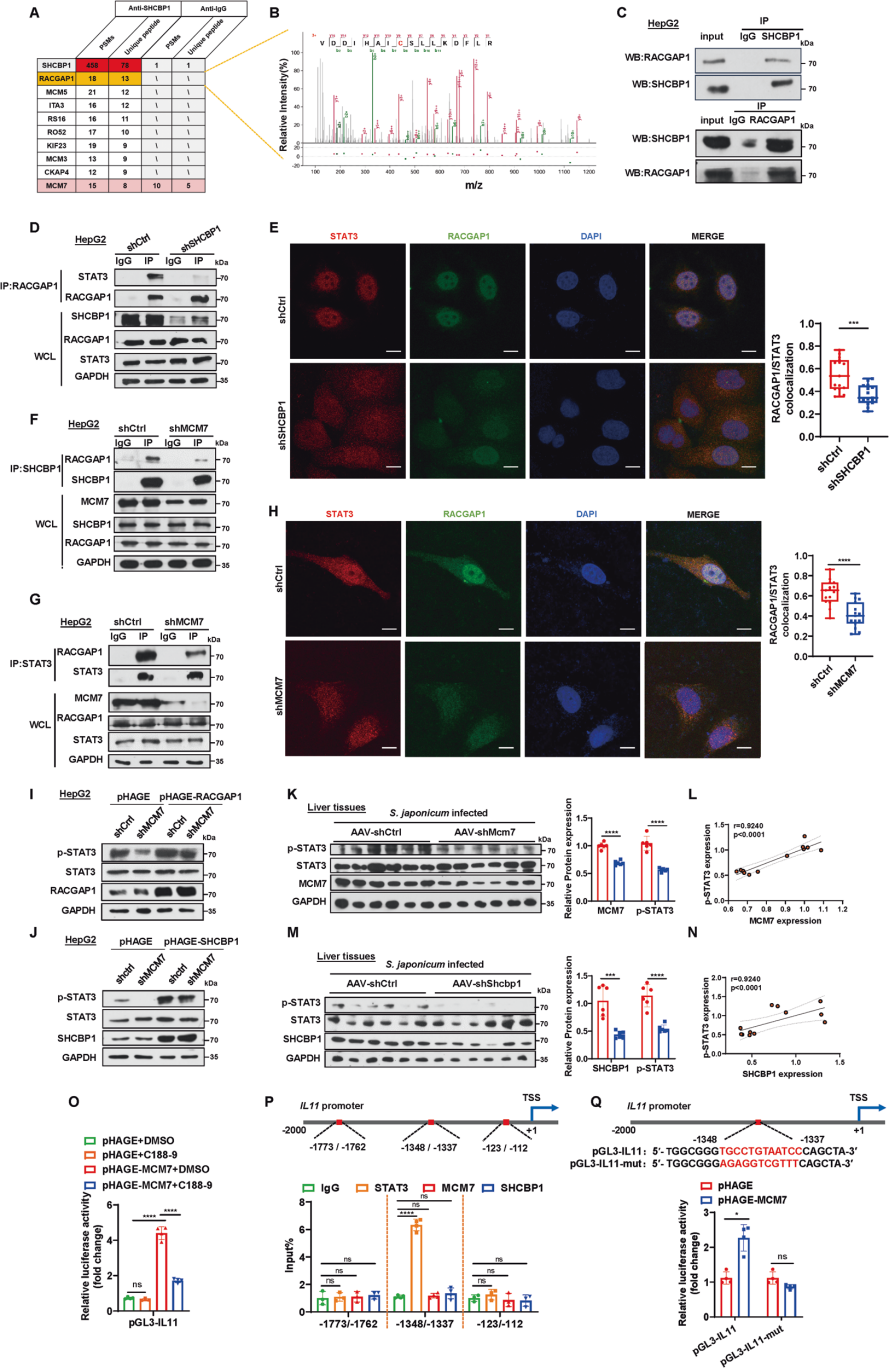
MCM7 activates hepatic IL11 expression through SHCBP1-RACGAP1-STAT3 axis. **A** Identification of SHCBP1-interacting proteins by mass spectrometry, with the top ten interacting partners shown. **B** The peptide map from the marked protein band identifies RACGAP1 as a SHCBP1-interacting protein. **C** Co-IP analysis of the endogenous interaction between SHCBP1 and RACGAP1 in HepG2 cells. **D** Co-IP showing the interaction between RACGAP1 and STAT3 in HepG2 cells transfected with shCtrl or shSHCBP1. **E** Immunofluorescence (IF) showing the colocalization of STAT3 (red), RACGAP1 (green), and DAPI (nucleus, blue) in HepG2 cells transfected with shCtrl or shSHCBP1 (scale bar: 5 μm). The graph presents quantitative analysis of RACGAP1/STAT3 colocalization. **F**, **G** Co-IP analysis showing the interaction between SHCBP1 and RACGAP1 (**F**) or between RACGAP1 and STAT3 (**G**) in HepG2 cells transfected with shCtrl or shMCM7. **H** IF showing the colocalization of STAT3 (red), RACGAP1 (green), and DAPI (nucleus, blue) in HepG2 cells transfected with shCtrl or shMCM7 (scale bar: 5 μm). The graph presents quantitative analysis of RACGAP1/STAT3 colocalization. **I**, **J** Western blot analysis of phosphorylated STAT3 (p-STAT3) in HepG2 cells with MCM7 knockdown and co-transfected with pHAGE-RACGAP1 (**I**) or pHAGE-SHCBP1 (**J**). **K**, **M** Western blot analysis of p-STAT3 in liver tissues from *S. japonicum*-infected mice treated with AAV-shMcm7 (**K**) or AAV-shShcbp1 (**M**). Graphs show the relative protein expression levels. **L**, **N** Correlation between p-STAT3 levels and MCM7 expression (**L**) or SHCBP1 expression (**N**) in liver tissues from *S. japonicum*-infected mice (*n* = 12). **O** Relative luciferase activity of the *IL11* promoter in HepG2 cells transfected with pHAGE or pHAGE-MCM7 with or without C188-9. **P** The schematic diagram shows the *IL11* promoter region indicating STAT3 binding sites, and ChIP-qPCR analysis of STAT3, MCM7, and SHCBP1 binding to the *IL11* promoter was conducted in HepG2 cells. **Q** Relative luciferase activity of the *IL11* promoter in HepG2 cells transfected with pHAGE or pHAGE-MCM7, with or without mutations in the STAT3 binding site (−1348/−1337). Data are presented as mean ± SD of three independent experiments. Statistical analyses were performed using unpaired Student’s *t*-test, one-way ANOVA. *P < 0.05; ***P < 0.001; ****P < 0.0001; ns not significant.

To elucidate the molecular mechanisms by which MCM7 activates *IL11* expression via its interaction with SHCBP1, we performed immunoprecipitation with an anti-SHCBP1 antibody followed by mass spectrometry to identify potential binding partners of SHCBP1 in a hepatocyte cell line. (Supplementary Table 6). The interaction between SHCBP1 and MCM7 was confirmed as well (Fig. 7A). Notably, RACGAP1 was identified as a major partner of SHCBP1 (Fig. 7A), with its peptide spectrum displayed in Fig. 7B. We then conducted a Co-IP assay to verify the interaction between RACGAP1 and SHCBP1 in HepG2 cells (Fig. 7C). RACGAP1 is known to be a crucial mediator of STAT3 tyrosine phosphorylation and functions as a chaperone for the nuclear translocation of STAT3 [31]. Furthermore, SHCBP1 knockdown suppressed the interaction between RACGAP1 and STAT3 in Co-IP assays (Fig. 7D). Immunofluorescence analysis showed that SHCBP1 knockdown reduced STAT3 nuclear import and decreased colocalization of RACGAP1 and STAT3 (Fig. 7E). These findings reinforce the idea that SHCBP1 facilitates the binding of RACGAP1 to STAT3 and regulates its nuclear import. Considering that MCM7 interacts with SHCBP1 and SHCBP1 facilitates the interaction between RACGAP1 and STAT3, we hypothesized that MCM7 might modulate the interaction between RACGAP1 and STAT3 via SHCBP1. To test this hypothesis, we performed Co-IP experiments and found that MCM7 knockdown impaired the interaction between SHCBP1 and RACGAP1 (Fig. 7F). Moreover, the binding of RACGAP1 to STAT3 was decreased in MCM7 knockdown cells (Fig. 7G). Immunofluorescence analysis demonstrated that MCM7 knockdown led to decreased STAT3 nuclear import and reduced colocalization with RACGAP1 (Fig. 7H). Notably, overexpression of either RACGAP1 or SHCBP1 rescued the MCM7 knockdown-induced downregulation of STAT3 phosphorylation (Fig. 7I, J). Additionally, further analysis revealed a positive correlation between MCM7 expression and STAT3 phosphorylation in murine liver fibrosis models (Fig. 7K, L). Similarly, SHCBP1 expression positively correlated with STAT3 phosphorylation (Fig. 7M, N). Taken together, these results suggest that MCM7 modulates the interaction between RACGAP1 and STAT3 through SHCBP1, thereby enhancing STAT3 phosphorylation and nuclear translocation.

To further investigate whether MCM7 activates *IL11* transcription through STAT3 regulation, we conducted luciferase reporter assays using the *IL11* promoter. Treatment with C-118-9, a STAT3 inhibitor, significantly suppressed the luciferase activity of the *IL11* promoter in MCM7-overexpressing hepatocytes (Fig. 7O). Additionally, predictive analysis using the JASPAR database identified three potential STAT3 binding sites within the *IL11* promoter region. ChIP-qPCR assays confirmed significant STAT3 enrichment specifically in the −1348/−1337 bp region of the *IL11* promoter (Fig. 7P). In addition, to investigate whether SHCBP1 or MCM7 directly binds to the *IL11* promoter, we performed ChIP-qPCR assays using antibodies against SHCBP1 and MCM7. Our results demonstrated that neither SHCBP1 nor MCM7 was recruited to the *IL11* promoter (Fig. 7P). This finding indicates that SHCBP1 and MCM7 do not directly bind to the *IL11* promoter to regulate its transcription. Moreover, we generated luciferase reporter constructs with mutations in the STAT3 binding site in the −1348/−1337 region of the *IL11* promoter. The mutation significantly blocked the upregulation of luciferase activity induced by MCM7 overexpression (Fig. 7Q). In summary, these results demonstrate MCM7 facilitates the interaction between RACGAP1 and STAT3 through SHCBP1, thereby promoting STAT3 binding to the *IL11* promoter.

### Inhibition of IL11 attenuates MCM7 overexpression-induced liver fibrosis by inhibiting HSC activation

**Fig. 8:**
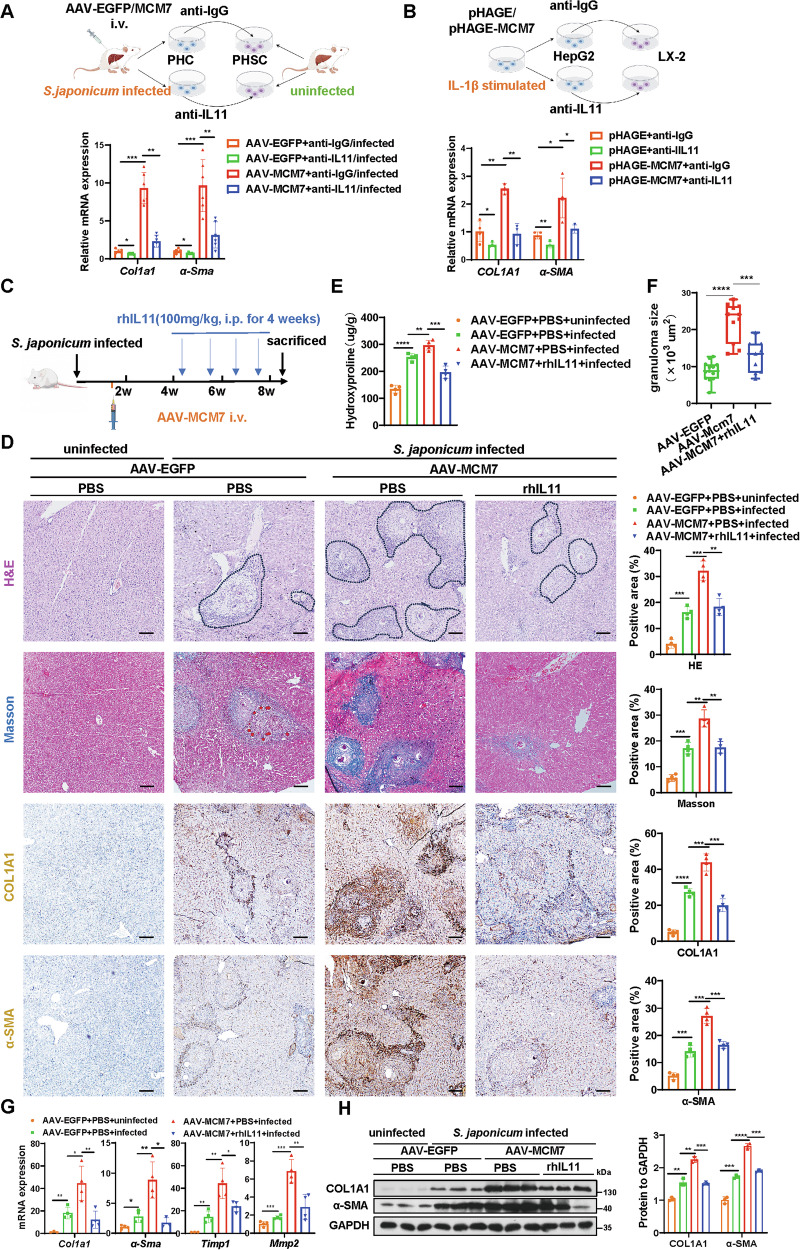
Inhibition of IL11 attenuates MCM7 overexpression-induced liver fibrosis by inhibiting HSC activation. **A** Experimental design schematic: HSCs isolated from uninfected mice were exposed to conditioned medium from primary hepatocytes of *S. japonicum*-infected mice treated with AAV-EGFP or AAV-MCM7, with or without IL11 neutralizing antibody (top). Relative mRNA expression levels of *Col1a1* and *α-Sma* in HSCs were analyzed by qRT-PCR (bottom). **B** Experimental design schematic: LX-2 cells were exposed to conditioned medium from HepG2 cells transfected with pHAGE or pHAGE-MCM7, with or without IL11 neutralizing antibody (top). Relative mRNA expression levels of *COL1A1* and *α-SMA* in LX-2 cells were analyzed by qRT-PCR (bottom). **C** Experimental design schematic: mice were infected with *S. japonicum* and intravenously injected with either AAV-MCM7 or AAV-EGFP on day 10 post-infection. Four weeks before sacrifice, mice were intraperitoneally treated rhIL11 every two days. Liver samples were collected at 8 weeks post-infection for analysis. **D** H&E staining (areas positive for liver fibrosis are delineated by black dashed lines), Masson’s trichrome staining, COL1A1 staining, and α-SMA staining (all scale bars: 100 μm) of liver sections from the indicated groups (AAV-EGFP + PBS + uninfected, AAV-EGFP + PBS + infected, AAV-MCM7 + PBS + infected, AAV-MCM7 + rhIL11 + infected). Graphs show the quantified positive areas for each stain, determined using ImageJ software from multiple randomly selected fields across distinct tissue sections. **E** Hydroxyproline content in liver tissues was determined. **F** The size of the granuloma area in *S. japonicum*-infected mice (AAV-EGFP + PBS + infected, AAV-MCM7 + PBS + infected, AAV-MCM7 + rhIL11 + infected) was measured and calculated. **G**, **H** qRT-PCR (**G**) was used to assess the expression levels of *Col1a1*, *α-Sma*, *Timp1*, and *Mmp2*, while Western blot (**H**) analysis focused on COL1A1 and α-SMA in liver tissues from the indicated groups, with the graph displaying protein levels. Data are presented as mean ± SD of 3–6 mice per group and are representative of three independent experiments. Statistical analyses were performed using unpaired Student’s *t*-test or one-way ANOVA. *P < 0.05; **P < 0.01; ***P < 0.001; ****P < 0.0001.

Given that hepatic MCM7 activates *IL11* transcription and IL11 promotes HSC activation [32], we hypothesized that MCM7 accelerates liver fibrosis by inducing IL11 release, which subsequently activates HSCs. To validate this hypothesis, primary HSCs isolated from uninfected mice were treated with conditioned medium from MCM7-overexpressing hepatocytes isolated from *S. japonicum*-infected mice, in the presence or absence of an IL11 neutralizing antibody. (Fig. 8A). MCM7 overexpression significantly upregulated the expression of fibrogenic markers, such as α-SMA and COL1A1, in HSCs. The IL11 neutralizing antibody inhibited the MCM7 overexpression-induced upregulation of α-SMA and COL1A1 in HSCs (Fig. 8A). Similar results were observed in co-culture of LX-2 cells with MCM7-overexpressing HepG2 cells (Fig. 8B). These results demonstrate that MCM7 induces IL11 secretion from hepatocytes, thereby promoting HSC activation in vitro.

Previous studies have shown that rhIL11 inhibits endogenous mouse IL11 activity in mice [33–35]. To explore whether MCM7 promotes liver fibrosis through IL11 in vivo, we administered rhIL11 to inhibit endogenous IL11 activity in mouse models subjected to *S. japonicum* infection or chronic CCl4 treatment. The efficacy of rhIL11 in suppressing the endogenous IL11 signaling pathway was confirmed by Western blot analysis in mice, consistent with previous studies (Fig. S16A, B) [33]. Our results demonstrated that rhIL11 treatment significantly alleviated histological injury, collagen deposition, liver fibrosis severity, and hydroxyproline content induced by MCM7 overexpression (Figs. 8C–F and S17A–C). Additionally, rhIL11 treatment attenuated the elevated mRNA and protein levels of profibrotic genes in mice with hepatocyte-specific MCM7 overexpression (Figs. 8G, H and S17D, E). In conclusion, our findings demonstrate that MCM7 enhances hepatocyte-derived IL11 production, which drives HSC activation and promotes liver fibrosis progression.

## Discussion

This study initially demonstrates that dysregulated MCM7-mediated protein interactions serve as critical regulatory factors in liver fibrosis development. Hepatocyte MCM7 facilitates the interaction between RACGAP1 and STAT3 by binding to SHCBP1, enhancing STAT3 phosphorylation and nuclear translocation, thereby activating the STAT3 signaling pathway. This activation leads to transcriptional upregulation of *IL11*, which further promotes HSC activation and ultimately drives liver fibrosis progression.

MCM7, a crucial component of the MCM complex, is primarily studied for its role in initiating DNA replication [11]. Some researchers have indicated that MCM7 not only has replication functions but also plays additional biological roles [12–17]. Notably, MCM7 has been reported to positively regulate the mRNA splicing of EGFR and PDGFR through its interaction with SF3B3 in prostate cancer, suggesting its significant role in the pathogenesis of liver fibrosis [19]. Previous studies have indicated that MCM7 is upregulated in HCC and regulated by miRNA-214 and arsenic trioxide (ATO), potentially affecting HCC progression via the PI3K/AKT pathway [18, 36, 37]. Notably, we observed that MCM7 expression was also increased in both liver cirrhosis patients and two murine liver fibrosis models induced by *S. japonicum* infection and CCl4 treatment, respectively. In the *S. japonicum*-infected murine model, fibrosis was characterized by granuloma formation and inflammatory infiltrates, while the CCl4-induced model showed widespread hepatocyte necrosis and bridging fibrosis [38, 39]. Despite these differences, HSC activation was a key driver of liver fibrosis in both models [40, 41]. Moreover, increased MCM7 expression was found to positively correlate with the severity of hepatic fibrosis in both *S. japonicum*- and CCl4-induced murine fibrosis models. Our further studies demonstrated that MCM7 is predominantly expressed in hepatocytes during liver fibrosis. MCM7 expression is known to be regulated by various intracellular signaling pathways, including the YAP/TAZ pathway in non-small cell lung cancer [14]. In this study, we found that YAP activates *MCM7* transcription in hepatocytes. The activation of the YAP/TAZ pathway in liver fibrosis is well-documented, which explains the increased MCM7 expression observed in liver fibrosis [25]. Moreover, MCM7 knockdown in hepatocytes via an AAV8 vector inhibited the development of liver fibrosis, while MCM7 overexpression exacerbated fibrosis induced by *S. japonicum* and CCl4. Collectively, these findings underscore the critical role of MCM7 in liver fibrosis and highlight its potential as a therapeutic target for treating this disease. While our observations in human tissue samples provide important context, the primary conclusions about MCM7’s role in liver fibrosis and its therapeutic potential are derived from extensive murine model studies. These models enabled us to explore underlying mechanisms and validate therapeutic strategies in a controlled experimental setting.

SHCBP1 has been reported to be closely associated with multiple signaling pathways, including TGF-β1/Smad, NF-κB, STAT3, MAPK/ERK, PI3K/Akt, and Wnt/β-catenin activation, which are involved in cell growth, proliferation, differentiation, tumorigenesis, and progression [28, 42]. Mechanistically, SHCBP1 has been identified as interacting with several proteins to perform a series of physiological or pathological functions [43–45]. It has been shown that under EGF stimulation, SHCBP1 interacts with β-catenin, promoting its nuclear translocation to activate β-catenin signaling [43]. In addition, SHCBP1 can bind to PLK1 to regulate cell mitosis [44]. Furthermore, the interaction between SHCBP1 and KIF23 plays functional roles in modulating the cell cycle and cisplatin resistance in head and neck squamous cell carcinoma [45]. Here, we provide the first evidence of a physical interaction between MCM7 and SHCBP1 in hepatocytes and identify the site where the N-terminal and AAA domains of MCM7 bind to multiple truncations of SHCBP1. These findings suggest that MCM7 and SHCBP1 interact via multiple binding interfaces, possibly attributed to redundant binding sites or flexible interaction modes within both proteins. RNA-seq analysis of MCM7 or SHCBP1 knockdown cells showed significant enrichment of downregulated genes in cytokine-cytokine receptor interactions, with IL11 being the most downregulated. Previous studies have demonstrated that IL11 released by hepatocytes is a critical factor in the progression of liver fibrosis [6, 30]. Importantly, both in vivo and in vitro studies demonstrated that overexpression of MCM7 or SHCBP1 increased IL11 expression, while their knockdown significantly reduced IL11 expression. Moreover, luciferase assays revealed that MCM7 activates *IL11* transcription through its interaction with SHCBP1. EGF stimulation increased the interaction between SHCBP1 and RACGAP1, which in turn attenuated the catalytic activity of RACGAP1 toward GTP-RAC1 in bladder cancer [46]. Inhibition of RACGAP1 was found to enhance STAT3 signaling, while RACGAP1 activity itself did not drive STAT3 nuclear localization in HEK293 SIE-Luc cells [47]. Interestingly, other studies have reported that RACGAP1 stimulates STAT3 phosphorylation and promotes its nuclear translocation, thereby affecting the expression of target genes, which facilitates cell proliferation, migration, and decreases chemosensitivity to doxorubicin [48]. In this study, we identified an interaction between SHCBP1 and RACGAP1 via mass spectrometry and co-immunoprecipitation. Furthermore, we demonstrated that SHCBP1 facilitates the binding of RACGAP1 to STAT3 and regulates STAT3 nuclear import. Moreover, we observed that MCM7 knockdown impaired the interaction between SHCBP1 and RACGAP1, as well as that between RACGAP1 and STAT3. Furthermore, overexpression of RACGAP1 or SHCBP1 rescued the reduced STAT3 phosphorylation levels caused by MCM7 knockdown. It has been reported that IL11 activates the STAT3 pathway [49]. Interestingly, *IL11* has been identified as a direct transcriptional target of STAT3 [50]. Our findings reveal that MCM7 promotes STAT3 binding to the *IL11* promoter, specifically within the −1348 to −1337 region. Additionally, ChIP assays revealed that SHCBP1 and MCM7 do not directly bind to the *IL11* promoter. Collectively, these findings emphasize the critical role of MCM7 in activating *IL11* transcription via the SHCBP1-RACGAP1-STAT3 axis. The involvement of MCM7 in this regulatory pathway unveils a novel mechanism by which MCM7 modulates IL11 expression, a process that may critically influence liver fibrosis progression and potentially other pathological conditions.

IL11 is a cytokine in the IL-6 family that participates in regulating signaling pathways such as STAT3, TGF-β, ERK, and MAPK [51, 52]. Numerous studies have established IL11 as a pivotal mediator of organ fibrosis and dysfunction [52]. IL11 levels correlate with liver fibrosis severity and promote HSC activation [30]. Inhibiting IL11 signaling reduces hepatocyte death, liver fibrosis, inflammation, and steatosis in murine models of non-alcoholic steatohepatitis [28]. Interestingly, rhIL11 acts as an inhibitor of mouse IL11 activity and has been administered to patients in clinical trials for liver diseases [33–35]. Our study identified a significant positive correlation between MCM7 and IL11 expression in patients with liver cirrhosis. In vitro studies have shown that an IL11 neutralizing antibody significantly suppresses HSC activation triggered by MCM7-overexpressing hepatocytes. These findings suggest that MCM7 promotes the release of IL11 from hepatocytes, thereby activating HSCs. Furthermore, in vivo experiments revealed that treatment with rhIL11 in MCM7-overexpressing mice substantially suppressed the progression of liver fibrosis. This indicates that inhibiting IL11 signaling can mitigate the fibrosis exacerbated by MCM7 overexpression. Based on these results, small-molecule inhibitors that selectively targeting MCM7 or the SHCBP1-RACGAP1-STAT3 axis could disrupt their respective pro-fibrotic signaling cascades. Given that inhibiting IL11 signaling reduces liver fibrosis and multiple pathways regulate IL11 expression, combining MCM7 or SHCBP1-RACGAP1-STAT3 axis inhibitors with existing IL11 inhibitors may achieve synergistic multi-target action, significantly attenuating or even reversing liver fibrosis progression.

In conclusion, our study underscores the pivotal role of MCM7 in liver fibrosis. We present compelling evidence that MCM7 promotes IL11 expression via the SHCBP1-RACGAP1-STAT3 signaling axis, thereby driving HSC activation and fibrosis progression. These findings establish MCM7 as a critical regulatory node in liver fibrogenesis, unveiling a novel mechanism within the fibrotic regulatory network and highlighting its potential as a therapeutic target for developing innovative antifibrotic strategies.

## Supplementary information

Supplementary Figure and Table

original western blots

## Data Availability

The RNA-seq FASTQ files were deposited in Gene Expression Omnibus (GEO) (https://www.ncbi.nlm.nih.gov/geo/query/acc.cgi?acc=GSE275059), accessible password is mhwjeoosljobvob; (https://www.ncbi.nlm.nih.gov/geo/query/acc.cgi?acc=GSE276001), accessible password is gzcrqoqqrperbqf. The mass spectrometry proteomics data have been deposited to the ProteomeXchange Consortium via the PRIDE partner repository with the dataset identifier PXD055175. Reviewer account details: Username is reviewer_pxd055175@ebi.ac.uk, Password is e7q2xc1Xbz2D.The two in-house RNA-seq datasets generated by our research group has not been published yet. Published RNA-seq datasets were obtained from GEO (GSE61376, GSE25713, GSE55747).
