# Supplementary material for: MCM7 promotes liver fibrosis by transcriptionally regulating IL11 via the SHCBP1-RACGAP1-STAT3 axis
Source: Cell Death Dis. 2025 Aug 11;16(1):608. doi: 10.1038/s41419-025-07937-x (PMC12339988; doi:10.1038/s41419-025-07937-x)
Supplement: Supplementary file 1 — Supplementary Figure and Table [file 41419_2025_7937_MOESM1_ESM.pdf]

Supplementary figures and figure legends

Supplementary figure 1

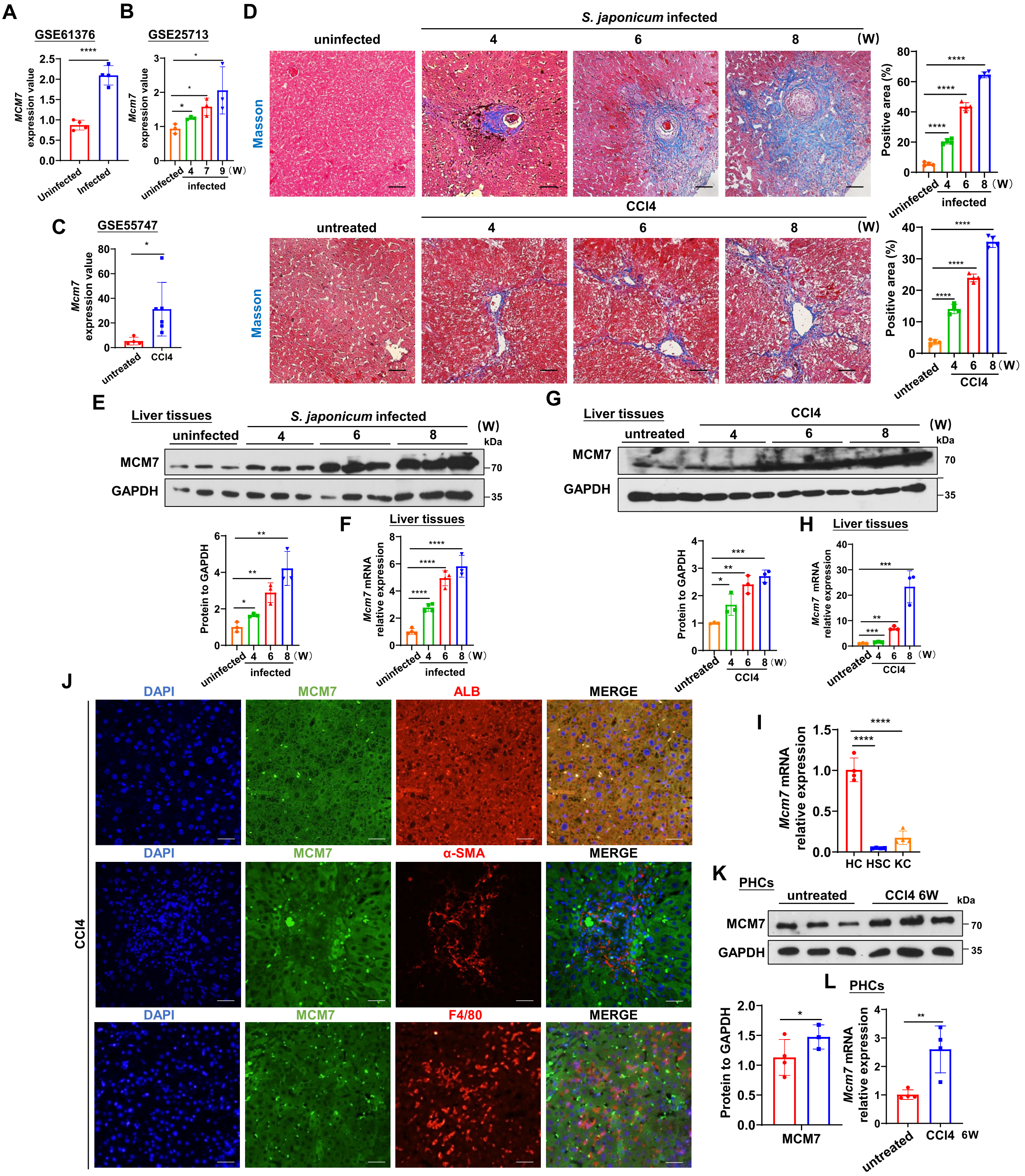

Figure S1. Upregulation of hepatic MCM7 expression in livers fibrosis. (A) Analysis of *MCM7* transcript levels in human liver samples from uninfected individuals (n = 4) and *S. japonicum*-infected patients (n = 4), obtained from the GEO database (GSE61376). (B) Analysis of *Mcm7* transcript levels in mouse liver samples from uninfected controls (n = 3) and groups infected with *S. japonicum* for 3 weeks (n = 3), 4 weeks (n = 3), 7 weeks (n = 3), and 9 weeks (n = 3), obtained from the GEO database (GSE25713). (C) Analysis of *Mcm7* transcript levels in mouse liver samples from healthy controls (n = 4) and groups with CCl4-induced liver fibrosis (n = 6), obtained from the GEO database (GSE55747). (D) Masson's trichrome staining of liver tissues from *S. japonicum*-infected (top) or CCl4-treated (bottom) mice (4, 6, and 8 weeks) assessed fibrosis (scale bar: 50 μm), with quantification of positive areas shown in the corresponding graphs. (E-H) MCM7 expression levels were assessed in liver tissues from *S. japonicum*-infected or CCl4-treated mice at 4, 6, and 8 weeks by Western blot (E, G) for protein expression, with the graph displaying protein levels, and qRT-PCR (F, H) for mRNA expression. (I) *Mcm7* transcript levels were analyzed in isolated HCs, HSCs, and KCs from the livers of normal mice. (J) Immunofluorescence (IF) analysis was performed to assess the colocalization of MCM7 in fibrotic liver sections from mice treated with CCl4 for 6 weeks, with ALB, α-SMA and F4/80 used to mark HCs, HSCs, and KCs, respectively. Nuclei were stained with DAPI (scale bar: 50 μm). (K, L) MCM7 expression levels were assessed in primary hepatocytes (PHCs) from untreated and CCl4-treated mice at 6 weeks by Western blot (K) for protein expression, with the graph displaying protein levels, and qRT-PCR (L) for mRNA expression. Data are expressed as the mean ± SD of 3–6 mice per group and are representative of three independent experiments. Statistical analyses were performed using unpaired Student's t test or one-way ANOVA. $^{*}P < 0.05$; $^{**}P < 0.01$; $^{***}P < 0.001$; $^{****}P < 0.0001$.

Supplementary figure 2

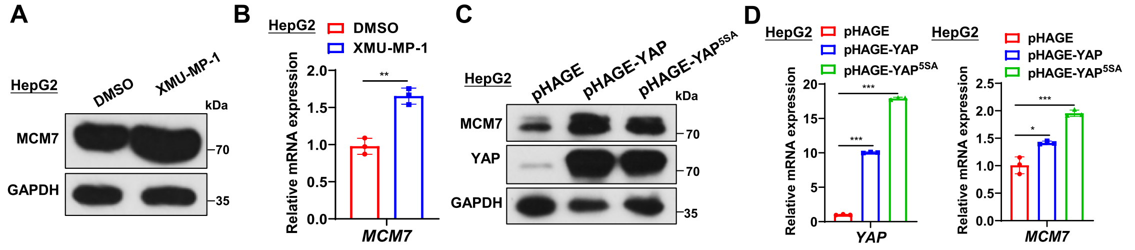

Figure S2. The increased expression of MCM7 is driven by YAP activation. (A, B) Detection of protein (A) and mRNA (B) levels of MCM7 in HepG2 cells after XMU-MP-1 stimulation for 24 h. (C, D) Detection of protein (C) and mRNA (D) levels of YAP and MCM7 in HepG2 cells transfected with pHAGE-YAP or pHAGE-YAP^{5SA}. Data are presented as mean ± SD of three independent experiments. Statistical analyses were performed using an unpaired Student's t-test or one-way ANOVA. *$P < 0.05$; **$P < 0.01$; ***$P < 0.001$.

Supplementary figure 3

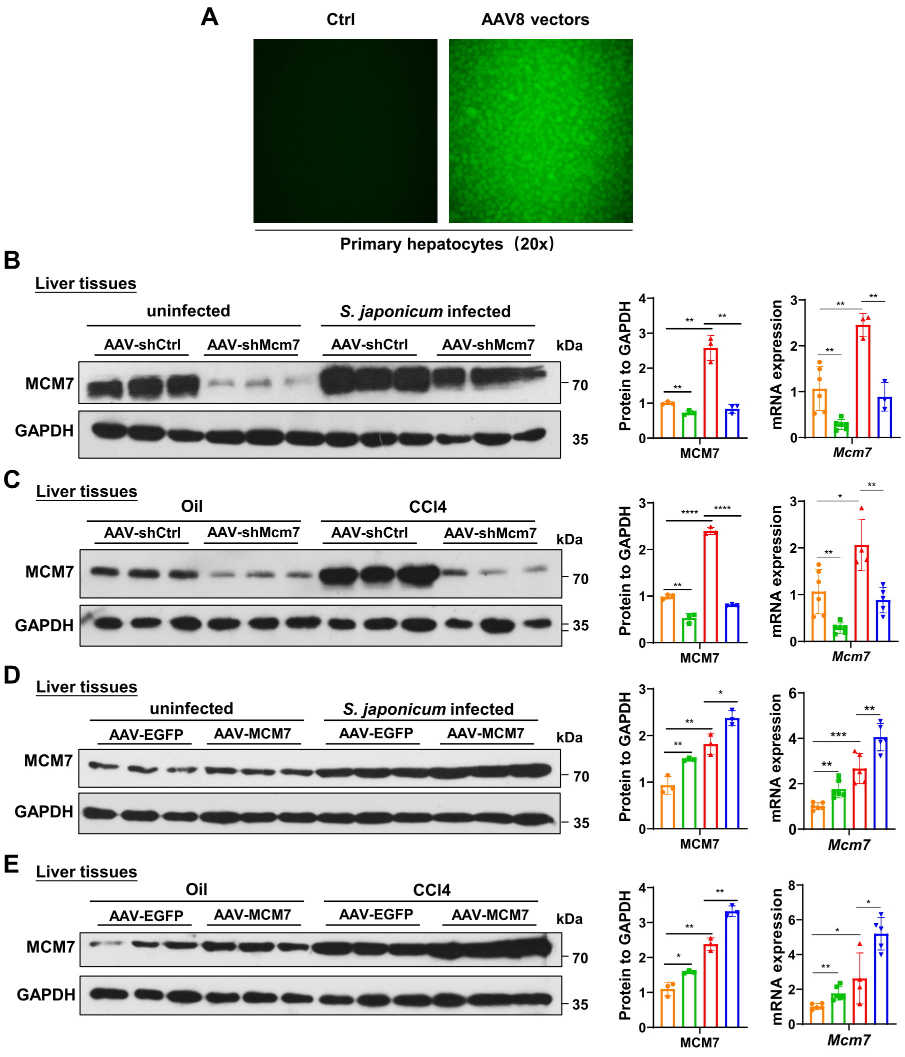

Figure S3. The expression of MCM7 in *S. japonicum*-infected or CCl4-induced mice after AAV-shMcm7 or AAV-MCM7 injection. (A) Representative fluorescence microscopy image of GFP signal (green) in primary hepatocytes isolated from AAV-injected and control mice, observed with a 20 x objective lens. (B–E) qRT-PCR (right) and Western blot (left) analyses, along with protein level quantification, were performed to assess MCM7 expression in liver tissues of mice with *S. japonicum*- or CCl4-induced liver fibrosis, following MCM7 knockdown or overexpression mediated by AAV8 vectors. Data are expressed as the mean ± SD for 3–6 mice per group and are representative of three independent experiments. Statistical analyses were performed using an unpaired Student's t-test or one-way ANOVA. * $P< 0.05$, ** $P < 0.01$, *** $P < 0.001$, **** $P < 0.0001$.

Supplementary figure 4

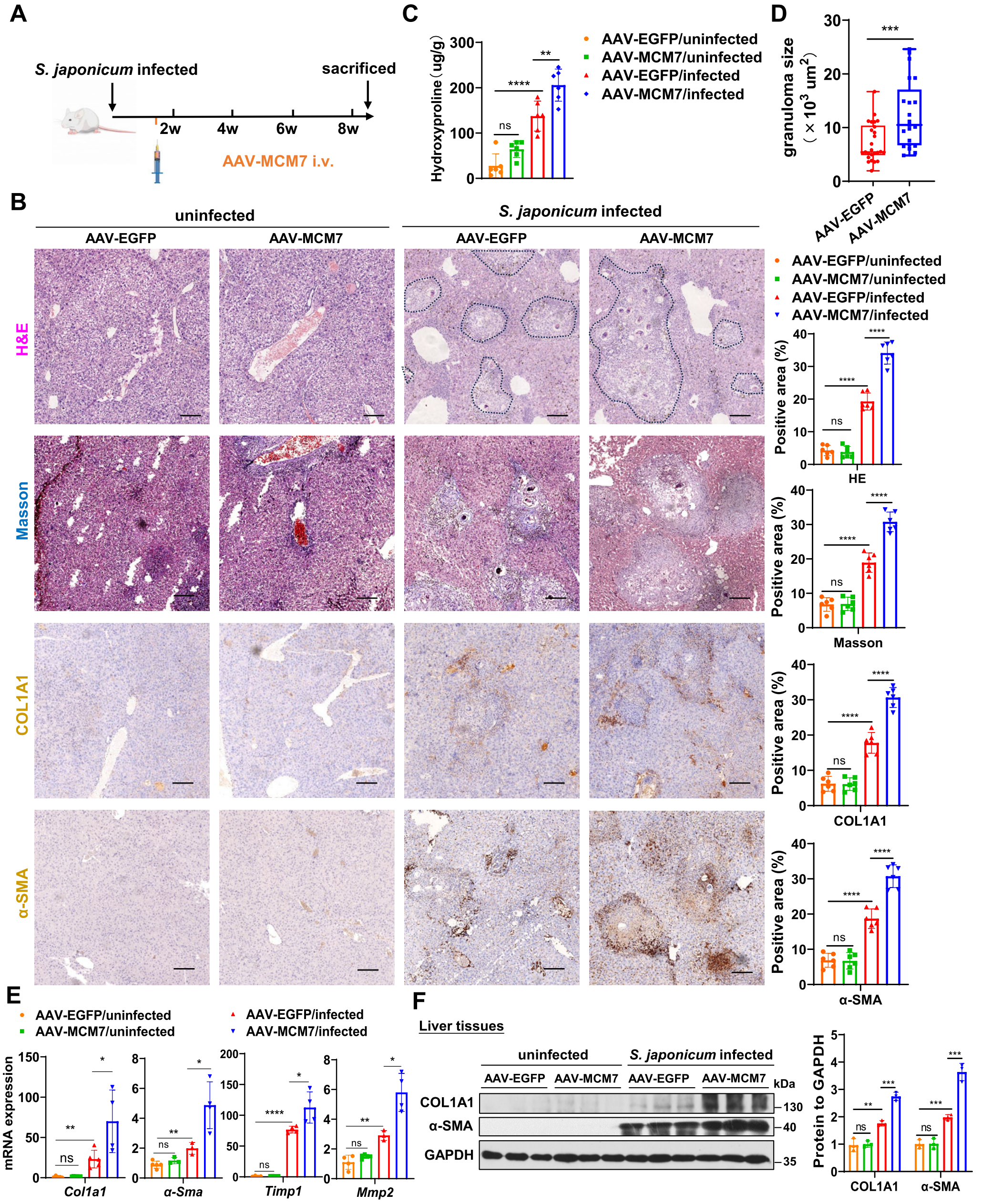

Figure S4. Hepatocyte MCM7 overexpression exacerbates liver fibrosis in *S. japonicum*-induced mice. (A) Experimental design schematic: mice were infected with *S. japonicum* and received intravenous injections of either AAV-EGFP or AAV-MCM7 on day 10 post-infection. Liver samples were collected at 8 weeks post-infection for analysis. (B) H&E staining (areas positive for liver fibrosis are delineated by black dashed lines), Masson's trichrome staining, COL1A1 staining, and α-SMA staining (all scale bars: 100 μm) of liver sections from the indicated groups (AAV-EGFP/uninfected, AAV-MCM7/uninfected, AAV-EGFP/infected, AAV-MCM7/infected). Graphs show the quantified positive areas for each stain, determined using ImageJ software from multiple randomly selected fields across distinct tissue sections. (C) Hydroxyproline content in liver tissues was determined. (D) The size of the granuloma area in *S. japonicum*-induced mice (AAV-EGFP/infected, AAV-MCM7/infected) was measured and calculated. (E, F) qRT-PCR (E) was used to assess the expression levels of *Col1a1, α-Sma, Timp1, and Mmp2*, while Western blot (F) analysis focused on COL1A1 and α-SMA in liver tissues from the indicated groups, with the graph displaying protein levels. Data are presented as the mean ± SD of 3–6 mice per group and are representative of three independent experiments. Statistical analyses were performed using an unpaired Student's t-test or one-way ANOVA. *P < 0.05; **P < 0.01; ***P < 0.001; ****P < 0.0001; ns, not significant.

Supplementary figure 5

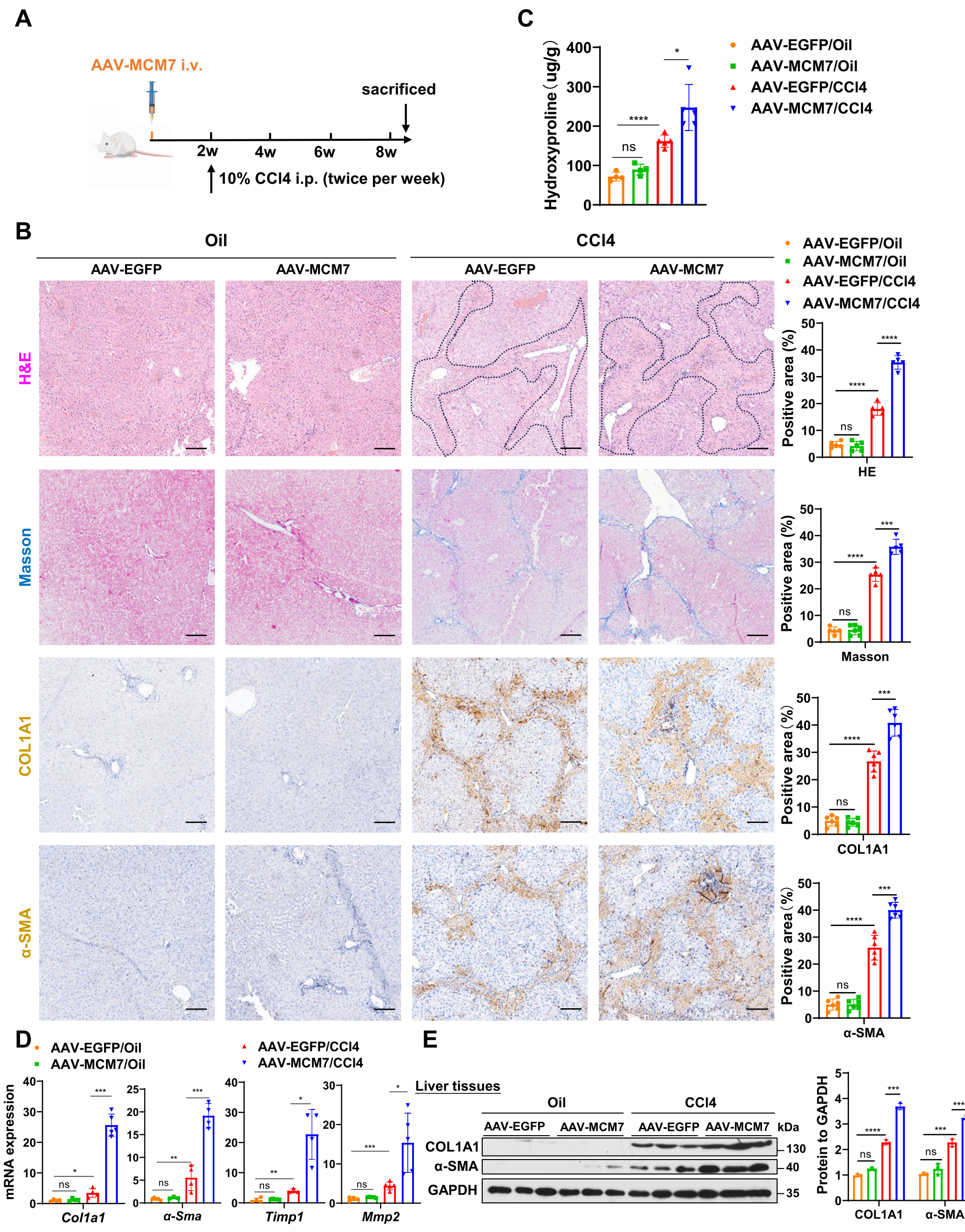

Figure S5. Hepatocyte MCM7 overexpression exacerbates liver fibrosis in CCl4-treated mice. (A) Experimental design schematic: mice were intravenously injected with AAV-EGFP or AAV-MCM7. Two weeks after the AAV injection, mice began receiving intraperitoneal injections of 10% CCl4 twice per week. Liver samples were collected at 6 weeks post-CCl4 treatment for analysis. (B) H&E staining (areas positive for liver fibrosis are delineated by black dashed lines), Masson's trichrome staining, COL1A1 staining, and α-SMA staining (all scale bars: 100 μm) of liver sections from the indicated groups (AAV-EGFP/Oil, AAV-MCM7/Oil, AAV-EGFP/CCl4, AAV-MCM7/CCl4). Graphs show the quantified positive areas for each stain, determined using ImageJ software from multiple randomly selected fields across distinct tissue sections. (C) Hydroxyproline content in liver tissues was determined. (D, E) qRT-PCR (D) assessed the expression levels of *Col1a1*, *α-Sma*, *Timp1*, and *Mmp2*, while Western blot (E) analysis focused on COL1A1 and α-SMA in liver tissues from the indicated groups, with the graph displaying protein levels. Data are presented as the mean ± SD for 3–6 mice per group and are representative of three independent experiments. Statistical analyses were performed using an unpaired Student's t-test or one-way ANOVA. $^{*}P < 0.05$; $^{**}P < 0.01$; $^{***}P < 0.001$; $^{****}P < 0.0001$; ns, not significant.

Supplementary figure 6

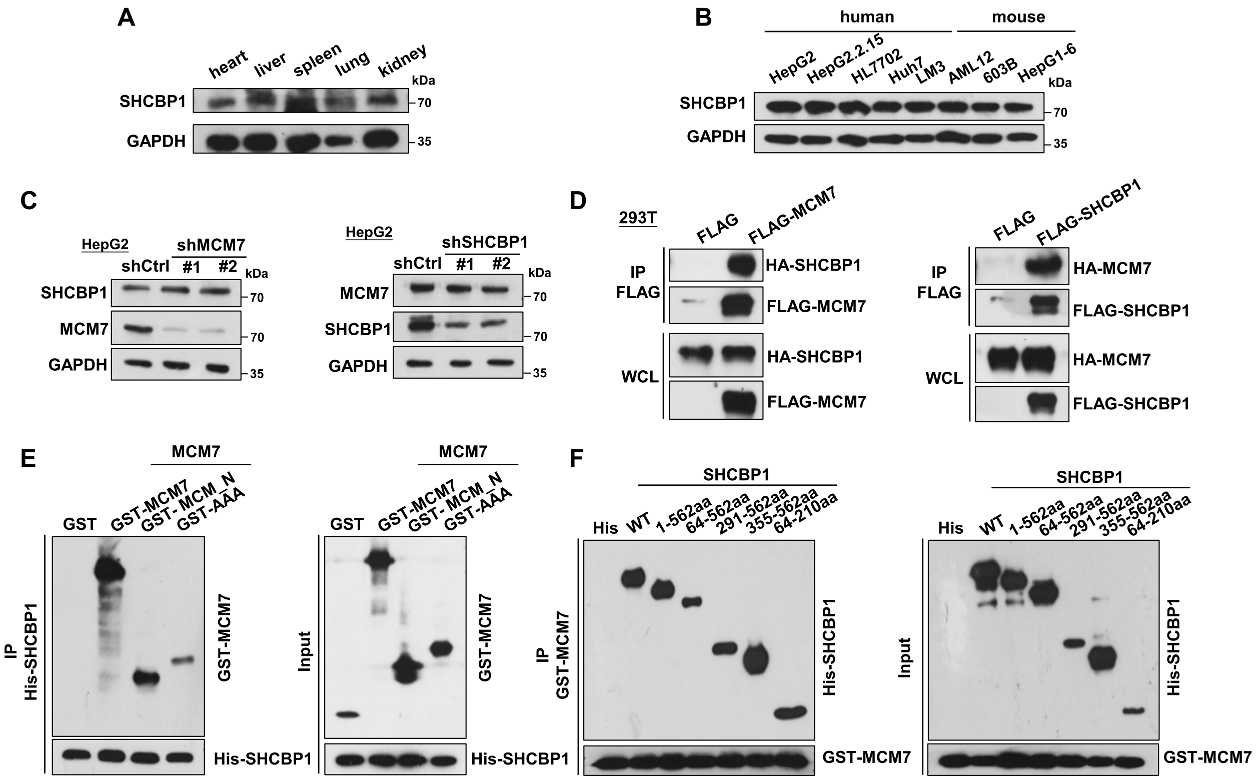

Figure S6. Expression and interaction analysis of SHCBP1. (A, B) Western blot analysis of SHCBP1 expression levels in various mouse tissues (A) and in different human and mouse cell lines (B). (C) Western blot analysis showing SHCBP1 expression after MCM7 knockdown (left) and MCM7 expression after SHCBP1 knockdown (right) in HepG2 cells. (D) Exogenous interaction between FLAG/HA-tagged MCM7 and FLAG/HA-tagged SHCBP1 was validated by Co-IP in HEK293T cells. (E, F) GST/His pull-down assay to examine the interaction between His-SHCBP1 and various GST-MCM7 truncations (E) or GST-MCM7 and various His-tagged SHCBP1 truncations (F) in an *in vitro* system. Input and immunoprecipitation (IP) samples were analyzed by Western blot.

Supplementary figure 7

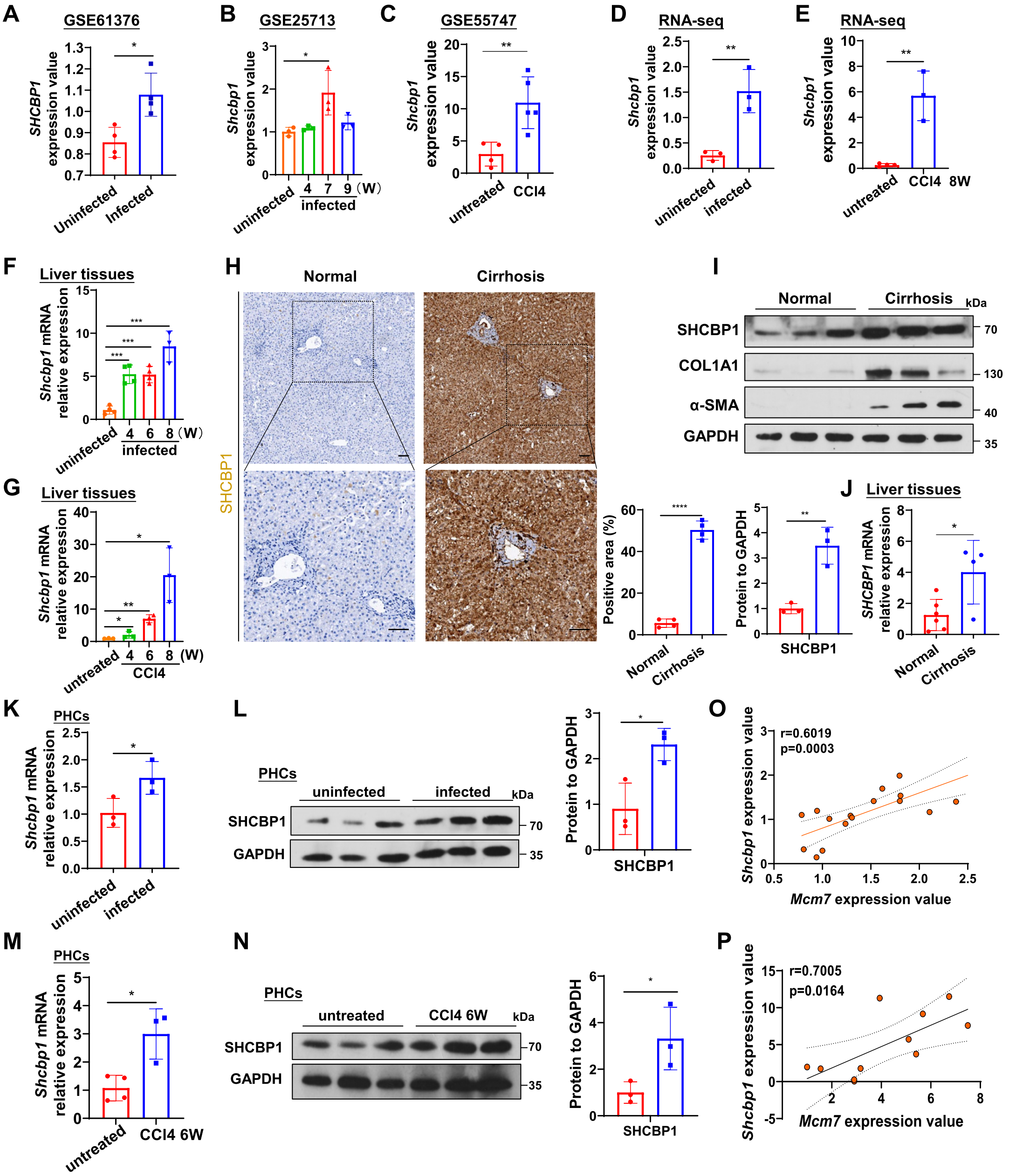

Figure S7. Upregulation of hepatic SHCBP1 expression in liver fibrosis. (A) Analysis of *SHCBP1* transcript levels in human liver samples from uninfected individuals (n = 4) and *S. japonicum*-infected patients (n = 4), obtained from the GEO database (GSE61376). (B) Analysis of *Shcbp1* transcript levels in mouse liver samples from uninfected controls (n = 3) and groups infected with *S. japonicum* for 3 weeks (n = 3), 4 weeks (n = 3), 7 weeks (n = 3), and 9 weeks (n = 3), obtained from the GEO database (GSE25713). (C) Analysis of *Shcbp1* transcript levels in mouse liver samples from healthy controls (n = 4) and groups with CCl4-induced liver fibrosis (n = 6), obtained from the GEO database (GSE55747). (D, E) *Shcbp1* transcript levels were assessed using two in-house RNA-seq data obtained from fibrotic mouse liver tissues following 8-week *S. japonicum* infection (D) or 8-week CCl4 treatment (E). (F, G) *Shcbp1* mRNA expression levels were assessed in fibrotic liver tissues from mice infected with *S. japonicum* (F) or treated with CCl4 (G) for 4, 6, and 8 weeks. (H–J) SHCBP1 expression levels in human liver tissue samples, including normal (n = 3) and cirrhosis (n = 3) groups, were assessed by IHC (H) (scale bar: 100 μm), Western blot (I) for protein levels, and qRT-PCR (J) for mRNA expression, with corresponding graphs showing SHCBP1^{+} staining, protein expression, and mRNA levels, respectively. (K, L) SHCBP1 mRNA expression (K) and protein expression (L) were assessed in primary hepatocytes (PHCs) isolated from uninfected and *S. japonicum*-infected mice, with the graph displaying protein levels. (M, N) SHCBP1 expression levels were assessed in primary hepatocytes (PHCs) from untreated and CCl4-treated mice by qRT-PCR (M) and Western blot (N), with the graph displaying protein levels. (O) Correlation analysis between *Mcm7* and *Shcbp1* mRNA expression levels in liver tissues from *S. japonicum*-infected and uninfected samples based on GEO and RNA-seq data. (P) Correlation analysis between *Mcm7* and *Shcbp1* mRNA expression levels in liver tissues from untreated and CCl4-treated samples based on GEO and RNA-seq data. Data are expressed as the mean ± SD of 3–6 mice per group and are representative of three independent experiments. Statistical analyses were performed using unpaired Student's t-test and one-way ANOVA. *P < 0.05; **P < 0.01; ***P < 0.001; ****P < 0.0001.

Supplementary figure 8

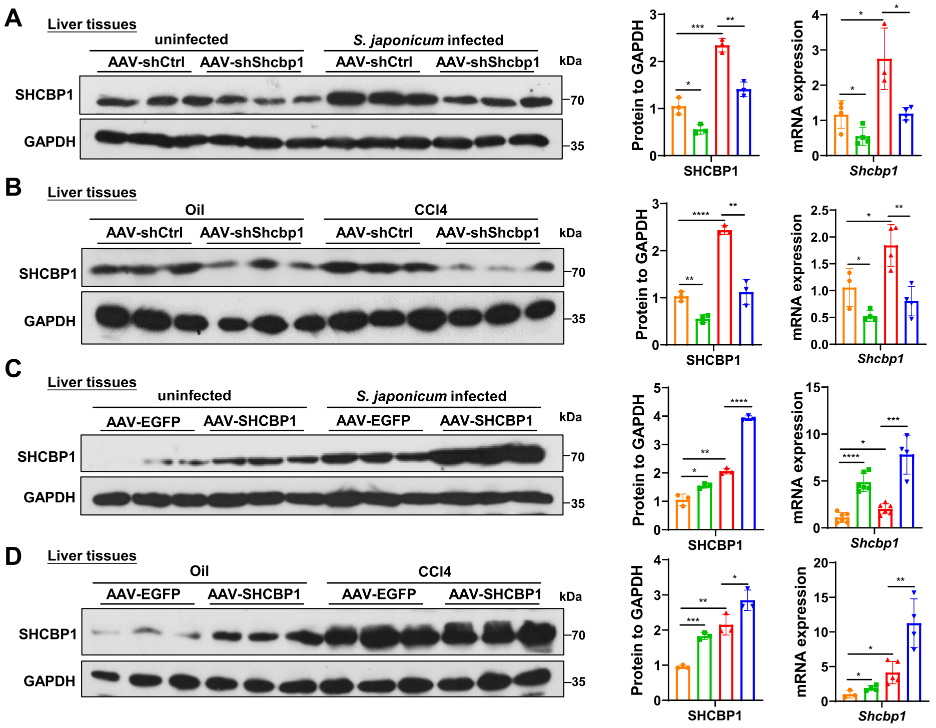

Figure S8. The expression of SHCBP1 in *S. japonicum*-infected or CCl4-induced mice after AAV-shShcbp1 or AAV-SHCBP1 injection. (A–D) qRT-PCR (right) and Western blot (left) analyses, along with protein level quantification, were performed to assess SHCBP1 expression in liver tissues of mice with *S. japonicum*- or CCl4-induced liver fibrosis, following SHCBP1 knockdown or overexpression mediated by AAV8 vectors. Data are expressed as the mean ± SD of 3–6 mice per group and are representative of three independent experiments. Statistical analyses were performed using an unpaired Student's t-test or one-way ANOVA. *P < 0.05, **P < 0.01, ***P < 0.001, ****P < 0.0001.

Supplementary figure 9

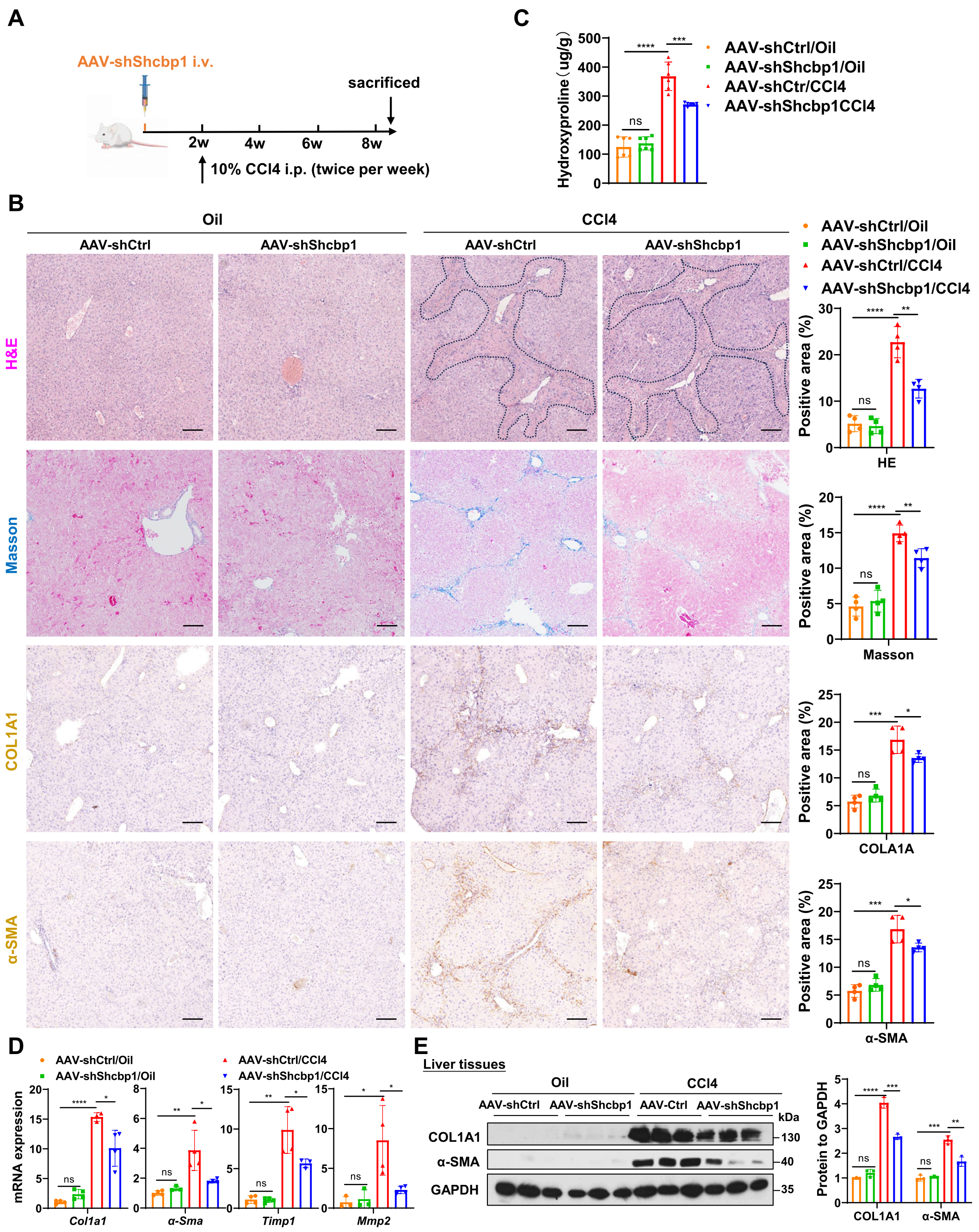

Figure S9. Hepatocyte SHCBP1 knockdown attenuates liver fibrosis in CCl4-induced mice. (A) Experimental design schematic: mice were intravenously injected with AAV-shCtrl or AAV-shShcbp1. Two weeks after the AAV injection, mice began receiving intraperitoneal injections of 10% CCl4 twice per week. Liver samples were collected at 6 weeks post-CCl4 treatment for analysis. (B) H&E staining (areas positive for liver fibrosis are delineated by black dashed lines), Masson's trichrome staining, COL1A1 staining, and α-SMA staining (all scale bars: 100 µm) of liver sections from the indicated groups (AAV-shCtrl/Oil, shShcbp1 /Oil, AAV-shCtrl/CCl4, AAV-shShcbp1/CCl4). Graphs show the quantified positive areas for each stain, determined using ImageJ software from multiple randomly selected fields across distinct tissue sections. (C) Hydroxyproline content in liver tissues was determined. (D, E) qRT-PCR (D) assessed the expression levels of *Col1a1*, *α-Sma*, *Timp1*, and *Mmp2*, while Western blot (E) analysis focused on COL1A1 and α-SMA in liver tissues from the indicated groups, with the graph displaying protein levels. Data are presented as the mean ± SD of 3–6 mice per group and are representative of three independent experiments. Statistical analyses were performed using an unpaired Student’s t-test or one-way ANOVA. *P < 0.05; **P < 0.01; ***P < 0.001; ****P < 0.0001; ns, not significant.

Supplementary figure 10

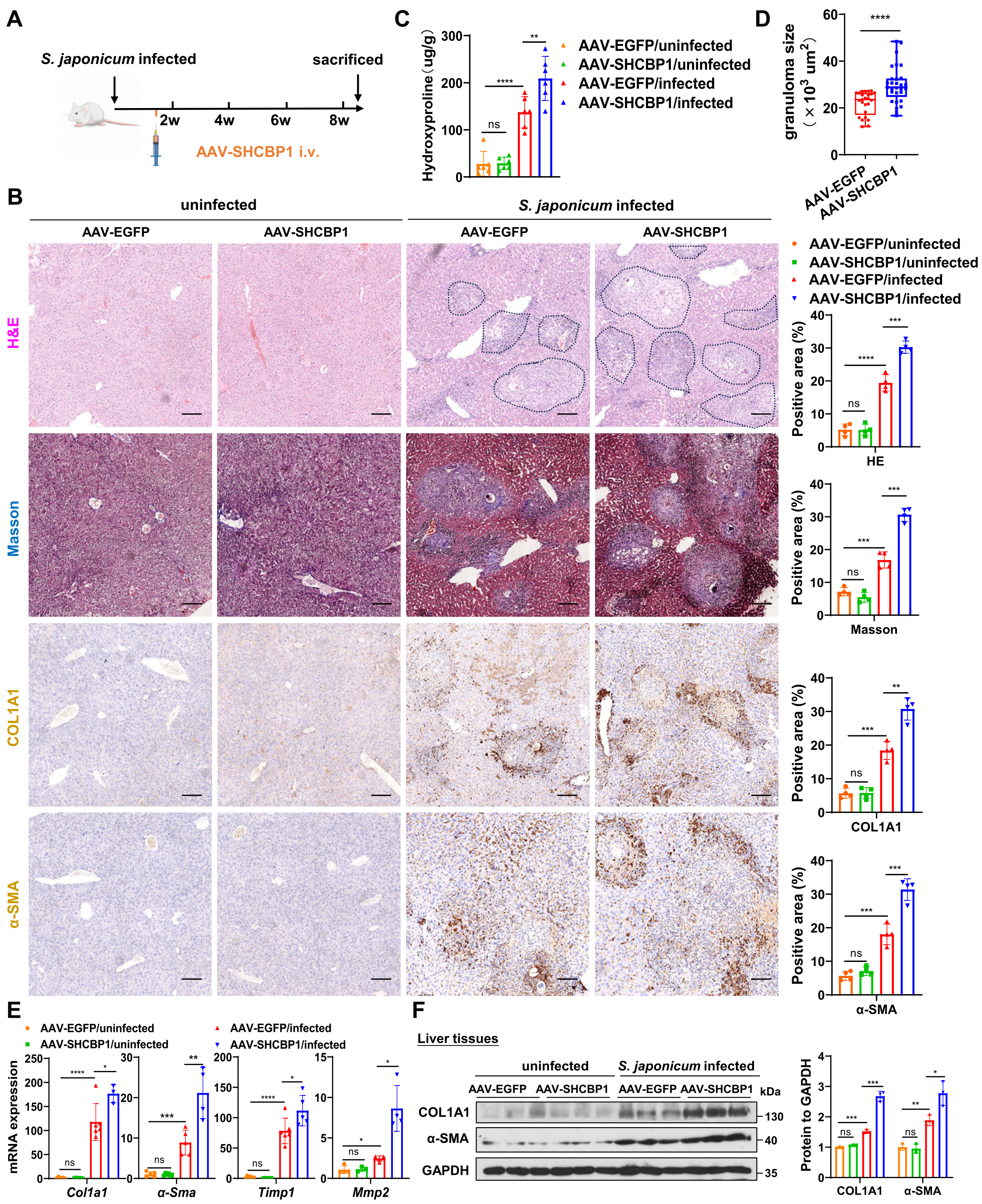

Figure S10. Hepatocyte SHCBP1 overexpression exacerbates liver fibrosis in *S. japonicum*-induced mice. (A) Experimental design schematic: mice were infected with *S. japonicum* and received intravenous injections of either AAV-EGFP or AAV-SHCBP1 on day 10 post-infection. Liver samples were collected at 8 weeks post-infection for analysis. (B) H&E staining (areas positive for liver fibrosis are delineated by black dashed lines), Masson's trichrome staining, COL1A1 staining, and α-SMA staining (all scale bars: 100 μm) of liver sections from the indicated groups (AAV-EGFP/uninfected, AAV-SHCBP1/uninfected, AAV-EGFP/infected, AAV-SHCBP1/infected). Graphs show the quantified positive areas for each stain, determined using ImageJ software from multiple randomly selected fields across distinct tissue sections. (C) Hydroxyproline content in liver tissues was determined. (D) The size of the granuloma area in *S. japonicum*-induced mice (AAV-EGFP/infected, AAV-SHCBP1/infected) was measured and calculated. (E, F) qRT-PCR (E) was used to assess the expression levels of *Col1a1*, *α-Sma*, *Timp1*, and *Mmp2*, while Western blot (F) analysis focused on COL1A1 and α-SMA in liver tissues from the indicated groups, with the graph displaying protein levels. Data are presented as the mean ± SD of 3–6 mice per group and are representative of three independent experiments. Statistical analyses were performed using an unpaired Student's t-test or one-way ANOVA. *P < 0.05; **P < 0.01; ***P < 0.001; ****P < 0.0001; ns, not significant.

Supplementary figure 11

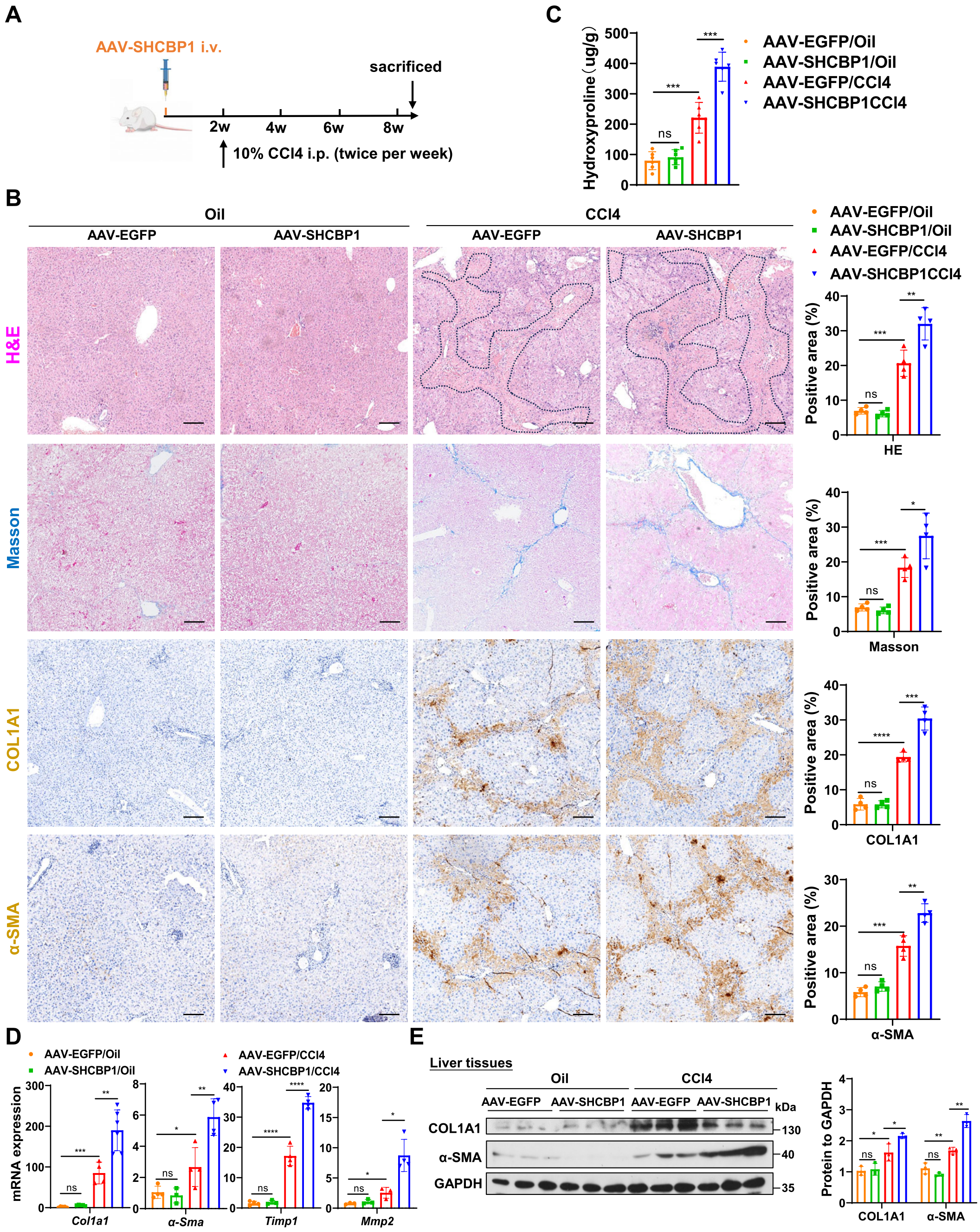

Figure S11. Hepatocyte SHCBP1 overexpression exacerbates liver fibrosis in CCl4-induced mice. (A) Experimental design schematic: mice were intravenously injected with AAV-EGFP or AAV-SHCBP1. Two weeks after the AAV injection, mice began receiving intraperitoneal injections of 10% CCl4 twice per week. Liver samples were collected at 6 weeks post-CCl4 treatment for analysis. (B) H&E staining (areas positive for liver fibrosis are delineated by black dashed lines), Masson's trichrome staining, COL1A1 staining, and α-SMA staining (all scale bars: 100 μm) of liver sections from the indicated groups (AAV-EGFP/Oil, AAV-SHCBP1/Oil, AAV-EGFP/CCl4, AAV-SHCBP1/CCl4). Graphs show the quantified positive areas for each stain, determined using ImageJ software from multiple randomly selected fields across distinct tissue sections. (C) Hydroxyproline content in liver tissues was determined. (D, E) qRT-PCR (D) assessed the expression levels of *Col1a1*, *α-Sma*, *Timp1*, and *Mmp2*, while Western blot (E) analysis focused on COL1A1 and α-SMA in liver tissues from the indicated groups, with the graph displaying protein levels. Data are presented as the mean ± SD for 3-6 mice per group and are representative of three independent experiments. Statistical analyses were performed using an unpaired Student's t-test or one-way ANOVA. *P < 0.05; **P < 0.01; ***P < 0.001; ****P < 0.0001; ns, not significant.

Supplementary figure 12

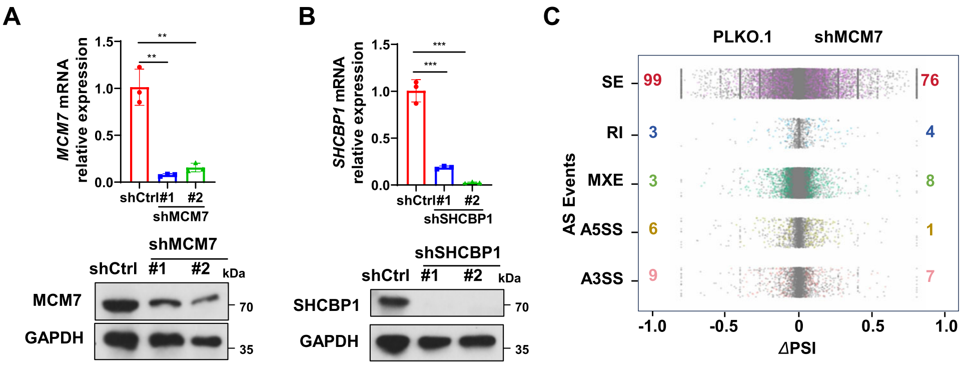

Figure S12. Analysis of alternative splicing events in MCM7 knockdown cells. (A) *MCM7* mRNA expression levels measured by qRT-PCR (top) and protein expression levels analyzed by Western blot (bottom) in hepatocytes stably expressing control shRNA (shCtrl) or MCM7 shRNA (shMCM7 #1 and #2). (B) *SHCBP1* mRNA expression levels measured by qRT-PCR (top) and protein expression levels analyzed by Western blot (bottom) in hepatocytes stably expressing control shRNA (shCtrl) or SHCBP1 shRNA (shSHCBP1 #1 and #2). (C) Scatter plots show changes in splicing events between shMCM7 and PLKO.1 hepatocytes. Using rMATS, five types of AS events were analyzed: RIs, skipped exons, A5SS, A3SS and mutually exclusive exons. Significantly changed events ($|\Delta PSI| > 0.05$, $FDR < 0.05$, and supporting reads ≥ 5) are shown by color dots. Data are presented as mean ± SD of three independent experiments. Statistical analyses were performed using unpaired Student's t-test. $^{**}P < 0.01$; $^{***}P < 0.001$.

Supplementary figure 13

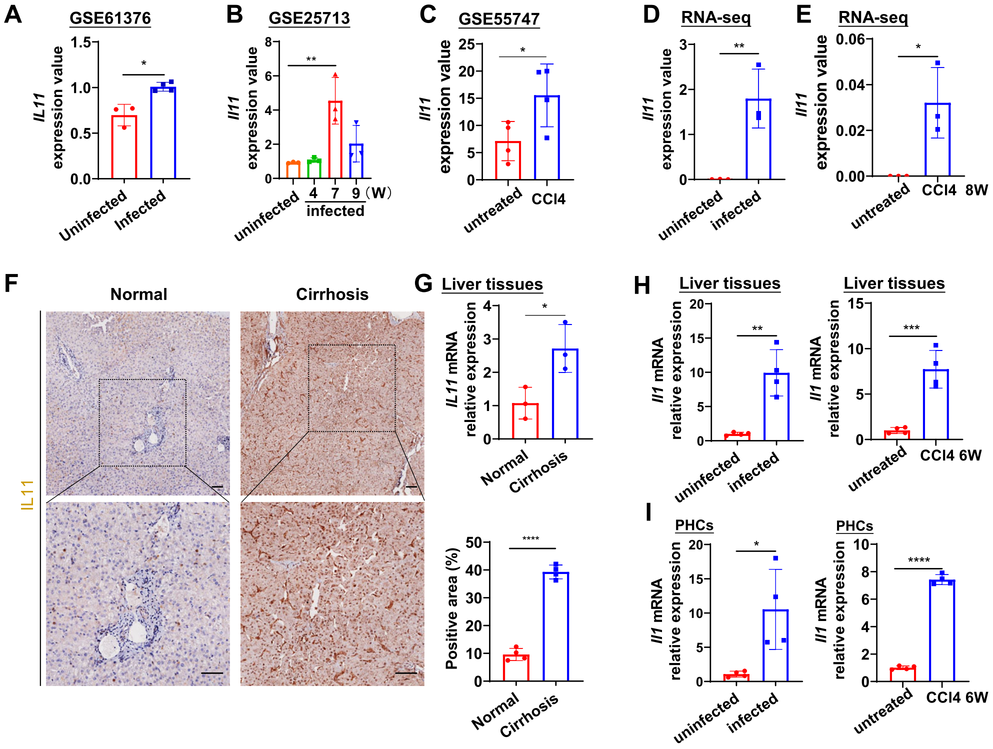

Figure S13. Upregulation of hepatic IL11 expression in liver fibrosis. (A) Analysis of *IL11* transcript levels in human liver samples from uninfected individuals (n = 4) and *S. japonicum*-infected patients (n = 4), obtained from the GEO database (GSE61376). (B) Analysis of *Il11* transcript levels in mouse liver samples from uninfected controls (n = 3) and groups infected with *S. japonicum* for 3 weeks (n = 3), 4 weeks (n = 3), 7 weeks (n = 3), and 9 weeks (n = 3), obtained from the GEO database (GSE25713). (C) Analysis of *Il11* transcript levels in mouse liver samples from healthy controls (n = 4) and groups with CCl4-induced liver fibrosis (n = 6), obtained from the GEO database (GSE55747). (D, E) *Il11* transcript levels were assessed using two in-house RNA-seq data obtained from fibrotic mouse liver tissues following 8-week *S. japonicum* infection (D) or 8-week CCl4 treatment (E). (F, G) IL11 expression levels in human liver tissue samples, including normal (n = 3) and cirrhosis (n = 3) groups, determined by immunohistochemistry (F) (scale bar: 50 μm), and qRT-PCR (G). (H) *Il11* mRNA expression levels in 8-week *S. japonicum*-infected (left) and 6-week CCl4-induced (right) mouse livers assessed by qRT-PCR. (I) *Il11* mRNA levels were assessed in primary hepatocytes (PHCs) isolated from 8-week *S. japonicum*-infected (left) and 6-week CCl4-induced (right) mice. Data are expressed as the mean ± SD of 3–6 mice per group and are representative of three independent experiments. Statistical analyses were performed using unpaired Student's t-test. *P < 0.05; **P < 0.01; ***P < 0.001; ****P < 0.0001.

Supplementary figure 14

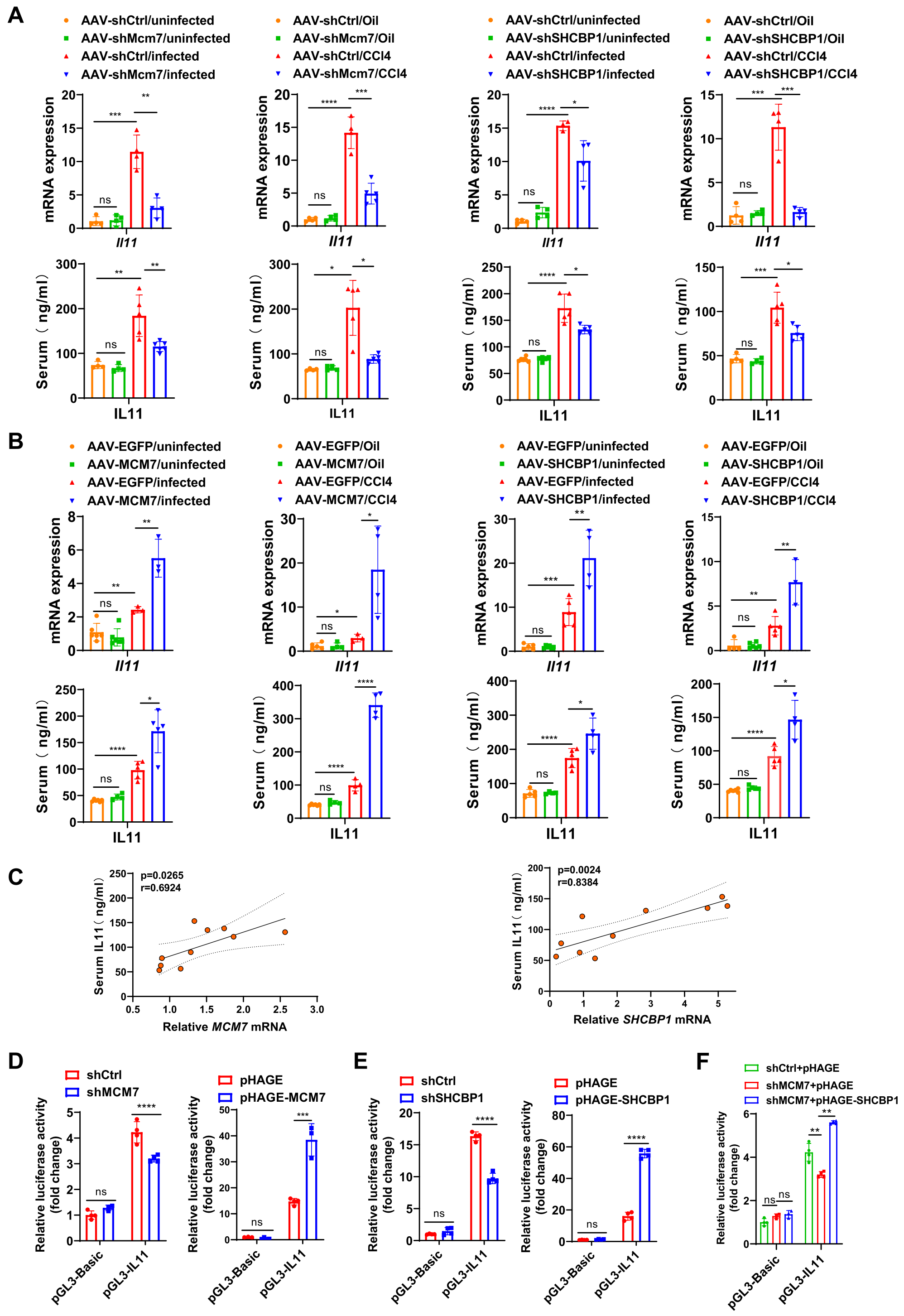

Figure S14. MCM7-mediated IL11 activation through SHCBP1. (A) IL11 mRNA levels measured by qRT-PCR (top) and protein levels in serum by ELISA (bottom) in *S. japonicum*- and CCl4-induced liver fibrosis mouse models with MCM7 or SHCBP1 knockdown. (B) IL11 mRNA levels measured by qRT-PCR (top) and protein levels in serum by ELISA (bottom) in *S. japonicum*- and CCl4-induced liver fibrosis mouse models with MCM7 or SHCBP1 overexpression. (C) Correlation between hepatic *MCM7* or *SHCBP1* mRNA expression and serum IL11 levels in cirrhotic patients (n = 6) and normal controls (n = 4). (D) Relative luciferase activity of the *IL11* promoter in HepG2 cells after transfection with shMCM7 (left) or pHAGE-MCM7 (right). (E) Relative luciferase activity of the *IL11* promoter in HepG2 cells transfected with shSHCBP1 (left) or pHAGE-SHCBP1 (right). (F) Relative luciferase activity of the *IL11* promoter in MCM7 knockdown HepG2 cells transfected with pHAGE-SHCBP1 for 24 hours. Data are presented as mean ± SD of 3–6 per group and are representative of three independent experiments. Statistical analyses were performed using unpaired Student's t-test and one-way ANOVA. *P < 0.05; **P < 0.01; ***P < 0.001; ****P < 0.0001; ns, not significant.

Supplementary figure 15

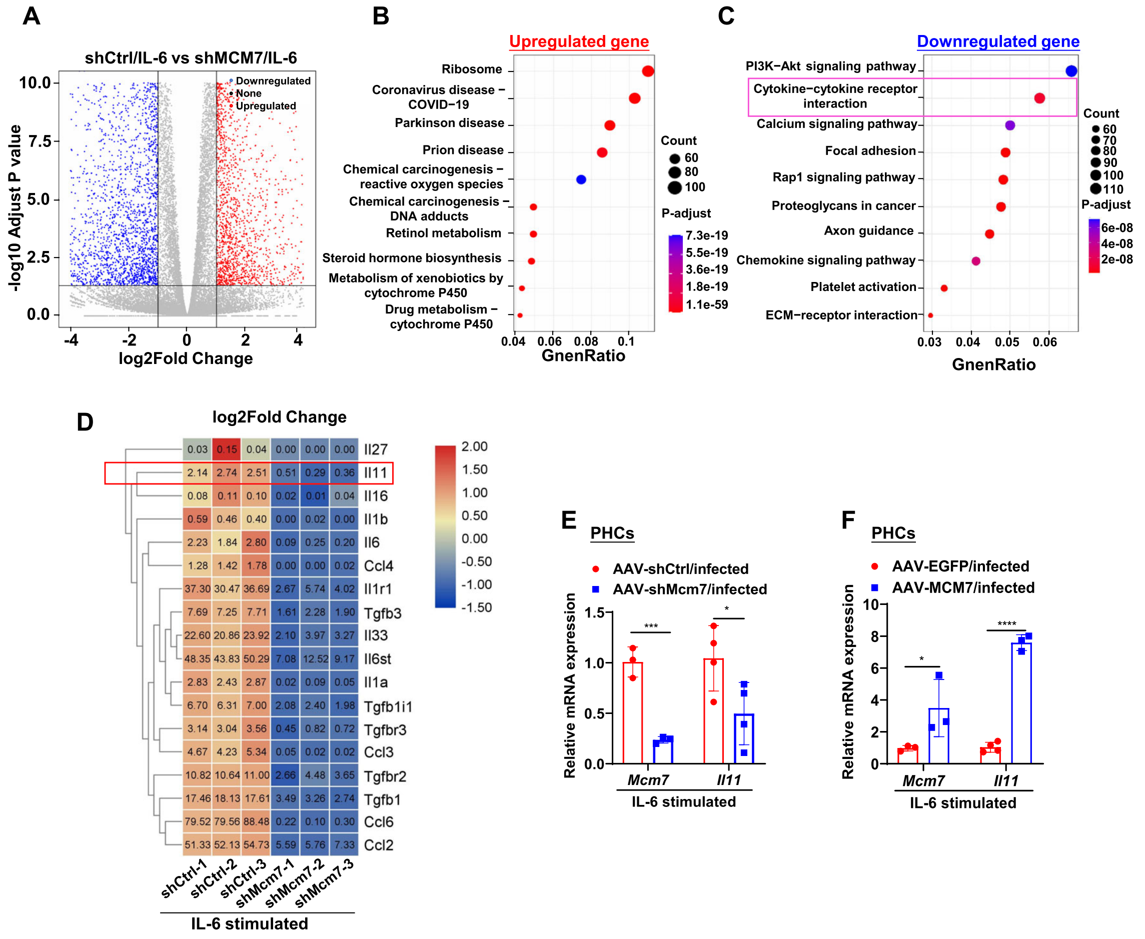

Figure S15. RNA-Seq analysis in primary hepatocytes isolated from AAV-shMcm7-injected mice following IL-6 stimulation. (A) Volcano plot showing differentially expressed genes in primary hepatocytes with MCM7 knockdown (shMCM7) compared to control (shCtrl) upon IL-6 stimulation. Upregulated genes are highlighted in red, and downregulated genes in blue. (B, C) KEGG pathway enrichment analysis of upregulated (B) and downregulated (C) genes in MCM7 knockdown primary hepatocytes upon IL-6 stimulation. Significantly enriched pathways are indicated. The size of the dots represents the number of genes involved, and the color indicates the P-adjust value. The cytokine−cytokine receptor interaction signaling pathway is highlighted with a red box. (D) Heatmap depicting the log2 fold change of inflammatory cytokines in control and MCM7 knockdown primary hepatocytes upon IL-6 stimulation. The expression of *Il11* is prominently highlighted with a red box. (E, F) *Mcm7* and *Il11* mRNA levels measured by qRT-PCR in primary hepatocytes (PHCs) isolated from mice injected with AAV-shMCM7 (E) or AAV-MCM7 (F) , and stimulated with IL-6. Data are presented as mean ± SD of three independent experiments. Statistical analyses were performed using unpaired Student's t-test and one-way ANOVA. *P < 0.05; ***P < 0.001; ****P < 0.0001.

Supplementary figure 16

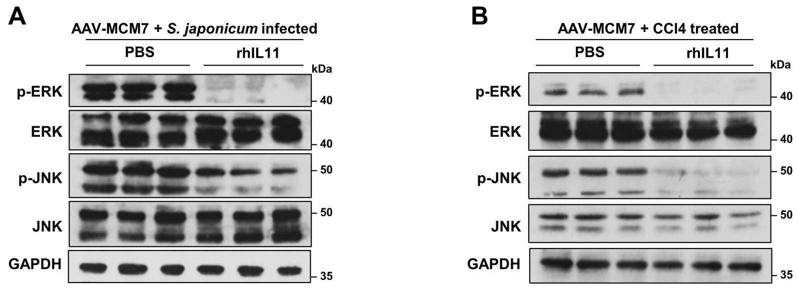

Figure S16. rhIL11 inhibits endogenous IL11 signaling in mice. (A, B) Western blot analysis of phosphorylated ERK, JNK, and their respective total expression in liver tissues from *S. japonicum*-infected (A) and CCl4-treated (B) mice, following injection of AAV-MCM7 and treatment with either rhIL11 or PBS. Data are presented as mean ± SD of 3–6 mice per group and are representative of three independent experiments.

Supplementary figure 17

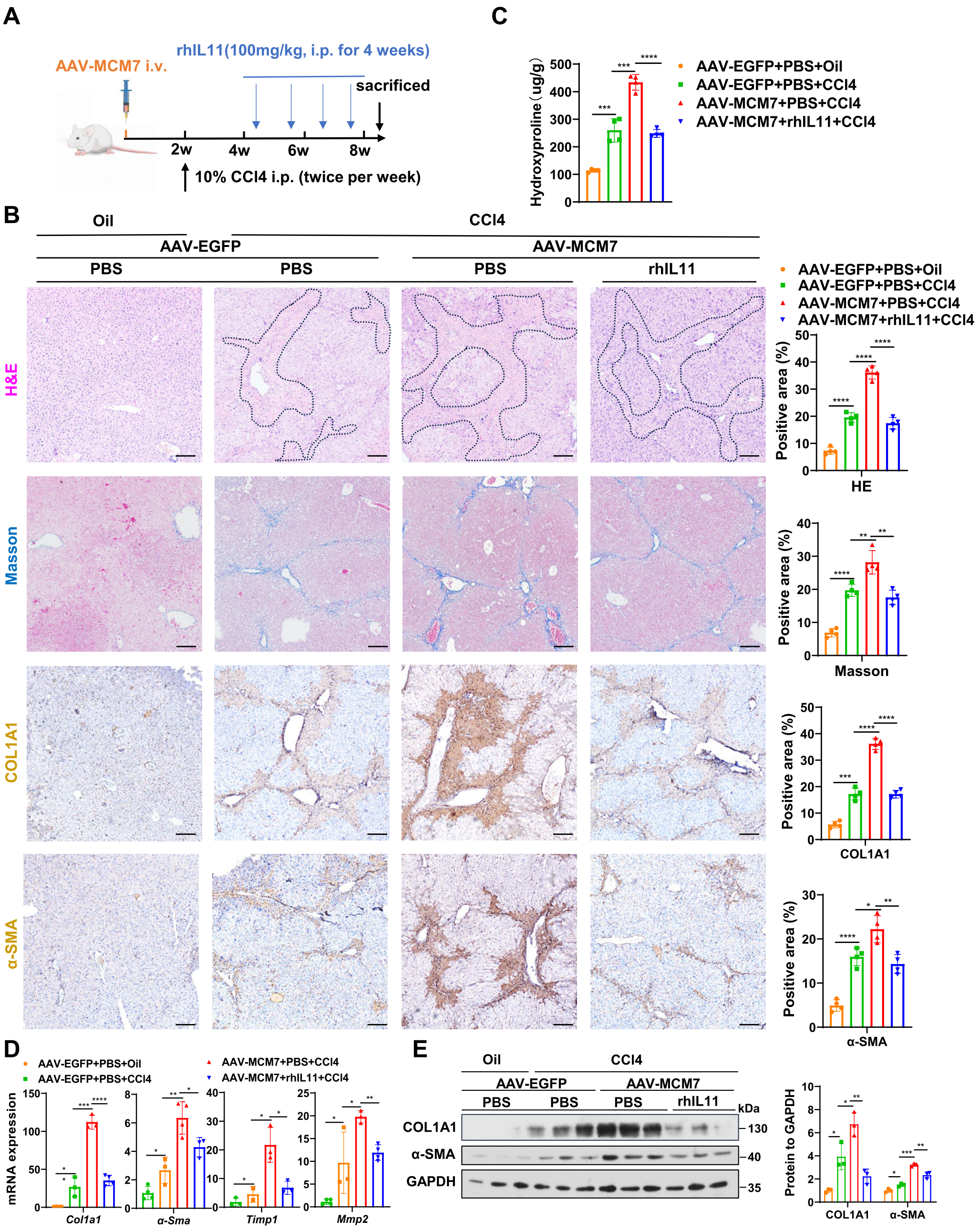

Figure S17. Inhibition of IL11 attenuates MCM7 overexpression-induced liver fibrosis in CCl4-treated mice. (A) Experimental design schematic: mice were intravenously injected with shCtrl or AAV-shMcm7. Two weeks after the AAV injection, mice began receiving intraperitoneal injections of 10% CCl4 twice per week. Four weeks before sacrifice, mice were intraperitoneally treated rhIL11 every two days. Liver samples were collected at 6 weeks post-CCl4 treatment for analysis. (B) H&E staining (areas positive for liver fibrosis are delineated by black dashed lines), Masson's trichrome staining, COL1A1 staining, and α-SMA staining (all scale bars: 100 μm) of liver sections from the indicated groups (AAV-EGFP + PBS + Oil, AAV-EGFP + PBS + CCl4, AAV-MCM7 + PBS + CCl4, AAV-MCM7 + rhIL11 + CCl4). Graphs show the quantified positive areas for each stain, determined using ImageJ software from multiple randomly selected fields across distinct tissue sections. (C) Hydroxyproline content in liver tissues was determined. (D, E) qRT-PCR (D) was used to assess the expression levels of *Col1a1*, *α-Sma*, *Timp1*, and *Mmp2*, while Western blot (E) analysis focused on COL1A1 and α-SMA in liver tissues from the indicated groups, with the graph displaying protein levels. Data are presented as mean ± SD of 3–6 mice per group and are representative of three independent experiments. Statistical analyses were performed using unpaired Student's t-test or one-way ANOVA. *P < 0.05; **P < 0.01; *** P <0.001; ****P < 0.0001; ns, not significant.

Supplementary Table 1: Primer sequences for real-time PCR analysis

Name	Sequence (5'- 3')
m-*MCM7*-F	GCCATTCTCCGACTTTCCAC
m-*MCM7*-R	GGTCCTAGCTGTCTGTCCCTTT
m-*SHCBP1*-F	CCGAGACCTGACTTGGCG
m-*SHCBP1*-R	TTTACAGTAGCACCTGGATTAGCAT
m-*IL11*-F	GGGACATTGGGATCTTTGC
m-*IL11*-R	GGAGTAGCCGTTCCAGTCG
m-*GAPDH*-F	ACTCCACTCACGGCAAATTC
m-*GAPDH*-R	TCTCCATGGTGGTGAAGACA
m-*TIMP1*-F	GGGCTAAATTCATGGGTTCC
m- *TIMP1*-R	CTGGGACTTGTGGGCATATC
m-*Col1a1*-F	GGGGCAAGACAGTCATCGAA
m-*Col1a1*-R	GGG TGGAGGGAGTTTACACG
m-*MMP2*-F	ACAAGTGGTCCGCGTAAAGT
m-*MMP2*-R	AAACAAGGCTTCATGGGGGC
m-*αSMA*-F	GTCCCAGACATCAGGGAGTAA
m-*αSMA*-R	TCGGATACTTCAGCGTCAGGA
h-*Col1a1*-F	GTGGCCCAGAAGAACTGGTA
h-*Col1a1*-R	CGCCATACTCGAACTGGAAT
h-*αSMA*-F	TTCAATGTCCCAGCCATGTA
h-*αSMA*-R	GAAGGAATAGCCACGCTCAG
h-*IL11*-F	GGACAGGGAAGGGTTAAAGG
h-*IL11*-R	CTCAGCACGACCAGGACC
h-*SHCBP1*-F	TGGCACACTAATGTGTTCAAGG
h-*SHCBP1*-R	TCACAGAGGTATGGTTCAGCA
h-*MCM7*-F	TCAGCGTCACTGGTATTTTCTTG
h-*MCM7*-R	TCATC CTCACTCTTGTTCATCTTCA
h-*RACGAP1*-F	ACCTCTTCTGACCTTTCGCC
h-*RACGAP1*-R	CTGAGCCACTCTCTGCAAGT
h-*YAP*-F	TAGCCCTGCGTAGCCAGTTA
h-*YAP*-R	TCATGCTTAGTCCACTGTCTGT
h-*GAPDH*-F	CTGGGCTACACTGAGCACC
h-GAPDH-R	AAGTGGTCGTTGAGGGCAATG
chip-*IL11*(123/-112)-F	TTCCTTCCGTGCCCTCCT
chip-*IL11*(123/-112)-R	AGGGAGGGTGAGTCAGGATGT
chip-*IL11*(-1348/1337)-F	TGGCAAAACCCTATCTCTACTAAAA
chip-*IL11*(-1348/1337)-R	GGGAGGCGGAGGTTACAGT
chip-*IL11*(-1773/-1762)-F	TGCCACGATGAGGATAAAGCG
chip-*IL11*(-1773/-1762)-R	CCCACTGTCCCGTCTTCACC

Supplementary Table 2: Primer sequences for plasmid construction

Name	Sequence (5'- 3')
m-sh*MCM7*-1-F	CTGATCTGTTGTCTGGTATAAACTCGAGTTTATACCAGA CAACAGATCATTTTTGGGCC
m-sh*MCM7*-1-R	CAAAAATGATCTGTTGTCTGGTATAAACTCGAGTTTATA CCAGACAACAGATCAGAGCT
m-sh*MCM7*-2-F	CTCCATGGTGGAAGGGTTAAATCTCGAGATTTAACCCTT CCACCATGGATTTTTGGGCC
m-sh*MCM7*-2-R	CAAAAATCCATGGTGGAAGGGTTAAATCTCGAGATTTA ACCCTTCCACCATGGAGAGCT
m-*SHCBP1*-1-F	CTGATCTGTTGTCTGGTATAAACTCGAGTTTATACCAGAC AACAGATCATTTTTGGGCC
m-*SHCBP1*-1-R	CAAAAATGATCTGTTGTCTGGTATAAACTCGAGTTTATAC CAGACAACAGATCAGAGCT
m-*SHCBP1*-2-F	CTCCATGGTGGAAGGGTTAAATCTCGAGATTTAACCCTT CCACCATGGATTTTTGGGCC
m-*SHCBP1*-2-R	CAAAAATCCATGGTGGAAGGGTTAAATCTCGAGATTTAA CCCTTCCACCATGGAGAGCT
m-AAV-*MCM7*-F	CGGGGTACCCCATGATGGAGCAGCGAAGCAGAG
m-AAV-*MCM7*-R	CGCGTCGACTCAGACAAAGGTGATCCGTGTCC
m-AAV-*SHCBP1*-F	CGGGGTACCATGGCTGATGATTTGCG
m-AAV-*SHCBP1*-R	CGCGTCGACCTAGTAAAGAAATGTGCCAAAAC
h-sh*MCM7*-1-F	CCGGGAGTTGGTGGACTCAATTTCTCGAGAAATTGAGTC CACCAACTCTTTTTG
h-sh*MCM7*-1-R	AATTCAAAAAGAGTTGGTGGACTCAATTTCTCGAGAAA TTGAGTCCACCAACTC
h-sh*MCM7*-2-F	CCGGGGACTCAATTTGTGAGAATCTCGAGATTCTCACAA ATTGAGTCCACTTTTTG
h-sh*MCM7*-2-R	AATTCAAAAAGGACTCAATTTGTGAGAATCTCGAGATTC TCACAAATTGAGTCCAC
h-sh*SHCBP1*-1-F	CCGGCCAATTACAGTGAGTCTGATTCTCGAGAATCAGA CTCACTGTAATTGGTTTTTG
h-sh*SHCBP1*-1-R	AATTCAAAAACCAATTACAGTGAGTCTGATTCTCGAGA ATCAGACTCACTGTAATTGG
h-sh*SHCBP1*-2-F	CCGGCTTGGTGAAACCTACAATCTTCTCGAGAAGATTGT AGGTTTCACCAAGTTTTTG
h-sh*SHCBP1*-2-R	AATTCAAAAACTTGGTGAAACCTACAATCTTCTCGAGA AGATTGTAGGTTTCACCAAG
h- sh*YAP* -1-F	CCGGGGATACAGGTGATACTATCAACTCGAGTTGATAG TATCACCTGTATCC TTTTTG
h-sh*YAP*-1-R	AATTCAAAAAGGATACAGGTGATACTATCAACTCGAGT TGATAGTATCACCTGTATCC
h-sh*YAP*-2-F	CCGGCTCAGGATGGAGAAATTTACTCGAGTAAATTTCT CCATCCTGAG TTTTTG
h-sh*YAP*-2-R	AATTCAAAAACTCAGGATGGAGAAATTTACTCGAGTAA ATTTCTCCATCCTGAG
FLAG-*MCM7*-F	CTAGCTAGCATGGTGGTGGCCACTTACAC
FLAG-*MCM7*-R	TGCTCTAGATCAGACAAAAGTGATCCGTGTC
HA-*MCM7*-F	CCGGAATTCATGGTGGTGGCCACTTACAC
HA-*MCM7*-R	CGGGGTACCCGACAAAAGTGATCCGTGTCCG
FLAG-*MCM_N*-F	CGGGGTACCAAGGAAAAGGTTAAGAAGTTCTT

FLAG-*MCM_N*-R	GCTCTAGAGCGCCTTGAAAATACAGCTCAA
FLAG-*AAA*-F	CGGGGTACCGGCAACATCAACATCTGTCTGATG
FLAG-*AAA*-R	GCTCTAGAGCGTCGGGCCGGTCCTGAAT
FLAG-*SHCBP1*-F	CGCGGATCCATGGCTGACGGGTCGCTG
FLAG-*SHCBP1*-R	CTAGCTAGCTCAGAAAAGAAATGTGCCAAAACTAC
HA-*SHCBP1*-F	CGGGGTACCATGGCTGACGGGTCGCTG
HA-*SHCBP1*-R	CCGCTCGAGGAAAAGAAATGTGCCAAAACTACCTT
GST-*MCM7*-F	CCGGAATTCATGGCACTGAAGGACTACG
GST-*MCM7*-R	ACGCGTCGACTCAGACAAAAGTGATCCGTGTC
GST- *MCM_N* -F	CCGGAATTCAAGGAAAAGGTTAAGAAGTTCTT
GST- *MCM_N* -R	ACGCGTCGACGCCTTGAAAATACAGCTCAA
GST- *AAA* -F	CCGGAATTCGGCAACATCAACATCTGTCTGATG
GST- *AAA* -R	ACGCGTCGACGTCGGGCCGGTCCTGAAT
His-*SHCBP1*-F	CCCAAGCTTATGGCTGACGGGTCGCTG
His-*SHCBP1*-R	CCGCTCGAGGAAAAGAAATGTGCCAAAACTACCT
His-(1-562aa)-F	CCCAAGCTTATGGCTGACGGGTCGCTG
His-(1-562aa)-R	CCGCTCGAGTCCATCTTCAGCATTTTCTTGCA
His-(64-562aa)-F	CCCAAGCTTAAAACCTTTTTCCCAGAAATTTTCC
His-(64-562aa)-R	CCGCTCGAGTCCATCTTCAGCATTTTCTTGCA
His-(291-562aa)-F	CCCAAGCTTGAGATGGAACAGTTGAAACAAAAGC
His-(291-562aa)-R	CCGCTCGAGTCCATCTTCAGCATTTTCTTGCA
His-(355-562aa)-F	CCCAAGCTTGAGCCTGGTGAGGAAGAAAGAGA
His-(355-562aa)-R	CCGCTCGAGTCCATCTTCAGCATTTTCTTGCA
His-(64-210aa)-F	CCCAAGCTTAAAACCTTTTTCCCAGAAATTTTCC
His-(64-210aa)-R	CCGCTCGAGATCCCAACTCCTCCAAATGTTTT
FLAG-*RACGAP1*-F	CGGGGTACCATGGATACTATGATGCTGAATGTG
FLAG-*RACGAP1*-R	CCGCTCGAGTCACTTGAGCATTGGAGAAGC
pGL3-*MCM7*-F	CGGGGTACCCTACCCCGAGAGGGAGTC
pGL3-*MCM7*-R	CCCAAGCTTCAGCTTTCTTTCCACCCC
pGL3-*MCM7*-mut-F	TTCGCGCTTTTGGGCTCGCCCAGGTTCCCGCG
pGL3-*MCM7*-mut-R	CGCGGGAACCTGGGCGAGCCCAAAAGCGCGAA
pGL3-*IL11*-F	CGGGGTACCCTGGCCCACTGTGAGC
pGL3-*IL11*-R	CTAGCTAGCGAGGGAGTGTGGACGC
pGL3-*IL11*-mut-F	CAGGCATGGTGGCGGAGAGGTCGTTGTCCCAGCTACTC AGG
pGL3-*IL11*-mut-R	CCTGAGTAGCTGGGACAACGACCTCTCCGCCACCATGCC TG

Supplementary Table 3: Antibodies for immunoblot.

Antibody	Cat. Number	Company
Anti-MCM7	11225-1-AP	Proteintech
Anti-MCM7	67446-1-Ig	Proteintech
Anti-SHCBP1	12672-1-AP	Proteintech
Anti-RACGAP1	13739-1-AP	Proteintech
Anti-IL11	163801	Zenbio
Anti-COL1A1	67288-1-Ig	Proteintech
Anti-α-SMA	14395-1-AP	Proteintech
Anti-Albumin	16475-1-AP	Proteintech
Anti-F4/80	29414-1-AP	Proteintech
Anti-FLAG	66008-4-Ig	Proteintech
Anti-FLAG	20543-1-AP	Proteintech
Anti-HA	2063	DIAAN
Anti-HA	51064-2-AP	Proteintech
Anti-p-STAT3 (Tyr705)	9131S	CST
Anti-STAT3	10253-2-AP	Proteintech
Anti-GST	66001-2-Ig	Proteintech
Anti-His	66005-1-Ig	Proteintech
Anti-p-ERK1(T202/Y204) +ERK2(T185/Y187)	T40072	Abmart
Anti-ERK1/2	T40071	Abmart
Anti- p-JNK1/2/3 (Thr183+Tyr185)	T40074	Abmart
Anti- JNK1/2/3	T40073	Abmart
Anti- IgG	B900610	Proteintech
Anti-GAPDH	60004-1-Ig	Proteintech
Anti-IL11 Neutralizing Antibody	MAB218	R&D Systems
Anti- IgG type 2a	MAB003	R&D Systems
HRP-goat anti-mouse IgG (H+L)	BF03001	Biodragon

HRP-goat anti- rabbit IgG (H+L)	BF03008	Biodragon
Cy3–conjugated Affinipure Goat Anti-Rabbit IgG(H+L)	SA00009-2	Proteintech
Fluorescein (FITC)–conjugated Affinipure Goat Anti-Rabbit IgG(H+L)	SA00003-2	Proteintech

Supplementary Table 4: Health status of mice in *S. japonicum* - and CCl4-induced liver fibrosis models

AAV8-mediated knockdown or overexpression of MCM7 in *S. japonicum* -induced liver fibrosis models

Group	Body Weight (Mean ± SD)	Liver Weight (Mean ± SD)	liver-to-body weight ratio (Mean ± SD)
AAV-shCtrl/uninfected	25.4 ± 1.2 g	1.2 ± 0.2 g	4.7 ± 0.8%
AAV-shMcm7/uninfected	26.2 ± 1.5 g	1.3 ± 0.1 g	5.0 ± 0.6%
AAV-shCtrl/infected	27.4 ± 1.4 g	1.8 ± 0.2 g	6.6 ± 0.8%
AAV-shMcm7/infected	27.6 ± 1.8 g	1.7 ± 0.2 g	6.2 ± 0.9%

Group	Body Weight (Mean ± SD)	Liver Weight (Mean ± SD)	liver-to-body weight ratio (Mean ± SD)
AAV-EGFP/uninfected	27.2 ± 1.6 g	1.2 ± 0.2 g	4.4 ± 0.7%
AAV-MCM7/uninfected	28.1 ± 1.8 g	1.4 ± 0.1 g	5.0 ± 0.6%
AAV-EGFP/infected	29.4 ± 2 g	2.1 ± 0.3 g	7.1 ± 1.2%
AAV-MCM7/infected	28.3 ± 1.6 g	2.0 ± 0.2 g	7.1 ± 0.9%

AAV8-mediated knockdown or overexpression of MCM7 in CCl4-induced liver fibrosis models

Group	Body Weight (Mean ± SD)	Liver Weight (Mean ± SD)	liver-to-body weight ratio (Mean ± SD)
AAV-shCtrl/Oil	25.0 ± 1.3 g	1.1 ± 0.2 g	4.4 ± 0.8%
shMcm7/Oil	26.1 ± 1.2 g	1.3 ± 0.1 g	5.0 ± 0.6%
AAV-shCtrl/CCl4	25.1 ± 1.1 g	1.6 ± 0.1 g	6.4 ± 0.6%
AAV-shMcm7/CCl4	26.1 ± 1.4 g	1.6± 0.2 g	6.1 ± 0.9%

Group	Body Weight (Mean ± SD)	Liver Weight (Mean ± SD)	liver-to-body weight ratio (Mean ± SD)
AAV-EGFP/Oil	27.1 ± 1.4 g	1.1 ± 0.1 g	4.1 ± 0.5%
AAV-MCM7/Oil	26.6 ± 1.1 g	1.2 ± 0.1 g	4.5 ± 0.5%
AAV-EGFP/CCl4	26.4 ± 2.2 g	1.7 ± 0.3 g	6.4 ± 1.3%
AAV-MCM7/CCl4	27.3 ± 1.8 g	1.8 ± 0.2 g	6.6 ± 1.0%

AAV8-mediated knockdown or overexpression of SHCBP1 in *S. japonicum* -induced liver fibrosis models

Group	Body Weight (Mean ± SD)	Liver Weight (Mean ± SD)	liver-to-body weight ratio (Mean ± SD)
AAV-shCtrl/uninfected	25.8 ± 1.3 g	1.1 ± 0.1 g	4.3 ± 0.6%
AAV-shShcbp1/uninfected	24.2 ± 1.5 g	1.1 ± 0.2 g	4.6 ± 1.0%
AAV-shCtrl/infected	27.2 ± 1.3 g	1.6 ± 0.2 g	5.9 ± 0.8%
AAV-shShcbp1/infected	28.3 ± 1.8 g	1.7 ± 0.3 g	6.0 ± 1.2%

Group	Body Weight (Mean ± SD)	Liver Weight (Mean ± SD)	liver-to-body weight ratio (Mean ± SD)
AAV-EGFP/uninfected	25.3 ± 1.2 g	1.1 ± 0.2 g	4.4 ± 0.9%
AAV-SHCBP1/uninfected	24.8 ± 1.3 g	1.2 ± 0.1 g	4.8 ± 0.6%
AAV-EGFP/infected	27.7 ± 1.5 g	1.6 ± 0.2 g	5.8 ± 0.9%
AAV-SHCBP1/infected	28.1 ± 1.2 g	1.7 ± 0.3 g	6.1 ± 1.2%

AAV8-mediated knockdown or overexpression of SHCBP1 in CCl4-induced liver fibrosis models

Group	Body Weight (Mean ± SD)	Liver Weight (Mean ± SD)	liver-to-body weight ratio (Mean ± SD)
AAV-shCtrl/Oil	28.1 ± 1.6 g	1.1 ± 0.2 g	3.9 ± 0.7%
shShcbp1/Oil	27.6 ± 1.3 g	1.2 ± 0.2 g	4.3 ± 0.8%
AAV-shCtrl/CCl4	29.4 ± 1.2 g	1.9 ± 0.4 g	6.5 ± 1.4%
AAV-shShcbp1/CCl4	28.6 ± 1.4 g	1.8 ± 0.3 g	6.3 ± 1.1%

Group	Body Weight (Mean ± SD)	Liver Weight (Mean ± SD)	liver-to-body weight ratio (Mean ± SD)
AAV-EGFP/Oil	27.8 ± 1.5 g	1.2 ± 0.2 g	4.3 ± 0.7%
AAV-SHCBP1/Oil	26.4 ± 1.5 g	1.0 ± 0.3 g	3.8 ± 1.2%
AAV-EGFP/CCl4	28.0 ± 1.3 g	1.8 ± 0.4 g	6.4 ± 1.4%
AAV-SHCBP1/CCl4	28.3 ± 1.2 g	1.7 ± 0.2 g	6.0 ± 0.8%

rhIL11 treatment in AAV8-mediated MCM7 overexpression in *S. japonicum* - and CCl4-induced liver fibrosis models

Group	Body Weight (Mean ± SD)	Liver Weight (Mean ± SD)	liver-to-body weight ratio (Mean ± SD)
AAV-EGFP + PBS + uninfected	27.3 ± 1 g	1.1 ± 0.1 g	4.0 ± 0.4%
AAV-EGFP + PBS + infected	26.2 ± 1.6 g	1.8 ± 0.3 g	6.9 ± 1.3%
AAV-MCM7 + PBS + infected	26.7 ± 1.8 g	2.0 ± 0.2 g	7.5 ± 1.1%
AAV-MCM7 + rhIL11 + infected	27.3 ± 2 g	1.7 ± 0.3 g	6.2 ± 1.3%

Group	Body Weight (Mean ± SD)	Liver Weight (Mean ± SD)	liver-to-body weight ratio (Mean ± SD)
AAV-EGFP + PBS + Oil	28.4 ± 1.3 g	1.2 ± 0.2 g	4.2 ± 0.7%
AAV-EGFP + PBS + CCl4	27.1 ± 1.5 g	2.0 ± 0.4 g	7.4 ± 1.6%
AAV-MCM7 + PBS + CCl4	27.3 ± 1.4 g	2.1 ± 0.4 g	7.7 ± 1.5%
AAV-MCM7 + rhIL11 + CCl4	28.9 ± 1.3 g	2.1 ± 0.3 g	7.3 ± 1.2%

Supplementary Table 5. Proteins interacting with MCM7 in HL7702 cells as determined by mass spectrometry analysis of MCM7 or IgG immunoprecipitations co-immunoprecipitations; p-values and permutation-based false discovery rate (FDR) calculated by one-tailed t-test

Gene_Name	Sq	Score	Spectra Mass	Q value	Delta Mass (PPM)	Protein AC	Number of PSMs	Unique peptide
MCM7	MALKDYALEKEK	0.0515	1349.73	0	-0.21	sp_P33993	353	64
MCMBP	NSTFTEHLYR	1.37E-01	1324.63	0.0015	2.67	sp_Q9BTE3	83	26
MCM2	QLVAEQVTYQR	9.45E-02	1334.71	0.00067	-0.88	sp_P49736	58	20
CT2NL	NTVTQVLSR	2.71E-02	1017.57	0	0.88	sp_Q9P2B4	35	16
SHCBP	AYQDYILADCK	3.03E-01	1359.63	0.0066	0.93	sp_Q8NEM2	30	12
DLG5	LSLDLSHR	1.60E-01	940.53	0.00176	5.34	sp_Q8TDM6	22	10
GYS1	RIGLFNSSADRVK	3.76E-01	1462.82	0.00867	8.17	sp_P13807	22	9
PEX6	LAVTELR	2.29E-01	801.48	0.00441	2.62	sp_Q13608	19	9
PTN13	SLNLQAESVR	3.48E-02	1116.6	0	0.94	sp_Q12923	17	12
PEX1	NVEVLHLGK	2.90E-01	1008.58	0.00629	0.34	sp_O43933	16	9
CACO1	APSRGGVNFLNVAR	3.66E-01	1457.8	0.00844	-0.73	sp_Q9P1Z2	15	9
STRN	CYIASAGADALAK	1.14E-01	1310.64	0.0011	2.41	sp_O43815	15	8
PUR1	FGVLSDNFK	2.18E-01	1026.53	0.00438	0	sp_Q06203	13	5
DIXC1	GTSDLQLVR	7.29E-02	988.54	0.00018	-0.09	sp_Q155Q3	12	6
CCD51	VSREDSQYLELATLEHR	1.51E-01	2046.03	0.00163	0.54	sp_Q96ER9	12	6
GANAB	GLLEFEHQR	8.91E-02	1128.58	0.00052	-0.2	sp_Q14697	11	5
STRN4	ALIASAGADALAK	1.95E-01	1171.67	0.0038	1.67	sp_Q9NRL3	10	7
XPO2	IIIPEIQK	1.42E-01	953.6	0.0015	1.22	sp_P55060	10	6
GDE	DGSAVEIVGLSK	6.76E-03	1174.63	0	1.23	sp_P35573	9	5
PCCB	NIIVGFAR	9.90E-02	889.53	0.00067	1.15	sp_P05166	8	4
RS7	ELNITAAKEIEVGGGRK	1.60E-01	1784.99	0.00176	3.54	sp_P62081	8	6
PNPT1	VLQSPATTVVR	1.52E-01	1170.68	0.00163	0.47	sp_Q8TCS8	8	6
MYOF	GPVGTVSEAQLAR	1.42E-01	1284.69	0.0015	-1.49	sp_Q9NZM1	8	5
HSP74	CTPACISFGPK	2.66E-01	1237.57	0.00526	0.38	sp_P34932	8	5
AIMP2	SVPENLLK	2.37E-01	899.52	0.00441	1.95	sp_Q13155	8	3
G3BP1	TFSWASVTSK	6.50E-02	1113.56	0.00018	2.03	sp_Q13283	8	3
COX5B	KGLDPYNVLAPK	5.72E-02	1314.74	0	2.08	sp_P10606	8	2
ACTB	MDDDIAALVVDNGSGMCK	4.28E-03	1773.8	0	0.99	sp_P60709	8	2
CCAR1	AGLLQPPVR	1.67E-01	950.58	0.00258	2.48	sp_Q8IX12	7	2
RO52	NFLVEEEQR	1.76E-02	1163.57	0	1.03	sp_P19474	7	4
STRP1	LLDGLEVTAR	2.61E-02	1086.61	0	-0.55	sp_Q5VSL9	7	4
TARB1	SIVQEYVK	3.25E-01	965.53	0.00758	2.44	sp_Q13395	7	6
WNK1	VAIPEVK	1.81E-01	755.47	0.00333	-0.35	sp_Q9H4A3	7	5
SYWC	ELINRIER	4.86E-02	1043.59	0	1.13	sp_P23381	7	4
KAD2	AVLLGPPGAGK	3.86E-01	979.6	0.00959	1.64	sp_P54819	7	3
PSA6	AINQGGLTSVAVR	2.89E-03	1285.72	0	-1.31	sp_P60900	7	3
TBB4A	TAVCDIPPR	1.18E-01	1028.52	0.0011	0.55	sp_P04350	7	2
PKHH1	VLQSLLK	1.29E-01	800.52	0.00124	-0.29	sp_Q9ULM0	7	1
TBA4A	QLFHPEQLITGKEDAANNYAR	5.88E-03	2415.21	0	2.67	sp_P68366	7	1
9-Sep	VVNIVPVIAK	3.55E-01	1051.69	0.00835	1.01	sp_Q9UHD8	6	4
SRRT	AEIISLCK	1.69E-01	933.51	0.00282	1.35	sp_Q9BXP5	6	4
TCPD	VIDPATATSVDLR	1.35E-01	1357.73	0.0015	-0.27	sp_P50991	6	5
CPT1A	LAALTAGDRVPWAR	3.84E-01	1496.83	0.00943	0.29	sp_P50416	6	5
CSTF3	CKIPNTVEEAVR	9.27E-03	1398.71	0	2.81	sp_Q12996	6	4
SYDM	IPEFSSFVVR	4.82E-02	1180.64	0	4.04	sp_Q6PI48	6	4
CSTF2	GSLPANVPTPR	1.81E-01	1108.62	0.00333	4.32	sp_P33240	6	3
PSD11	VIEPFSR	3.07E-01	847.47	0.00697	0.66	sp_O00231	6	3
REPS1	TVASATTAIEIR	5.09E-02	1232.69	0	2.52	sp_Q96D71	6	3
S10A4	SELKELLTR	3.80E-01	1088.63	0.00881	3.59	sp_P26447	6	3
RBM39	VLGVPIIVQASQAEKNR	3.23E-02	1822.06	0	2.63	sp_Q14498	6	3
CD44	ALSIGFETCR	1.96E-01	1153.57	0.0038	-0.05	sp_P16070	6	2
E2AK3	GGFGVVFEAK	2.18E-01	1010.53	0.00438	0.1	sp_Q9NZJ5	6	2
TBB1	LAVNMVPFPR	3.64E-02	1095.63	0	0.27	sp_Q9H4B7	6	1
GCP5	EDSGIQVDR	3.70E-01	1018.48	0.00844	-2.37	sp_Q96RT8	6	1
EIF3A	RLEEIPLIK	3.39E-01	1110.69	0.00778	2.51	sp_Q14152	5	4
EGFR	ITDFGLAK	4.30E-02	864.48	0	2.52	sp_P00533	5	3
HXK1	ITPELLTR	1.26E-01	942.56	0.0011	1.29	sp_P19367	5	3
FSCN1	LVARPEPATGYTLEFR	3.51E-02	1819.97	0	0.34	sp_Q16658	5	3
TOM34	ALCYLVLK	2.98E-01	979.57	0.0066	3.07	sp_Q15785	5	3
SYSC	LLIDEAILKCDAER	2.44E-01	1658.88	0.00441	0.67	sp_P49591	5	3
PSA	LNLGTVGFYR	3.05E-01	1139.62	0.0066	2	sp_P55786	5	3
HINT1	CAADLGLNKGYR	1.95E-03	1320.64	0	2.15	sp_P49773	5	2
CISY	IVPNVLLEQGK	3.53E-02	1209.72	0	1.89	sp_O75390	5	2
2AAB	TSACGLFSVCYPR	4.30E-03	1517.69	0	2	sp_P30154	5	2
PYRG1	GLGLSPDLVVCR	2.05E-03	1285.69	0	0.96	sp_P17812	5	2
RS29	DIGFIKLD	2.03E-01	920.51	0.00414	0.23	sp_P62273	5	2
GTR1	TFDEIASGFR	3.73E-01	1142.55	0.00859	2.6	sp_P11166	5	1
LYAM3	TLLALLR	1.07E-01	799.54	0.00082	0.93	sp_P16109	5	1
NCKPL	IYLTLLR	1.89E-01	891.57	0.00369	-0.15	sp_P55160	5	1
ACTG	CDVDIRK	1.23E-01	888.42	0.0011	-0.61	sp_P63261	5	1
SYRC	LMDLLGEGLKR	3.29E-01	1196.7	0.00771	1.08	sp_P54136	4	4
CTND1	GSLASLDSLRK	2.60E-01	1146.65	0.0048	2.87	sp_O60716	4	4
PA2G4	AFFSEVER	3.44E-01	984.48	0.00778	1.64	sp_Q9UQ80	4	4
SYIC	TQLKGSELEITLTR	3.50E-01	1588.89	0.00796	-0.58	sp_P41252	4	3
LRC47	NALGPGLSPELGPLPALR	2.83E-03	1772.02	0	7.91	sp_Q8N1G4	4	3
UBP10	QALGDKIVR	2.09E-01	982.57	0.0042	2.76	sp_Q14694	4	3
STRN3	AYIASAGADALAK	1.05E-01	1221.65	0.00082	-1.43	sp_Q13033	4	3
SF3B1	VQENCIDLVGR	2.90E-01	1302.65	0.00629	1.75	sp_O75533	4	3
SERPH	AVAISLPK	1.93E-01	798.51	0.0038	0.09	sp_P50454	4	3
PSB6	QVLLGDQIPK	3.79E-01	1110.66	0.00881	5.1	sp_P28072	4	3
SAHH	MSDKLPYK	9.03E-02	892.48	0.00052	0.04	sp_P23526	4	3
CLPX	LLQDANYNVEK	1.99E-01	1306.67	0.0038	1.3	sp_O76031	4	3
RS6	DIPGLTDTTVPR	1.13E-01	1284.67	0.00082	-3.76	sp_P62753	4	3
MCCB	AFYGDTLVTGFAR	4.41E-03	1417.71	0	1.66	sp_Q9HCC0	4	3
CMC2	LQVAGEITTGPR	2.71E-02	1241.69	0	0.86	sp_Q9UJS0	4	2
RB6I2	AAGLQAEIGQVK	2.69E-01	1184.66	0.00526	0.23	sp_Q8IUD2	4	2
HS90B	ELISNASDALDK	2.81E-01	1275.65	0.00601	2.46	sp_P08238	4	2
TERA	KGDIFLVR	5.59E-02	947.57	0	-0.25	sp_P55072	4	2
EIF2A	APSTPLLTVR	1.71E-01	1054.63	0.00282	0.8	sp_Q9BY44	4	2
6PGL	ELPAAVAPAGPASLAR	1.87E-01	1490.83	0.00345	-4.43	sp_O95336	4	2
AT1B1	VAPPGLTQIPQIQK	2.24E-01	1489.87	0.00438	0.72	sp_P05026	4	2
TMCO1	ILGLAPSR	3.09E-01	826.51	0.00697	-0.3	sp_Q9UM00	4	1
STRN	IAFLQGER	3.69E-01	933.52	0.00844	1.14	sp_O43815	4	1
GFPT2	GSPLLIGVR	3.22E-01	911.57	0.0073	0.05	sp_O94808	4	1
GYS1	VGGIYTVLQTK	1.88E-01	1178.68	0.00369	0.02	sp_P13807	4	1
GYS2	VGGIYTVLQTK	1.88E-01	1178.68	0.00369	0.02	sp_P13807	4	1
CAH6	EILDYLR	2.64E-01	921.51	0.00518	1.53	sp_P23280	4	1
POTEI	VAPEEHPILLTEAPLNPK	3.57E-01	1968.09	0.00841	2.89	sp_P0CG38	4	1
TBB6	LHFFMPGFAPLTSR	1.92E-03	1572.83	0	1.71	sp_Q9BUF5	4	1
PDC6I	CSDIVFAR	3.32E-01	967.47	0.00778	-0.26	sp_Q8WUM4	3	3
AAAT	STEPELIQVK	3.33E-02	1143.62	0	-0.54	sp_Q15758	3	3
CMC2	ASGDSARPVLLQVAESAYR	3.93E-01	1990.04	0.00981	1.59	sp_Q9UJS0	3	3
DDB1	IGRPSETGIIGIIDPECR	2.75E-01	1983.04	0.00544	1.19	sp_Q16531	3	3
PSB2	FILNLPTFSVR	4.24E-02	1306.76	0	3.71	sp_P49721	3	3
RL15	SLQSVAEERAGR	4.83E-02	1302.68	0	2.99	sp_P61313	3	3
STRAP	CVLPEEDSGELAKPK	2.10E-01	1671.83	0.0042	-0.36	sp_Q9Y3F4	3	2
ITB1	SGEPQTFTLK	1.03E-01	1107.57	0.00082	2.68	sp_P05556	3	2
SRBP2	AVLLAEAAR	8.69E-02	913.55	0.00052	0.45	sp_Q12772	3	2
STRP1	ILLAAAPTSK	3.61E-01	984.61	0.00844	1.21	sp_Q5VSL9	3	2
RAVR1	VSFCAPGPPGR	3.15E-01	1144.56	0.00702	0.68	sp_Q8IY67	3	2
RBM4	SLFEQYGK	1.96E-01	971.48	0.0038	0.97	sp_Q9BWF3	3	2
TCP4	EQISDIDDAVR	7.99E-02	1260.61	0.00052	3.43	sp_P53999	3	2
PSA1	ALRETLPAEQDLTTK	1.63E-02	1685.91	0	2.45	sp_P25786	3	2
DHX30	TAVEFLSK	3.05E-01	894.49	0.0068	-2.05	sp_Q7L2E3	3	2
RL9	GVTLGFR	2.57E-01	749.43	0.0047	2.08	sp_P32969	3	2
TBG2	VIHSILNSPYAK	2.50E-01	1341.75	0.00456	-0.29	sp_Q9NRH3	3	2
MYG1	YALTTTLSAR	4.90E-02	1096.6	0	0.81	sp_Q9HB07	3	2
VDAC1	YQIDPDACFSAK	4.76E-02	1414.63	0	-0.02	sp_P21796	3	2
HSDL2	LAGCTVFITGASR	1.07E-01	1352.7	0.00082	1.28	sp_Q6YN16	3	2
VPS35	ILVGTNLVR	2.03E-01	984.62	0.00414	-1.04	sp_Q96QK1	3	2
SBDS	FILPVNEGK	1.01E-01	1016.58	0.00067	0.02	sp_Q9Y3A5	3	2

ICLN	GLGTGTLYIAESR	1.41E-01	1337.71	0.0015	2.2	sp_P54105	3	2
UAP1	FIGFCIQK	3.15E-01	1012.53	0.00702	1.42	sp_Q16222	3	2
NU155	VASVSQNAIVSAAGNIAR	3.44E-01	1727.94	0.00778	0.95	sp_O75694	3	2
EIF3B	FSHQGVQLIDFSPCER	3.47E-01	1919.91	0.00778	0.7	sp_P55884	3	2
IPO7	MDPNTIIEALR	1.80E-01	1266.67	0.00311	2.05	sp_O95373	3	2
SPA5L	ALTALGLAVPR	5.13E-03	1081.67	0	-0.97	sp_Q9BVQ7	3	2
TLN1	AVAEQIPLLVQGVR	9.34E-02	1492.89	0.00067	2.84	sp_Q9Y490	3	2
NDEL1	ISALNIVGDLLR	3.12E-04	1283.77	0	1.99	sp_Q9GZM8	3	2
BHMT1	AIAEELAPER	2.00E-01	1098.58	0.00392	-0.96	sp_Q93088	3	1
ANR24	VAALIAR	3.46E-01	713.47	0.00778	-1.33	sp_Q8TF21	3	1
DYHC2	ALGGLLGR	3.59E-01	756.47	0.00841	1.65	sp_Q8NCM8	3	1
DOCK6	VVELLK	2.41E-01	700.46	0.00441	-0.16	sp_Q96HP0	3	1
COBL1	SLNDLGLR	2.63E-01	887.5	0.00518	0.8	sp_Q53SF7	3	1
ANXA3	LTFDEYR	3.36E-01	943.45	0.00778	1.78	sp_P12429	3	1
TBB4A	AVLVDLEPGTMDSVR	3.22E-01	1553.82	0.0073	1.66	sp_P04350	3	1
SMD3	VAQLEQVYIR	2.07E-01	1218.69	0.0042	4.46	sp_P62318	3	1
RACK1	VWQVTIGTR	1.15E-01	1059.59	0.0011	-5.47	sp_P63244	3	1
TBB4A	IREEFPDRIMNTFSVVPSPK	1.87E-01	2435.24	0.00345	0.53	sp_P04350	3	1
HNRH1	MMLGTEGGEGFVVKVR	1.66E-02	1572.84	0	-0.09	sp_P31943	3	1
IPYR	AAPFSLEYR	6.64E-02	1053.53	0.00018	-1.59	sp_Q15181	3	1
SART3	TQLSLLPR	1.02E-01	927.56	0.00082	1.76	sp_Q15020	3	1
SCMC1	QLLAGGIAGAVSR	5.27E-02	1212.71	0	1.15	sp_Q6NUK1	3	1
CE170	ISQDLALIAR	2.14E-02	1099.65	0	-1.72	sp_Q5SW79	3	1
CATD	VSTLPAITLK	2.56E-02	1042.66	0	4.67	sp_P07339	3	1
WNK4	IGDLGLATLK	6.74E-02	1000.61	0.00018	1.62	sp_Q96J92	3	1
RB6I2	AAILQTEVDALR	4.51E-03	1299.73	0	5.25	sp_Q8IUD2	3	1
IDH3A	IAEFAFEYAR	1.10E-01	1216.6	0.00082	2.71	sp_P50213	3	1
HSP72	LLQDFFNGKELNK	3.75E-01	1566.82	0.00867	0.84	sp_P54652	3	1
SRPRA	NQGFDVVLVDTAGR	2.46E-01	1490.76	0.00441	2.72	sp_P08240	3	1
RCBT1	FSLFSAAVR	1.31E-02	997.55	0	0.84	sp_Q8NDN9	3	1
HBB	VNVDEVGGEALGR	1.40E-02	1302.63	0	-0.16	sp_P68871	3	1
MYH14	LVLDQLR	2.86E-01	856.53	0.00618	0.13	sp_Q7Z406	3	1
HGB1A	IKGEHPGLSIGDVAK	3.70E-01	1520.84	0.00844	-1.01	sp_B2RPK0	3	1

Supplementary Table 6. Proteins interacting with SHCBP1 in HL7702 cells as determined by mass spectrometry analysis of SHCBP1 or IgG immunoprecipitations co-immunoprecipitations; p-values and permutation-based false discovery rate (FDR) calculated by one-tailed t-test

Gene_Name	Sq	Score	Spectra Mass	Q value	Delta Mass (PPM)	Protein AC	Number of PSMs	Unique peptide
MCM5	VLQLMLR	0.214	929.57	0.00285	6.77	sp_P33992	21	12
KIF23	NLQQELETQNQK	0.00603	1472.74	0	6.3	sp_Q02241	19	9
RACGAP1	SIGSAVDQGNESIVAK	0.102	1574.82	0.00056	9.84	sp_Q9H0H5	18	13
RO52	NFLVEEEQR	0.122	1163.58	0.00078	6.19	sp_P19474	17	10
ITA3	YTQVLWSGSEDQRR	0.0603	1724.85	0	6.47	sp_P26006	16	12
RS16	TLLVADPRR	0.413	1040.62	0.00995	3.71	sp_P62249	16	11
MCM3	SKDIFDQLAK	0.185	1164.64	0.00261	9.18	sp_P25205	13	9
CKAP4	SVGELPSTVESLQK	0.0637	1473.79	0	9.78	sp_Q07065	12	9
ITB1	SGEPQTFTLK	0.0836	1107.58	0.00013	7.23	sp_P05556	11	9
SVIL	GEVPVEGNLLEVACHCSSLR	0.367	2226.09	0.00759	12.5	sp_O95425	11	5
MPCP	FACFER	0.322	829.37	0.00549	5.92	sp_Q00325	11	4
HS90A	ELISNSSDALDKIR	0.201	1560.83	0.00273	7.04	sp_P07900	11	9
RL11	YDGIILPGK	0.307	975.56	0.00522	9.09	sp_P62913	10	7
ATD3A	QRYEDQLKQQQLLNEENLR	0.105	2428.25	0.00056	11.45	sp_Q9NVI7	8	6
RS17	VCEEIAIIPSKK	0.0815	1386.77	0.00013	5.29	sp_P08708	8	4
TCPG	AVAQALEVIPR	0.0192	1166.7	0	6.94	sp_P49368	8	4
K2C6A	FLEQQNK	0.221	906.47	0.00285	6.47	sp_P02538	7	1
RS20	TPVEPEVAIHR	0.0129	1247.68	0	7.92	sp_P60866	6	5
H2B1N	LLLPGELAK	0.0773	953.61	0.00013	6.06	sp_Q99877	6	4
1433B	YLSEVASGDNKQTTVSNSQQAYQEAFEISKK	0.0819	3450.68	0.00013	5.06	sp_P31946	6	2
POTEI	VAPEEHPILLTEAPLNPK	0.329	1968.1	0.00569	10.14	sp_P0CG38	6	1
DEST	AVIFCLSADKK	0.369	1251.69	0.00759	10.25	sp_P60981	5	5
ATPO	VAASVLNPYVK	0.414	1160.68	0.00995	6.93	sp_P48047	5	4
HS90A	ADLINNLGTIAK	0.00924	1242.71	0	7.14	sp_P07900	5	3
RSSA	FAAATGATPIAGR	0.103	1203.66	0.00056	6.91	sp_P08865	5	3
ADT3	GNLANVIR	0.147	856.51	0.00158	7.32	sp_P12236	5	3
RS14	IGRIEDVTPIPSDSTRR	0.349	1912.04	0.00685	5.55	sp_P62263	5	3
LV460	FSGSSSGADR	0.258	970.43	0.00374	6.75	sp_A0A075B6I1	5	2
LMNA	VAVEEVDEEGKFVR	0.00787	1605.82	0	7.48	sp_P02545	5	2
ADT3	GLGDCLVK	0.307	861.46	0.00522	7.04	sp_P12236	4	4
SSBP	ESETTTSLVLER	0.12	1364.7	0.00056	8.49	sp_Q04837	4	4
S27A4	YVPLDQEAYSR	0.226	1340.66	0.00285	7.4	sp_Q6P1M0	4	4
DX39A	GLAITFVSDENDAK	0.124	1479.74	0.0012	7.22	sp_O00148	4	3
PDIA1	ILEFFGLKKEECPAVR	0.0779	1936.05	0.00013	7.73	sp_P07237	4	3
ACADM	QREPGLGFSFEFTEQQKEFQATAR	0.188	2831.4	0.00261	8.97	sp_P11310	4	3
ABCD3	KGISDLVLKEYLDNVQLGHILER	0.00181	2652.49	0	7.77	sp_P28288	4	3
RL8	AVVGVVAGGGRIDKPILK	0.114	1749.09	0.00056	7	sp_P62917	4	3
ATD3A	LLSRPQDALEGVVLSPSLEAR	0.000977	2250.26	0	7.2	sp_Q9NVI7	4	3
CING	TLQQLR	0.242	758.46	0.00311	5.15	sp_Q9P2M7	4	3
KPYM	GDLGIEIPAEK	0.0686	1141.62	0	9.53	sp_P14618	4	2
PIMT	VQLVVGDGR	0.0873	942.54	0.00013	7.11	sp_P22061	4	2
DNJA1	TIVITSHPGQIVK	0.183	1392.83	0.00244	7.27	sp_P31689	4	2
TBA1B	LDHKFDLMYAKR	0.274	1488.8	0.0041	5.82	sp_P68363	4	2
PRDX2	QITVNDLPVGR	0.13	1211.68	0.0014	8.38	sp_P32119	4	1
MYH14	CNGVLEGIR	0.0512	1017.52	0	7.48	sp_Q7Z406	4	1
KI18B	SLLALINVLNALADAK	0.00807	1638.99	0	7.25	sp_Q86Y91	4	1
HGB1A	KKFKDPNAPK	0.121	1172.68	0.00056	5.34	sp_B2RPK0	3	3
RS10	IAIYELLFKEGVMVAK	0.00965	1881.07	0	10.12	sp_P46783	3	3
COPA	GITGVDLFGTTDAVVK	0.367	1592.87	0.00759	10.51	sp_P53621	3	3
RS11	DVQIGDIVTVGECRPLSK	0.0129	1986.05	0	7.93	sp_P62280	3	3
YBOX1	SSEAETQQPPAAPPAAPALSAADTKPGT	0.109	4642.3	0.00056	11.69	sp_P67809	3	3
M2OM	AVVVNAAQLASYSQSK	0.0278	1635.88	0	9.03	sp_Q02978	3	3
PDIA6	LAAVDATVNQVLASR	0.00368	1527.86	0	9.62	sp_Q15084	3	3
CAND1	ISGSILNELIGLVR	0.000338	1483.89	0	6.42	sp_Q86VP6	3	3
TITIN	KAPAVVAK	0.328	783.51	0.00569	4.53	sp_Q8WZ42	3	3
CMC2	LAVATFAGIENK	0.0615	1233.7	0	12.85	sp_Q9UJS0	3	3
ARC1B	NSVSQISVLSGGK	0.314	1275.7	0.00522	9.46	sp_O15143	3	2
BAG2	LLESLDQLELRVEALR	0.00771	1897.09	0	7.71	sp_O95816	3	2
RLA1	AAGVNVEPFWPGLFAK	0.00208	1702.91	0	10.03	sp_P05386	3	2
ITAV	AGTQLLAGLR	0.181	999.61	0.00225	11.77	sp_P06756	3	2
HS90B	HSQFLGYPITLYLEK	0.0338	1808.97	0	8.04	sp_P08238	3	2
ANXA7	VLIEILCTR	0.236	1116.65	0.00297	6.3	sp_P20073	3	2
FLNA	KGEITGEVR	0.108	988.55	0.00056	6.73	sp_P21333	3	2
VDAC2	TGDFQLHTNVNDGTEFGGSIYQK	0.0165	2528.19	0	8.33	sp_P45880	3	2
CRIP1	EVYFAER	0.395	913.45	0.00898	7.32	sp_P50238	3	2
RS25	LITPAVVSER	0.0551	1084.65	0	8.92	sp_P62851	3	2
TBA1B	LSVDYGKK	0.105	909.51	0.00056	6.47	sp_P68363	3	2
NSUN2	ILLTQENPFFR	0.146	1377.77	0.00158	9.71	sp_Q08J23	3	2
CMC2	LQVAGEITTGPR	0.0518	1241.69	0	1.65	sp_Q9UJS0	3	2
MYO1C	LLQSNPVLEAFGNAK	0.0218	1600.89	0	10.66	sp_O00159	3	1
GOSR2	RVDQLK	0.371	758.46	0.00759	6.25	sp_O14653	3	1
HMGB3	LKEKYEK	0.388	937.54	0.00879	5.21	sp_O15347	3	1
KCNN4	QIDTLAGKLDALTELLSTALGPR	0.263	2396.36	0.00389	10.45	sp_O15554	3	1
TBB4A	IMNTFSVVPSPK	0.298	1376.73	0.00516	5.88	sp_P04350	3	1
HS71B	STLEPVEK	0.368	902.49	0.00759	6.47	sp_P0DMV9	3	1
H12	GTGASGSFK	0.133	811.4	0.0014	6.28	sp_P16403	3	1
TXTP	TQLQLDER	0.0968	1002.53	0.00056	7.54	sp_P53007	3	1
TBA1B	QLFHPEQLITGKEDAANNYAR	0.0409	2415.22	0	6.96	sp_P68363	3	1
1433F	NLLSVAYK	0.111	907.53	0.00056	6.99	sp_Q04917	3	1
DX39B	CIALAQLLVEQNFPAIAIHR	0.0135	2260.24	0	7.79	sp_Q13838	3	1
H32	KSAPATGGVK	0.1	957.58	0.00056	6.32	sp_Q71DI3	3	1
K1C26	VTMQNLNDR	0.207	1147.56	0.00285	6.84	sp_Q7Z3Y9	3	1
ABCAD	TEEVILK	0.3	831.49	0.00516	7.05	sp_Q86UQ4	3	1
DNHD1	LVQVNR	0.267	728.45	0.00405	6.81	sp_Q96M86	3	1
TBB6	LHFFMPGFAPLTSR	0.18	1677.87	0.00225	5.41	sp_Q9BUF5	3	1
MYO1A	YLGLLENVR	0.169	1076.62	0.00174	7.88	sp_Q9UBC5	3	1
CMC1	GLIPQLIGVAPEK	0.166	1334.82	0.00174	8.68	sp_O75746	2	2
CYTB	VHVGDEDFVHLR	0.0274	1422.72	0	7.28	sp_P04080	2	2
C1TC	YVVVTGITPTPLGEGK	0.0878	1630.92	0.00013	10.92	sp_P11586	2	2
PYRG1	GLGLSPDLVVCR	0.084	1285.71	0.00013	9.89	sp_P17812	2	2
PUR6	IKAEYEGDGIPTVFVAVAGR	0.0942	2092.14	0.00056	15.26	sp_P22234	2	2
RS27	LVQSPNSYFMDVK	0.306	1584.78	0.00522	4.79	sp_P42677	2	2
TCPE	DVDFELIKVEGK	0.00495	1391.76	0	10.87	sp_P48643	2	2
RS13	GLTPSQIGVILR	0.271	1253.77	0.0041	7.09	sp_P62277	2	2
CNBP	GFQFVSSSLPDICYR	0.053	1775.87	0	13.71	sp_P62633	2	2
RL23A	LAPDYDALDVANK	0.0277	1404.71	0	6.95	sp_P62750	2	2
RL10A	FSVCVLGDQQHCDEAK	0.149	1892.84	0.00158	7.81	sp_P62906	2	2
U5S1	YHIEVNRVPAGNWVLIEGVDQPIVK	0.131	2845.56	0.0014	7	sp_Q15029	2	2
MARE1	LTVEDLEKERDFYFGK	0.402	1989.01	0.00947	9.04	sp_Q15691	2	2
NDUA9	SSVSGIVATVFGATGFLGR	0.000197	1826	0	12.26	sp_Q16795	2	2
CE350	LQEIYRK	0.101	949.55	0.00056	6.7	sp_Q5VT06	2	2
DDX1	FLICTDVAAR	0.389	1165.61	0.00879	9.97	sp_Q92499	2	2
DYH9	TLEDSLLSR	0.318	1033.56	0.00549	7.77	sp_Q9NYC9	2	2
COPG2	LAPSRQDIFQEQLAAIPEFLNIGPLFK	0.0618	3055.62	0	-13.07	sp_Q9UBF2	2	2
REV1	NLLLAR	0.303	699.46	0.00516	6.15	sp_Q9UBZ9	2	2
CLIC1	LAALNPESNTAGLDIFAK	0.0102	1844.99	0	7.64	sp_O00299	2	1
IPO5	AAAAAEQQQFYLLLGNLLSPDNVVR	0.0107	2743.46	0	7.33	sp_O00410	2	1
HMGB3	RPPSGFFLFCSEFRPK	0.0157	1972	0	7.58	sp_O15347	2	1
SERA	AGTGVDNVDLEAATR	0.00317	1488.74	0	6.94	sp_O43175	2	1
DEND	AGPLAEVR	0.3	814.48	0.00516	6.21	sp_O94850	2	1
PCNT	RAELEK	0.167	745.42	0.00174	4.76	sp_O95613	2	1
K2C6A	EYQELMNVK	0.222	1210.59	0.00285	8.06	sp_P02538	2	1
K2C6A	NKYEDEINKR	0.0482	1308.66	0	5.91	sp_P02538	2	1
K2C6A	KYEDEINKR	0.197	1194.62	0.00273	5.63	sp_P02538	2	1
TBB4A	TAVCDIPPR	0.121	1028.53	0.00056	9.06	sp_P04350	2	1
ITB1	LGFGSFVEK	0.0649	983.53	0	7.47	sp_P05556	2	1
TPM2	SVAKLEK	0.215	774.48	0.00285	4.96	sp_P07951	2	1
HMGB1	YEKDIAAYR	0.235	1128.58	0.00297	9.87	sp_P09429	2	1
CD44	ALSIGFETCR	0.138	1153.57	0.00158	6.79	sp_P16070	2	1
BTF3	VQASLAANTFTITGHAETK	0.003	1960.03	0	7.63	sp_P20290	2	1
PPIB	DTNGSQFFITTVK	0.16	1457.74	0.00174	9.37	sp_P23284	2	1
HMGB2	RPPSAFFLFCSEHRPK	0.157	1976.01	0.00174	7.54	sp_P26583	2	1
STOM	LLAQTTLR	0.367	915.57	0.00759	5.63	sp_P27105	2	1
DPOD1	GLLPQILENLLSAR	0.0319	1536.92	0	7.65	sp_P28340	2	1
PRDX5	LLADPTGAFGKETDLLLDDSLVSIFGNRR	0.000032	3133.68	0	7.88	sp_P30044	2	1
GLYM	VLELVSITANKNTCPGDR	0.0969	1987.04	0.00056	8.57	sp_P34897	2	1
IF1AX	YNADEAR	0.333	838.37	0.00569	4.58	sp_P47813	2	1
CRIP2	KASGPPKGPSR	0.126	1081.62	0.0014	6.69	sp_P52943	2	1
TMM33	ALLANALTSALR	0.144	1213.73	0.00158	6.39	sp_P57088	2	1

RL26	HFNAPSHIR	0.0922	1078.56	0.00047	5.98	sp_P61254	2	1
TBA1B	IHFPLATYAPVISAEK	0.0749	1756.98	0	7.36	sp_P68363	2	1
SOS2	KILDEAVELSQDHFKK	0.406	1900.02	0.00959	2	sp_Q07890	2	1
CPSF1	LGNSLLLK	0.173	871.57	0.00225	7.17	sp_Q10570	2	1
CBX3	CPQIVIAFYEER	0.117	1524.77	0.00056	14.84	sp_Q13185	2	1
PAP1L	YQGVNLYVK	0.291	1083.59	0.00452	6.77	sp_Q4VXU2	2	1
P4R3C	EVIPITNSELR	0.391	1270.71	0.00898	5.31	sp_Q6ZMV5	2	1
H32	LVREIAQDFKTDLR	0.175	1703.96	0.00225	7.67	sp_Q71DI3	2	1
PK1L2	LAEVLR	0.252	700.44	0.00359	6.81	sp_Q7Z442	2	1
NRAP	AGEILSEKK	0.366	1016.61	0.00754	7.46	sp_Q86VF7	2	1
ANKH1	KLLDEGR	0.125	830.48	0.0012	5.64	sp_Q8IWZ3	2	1
PACE1	TPEFLR	0.337	762.42	0.00637	6.24	sp_Q8IZE3	2	1
PRS33	LGSTSPR	0.352	717.39	0.00698	3.34	sp_Q8NF86	2	1
PTPM1	VLFYPTLLYTLFR	0.014	1645.95	0	11.33	sp_Q8WUK0	2	1
GOGA1	AINTLETR	0.34	917.51	0.00658	6.93	sp_Q92805	2	1
TBD2A	KLEHLK	0.333	767.48	0.00569	5.76	sp_Q9BYX2	2	1
T126A	SGLTGLVIGGLYPVFLAIPVNGGLAAR	0.00393	2625.54	0	10.5	sp_Q9H061	2	1
GHC2	GAAVNLTLVTPEK	0.186	1312.75	0.00261	5.73	sp_Q9H1K4	2	1
SPTN4	ALAVEGKR	0.183	843.51	0.00244	4.48	sp_Q9H254	2	1
BAZ1A	LSSSFSSR	0.163	870.44	0.00174	7.58	sp_Q9NRL2	2	1
TBA8	TIQFVDWCPTGFK	0.0387	1598.78	0	8.23	sp_Q9NY65	2	1
RB22A	QNEIHK	0.268	768.4	0.0041	5.47	sp_Q9UL26	2	1
NOL7	VNIQKK	0.261	729.47	0.00389	4.6	sp_Q9UMY1	2	1
PAL4A	IIPGFMCQGGDFTR	0.38	1655.78	0.00854	7.48	sp_Q9Y536	2	1
MTCH2	VLIQVGYEPLPPTIGR	0.196	1752.02	0.00261	8.52	sp_Q9Y6C9	2	1
