# Supplementary material for: MCM7 promotes liver fibrosis by transcriptionally regulating IL11 via the SHCBP1-RACGAP1-STAT3 axis
Source: Cell Death Dis. 2025 Aug 11;16(1):608. doi: 10.1038/s41419-025-07937-x (PMC12339988; doi:10.1038/s41419-025-07937-x)

The original protein Western blot images for Figure 1, The red dashed boxes indicate the protein bands that are referenced in the main text.

Fig. 1F

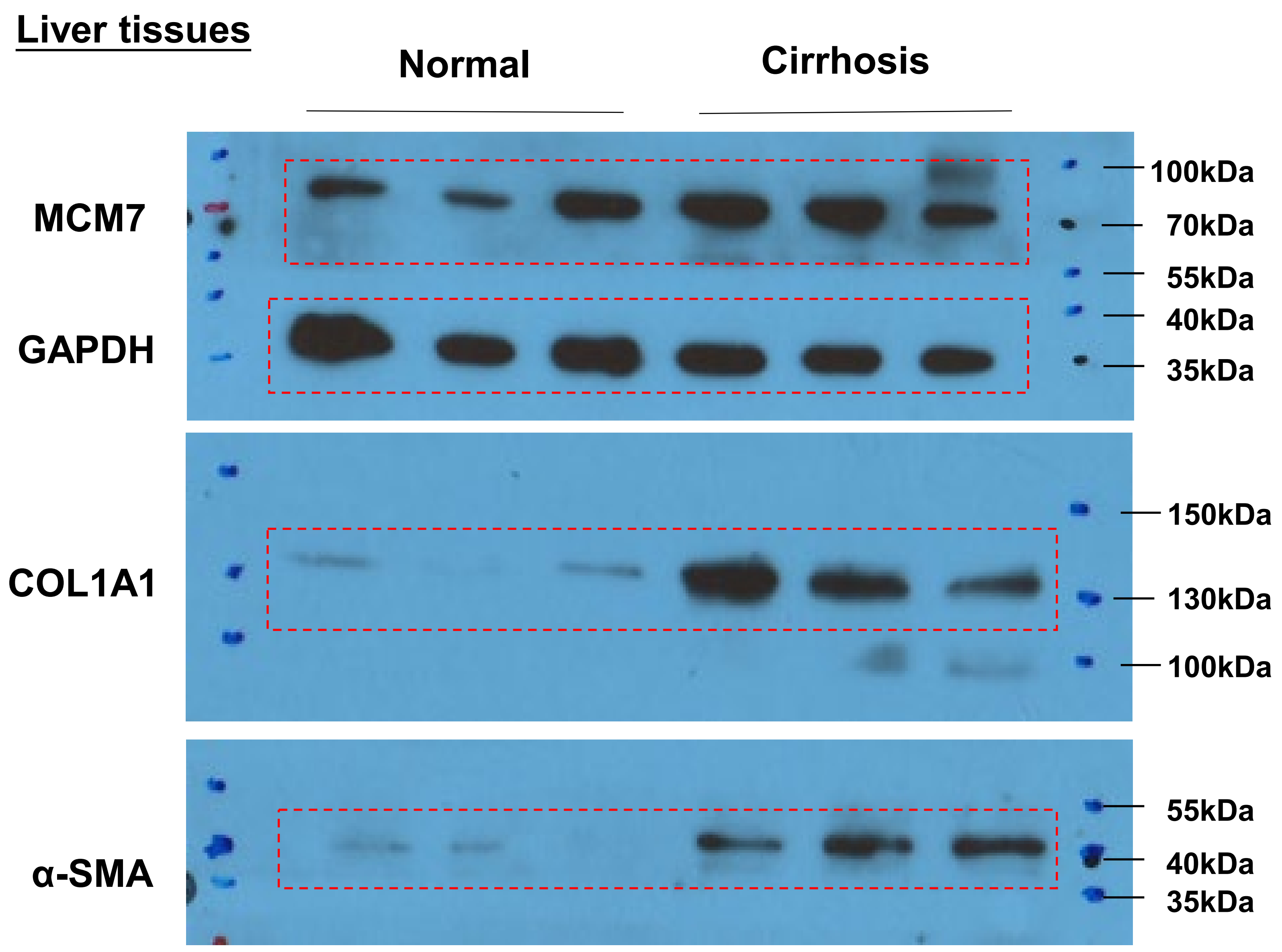

Fig. 1I

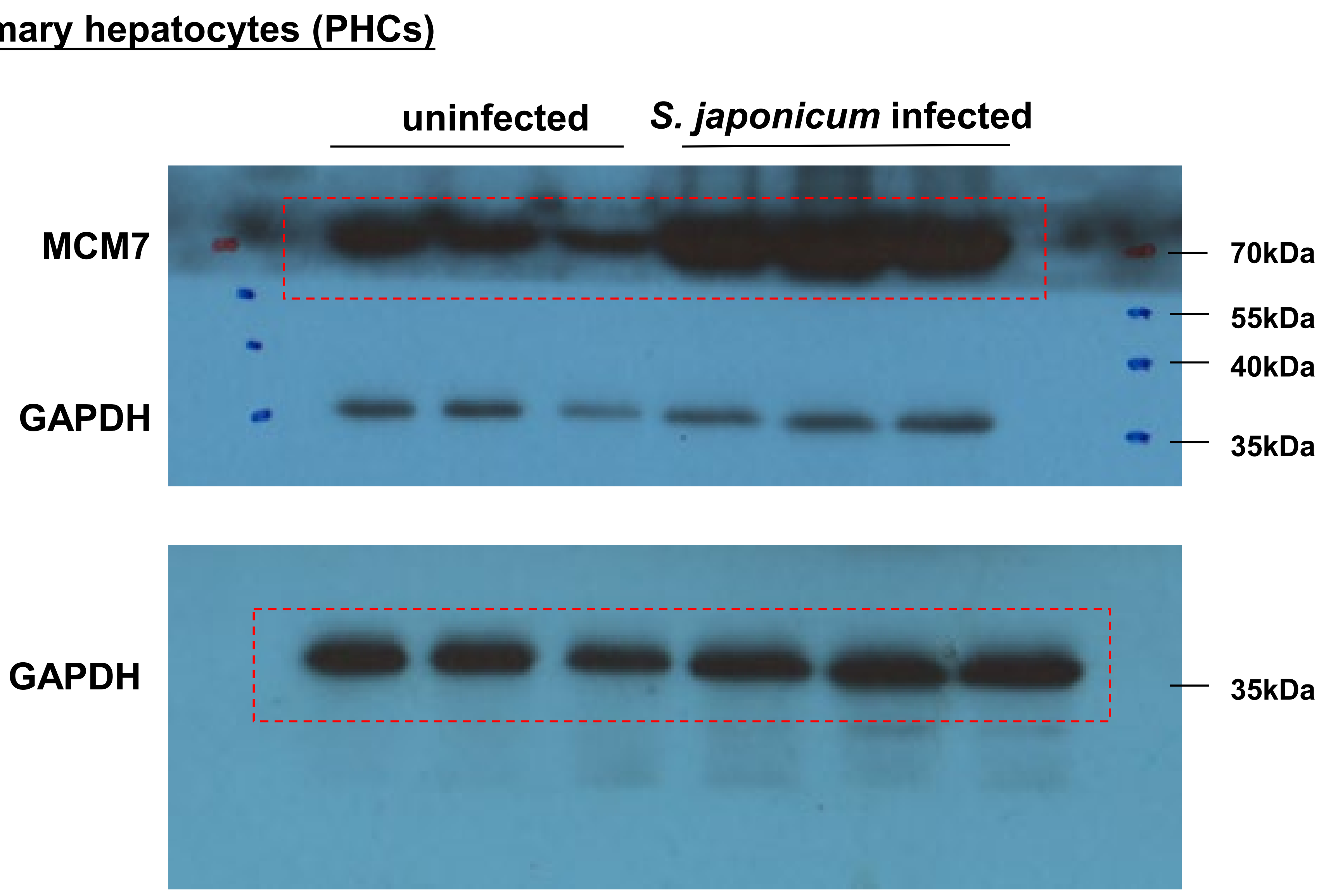

Fig. 1K

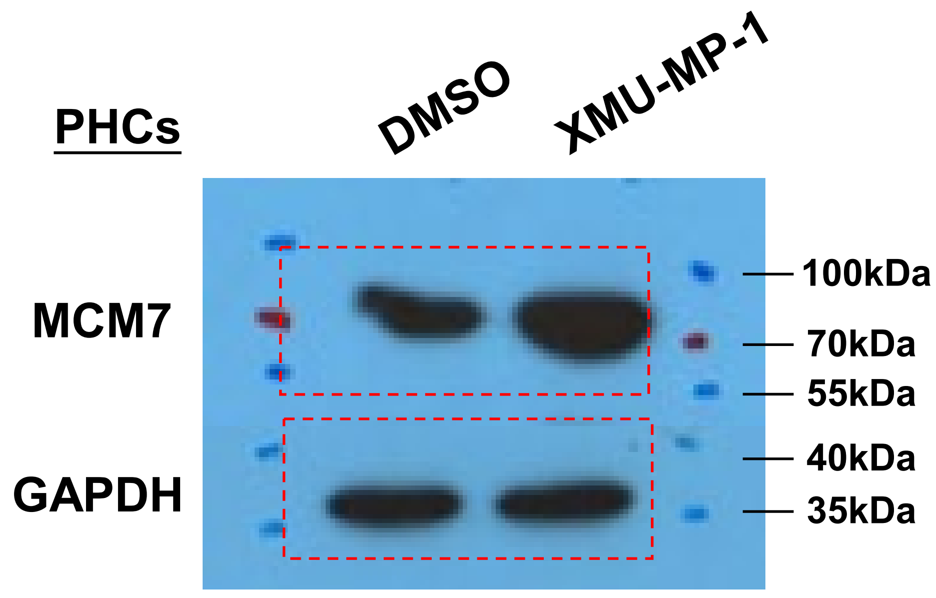

The original protein Western blot images for Figure 1, The red dashed boxes indicate the protein bands that are referenced in the main text.

Fig. 1M

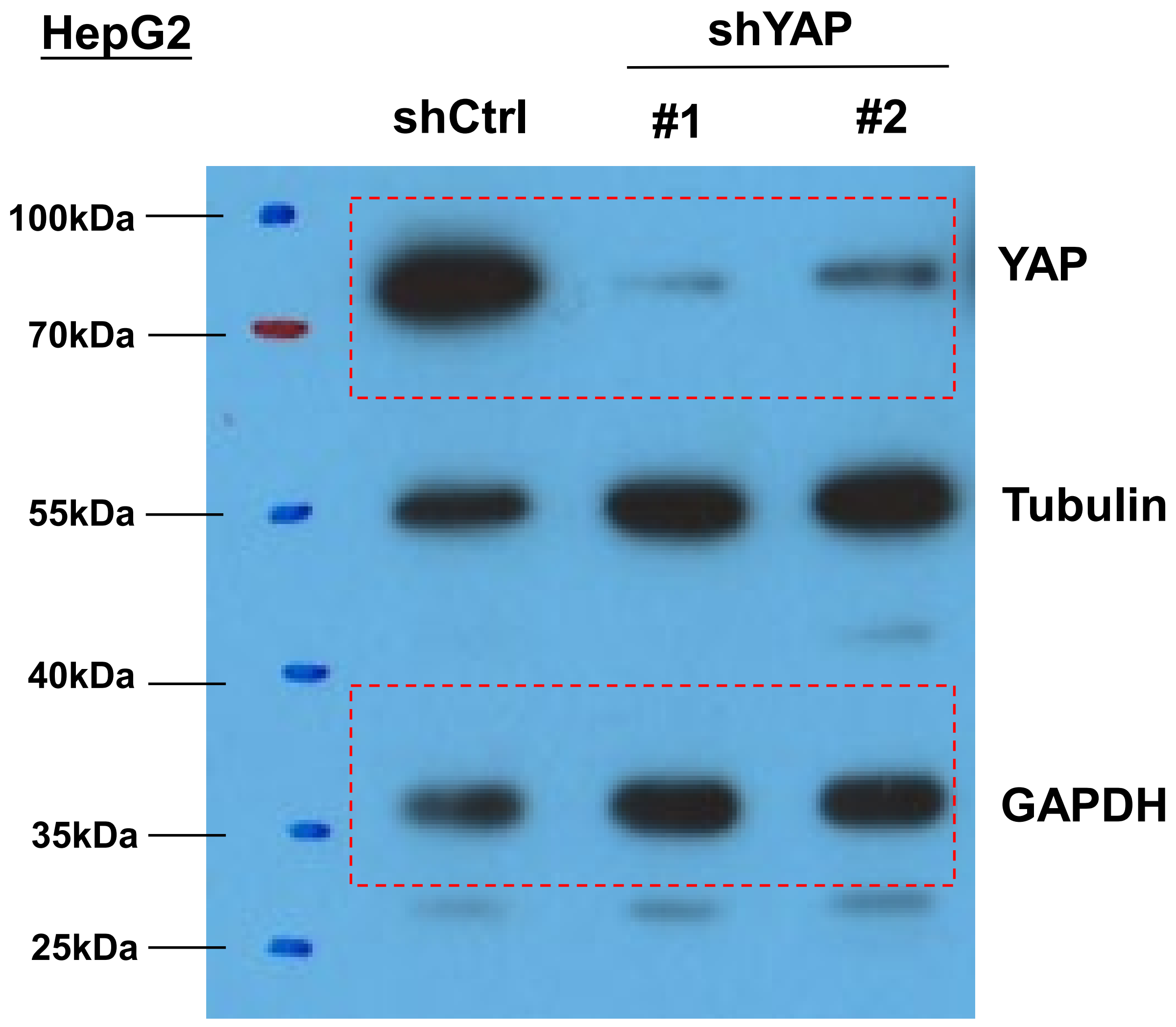

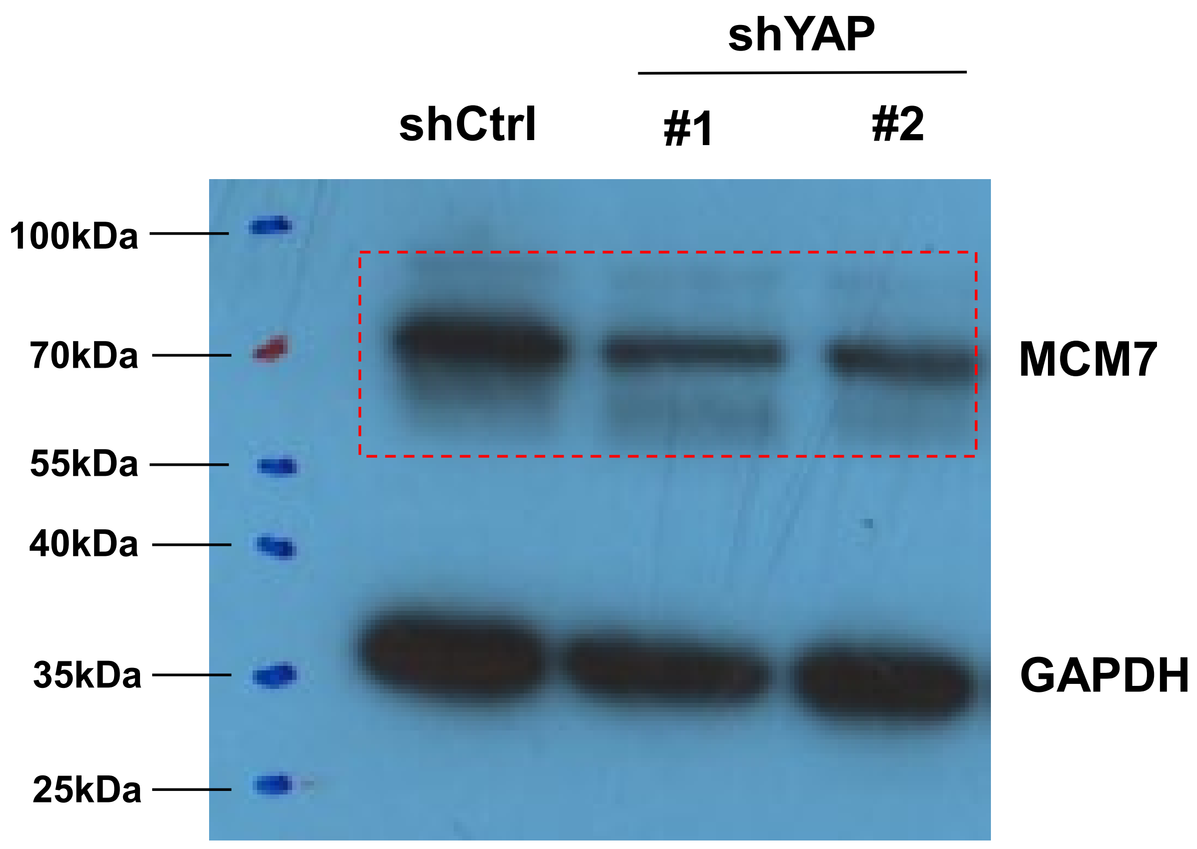

The original protein Western blot images for Figure 2 and 3, The red dashed boxes indicate the protein bands that are referenced in the main text.

Fig. 2F

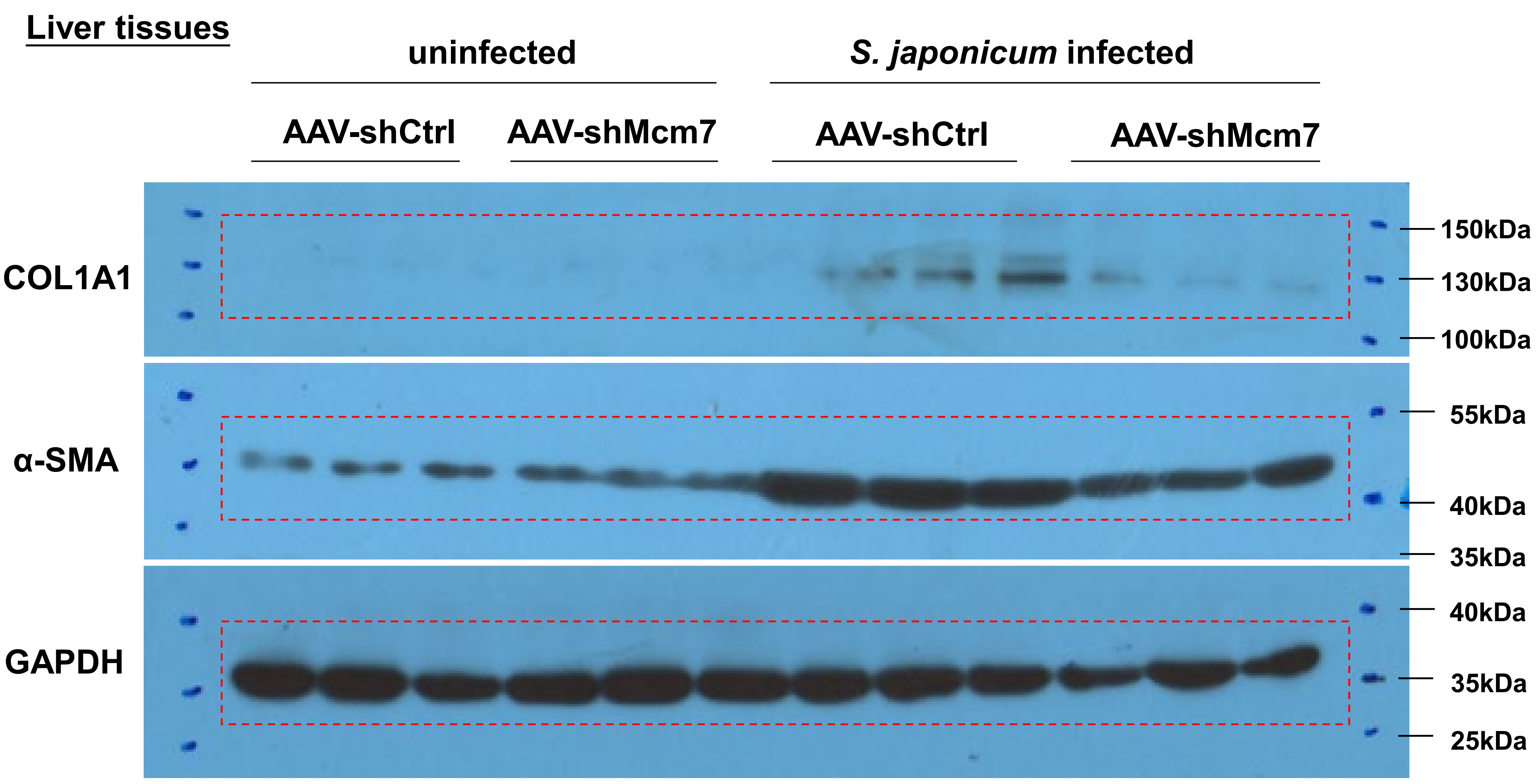

Fig. 3E

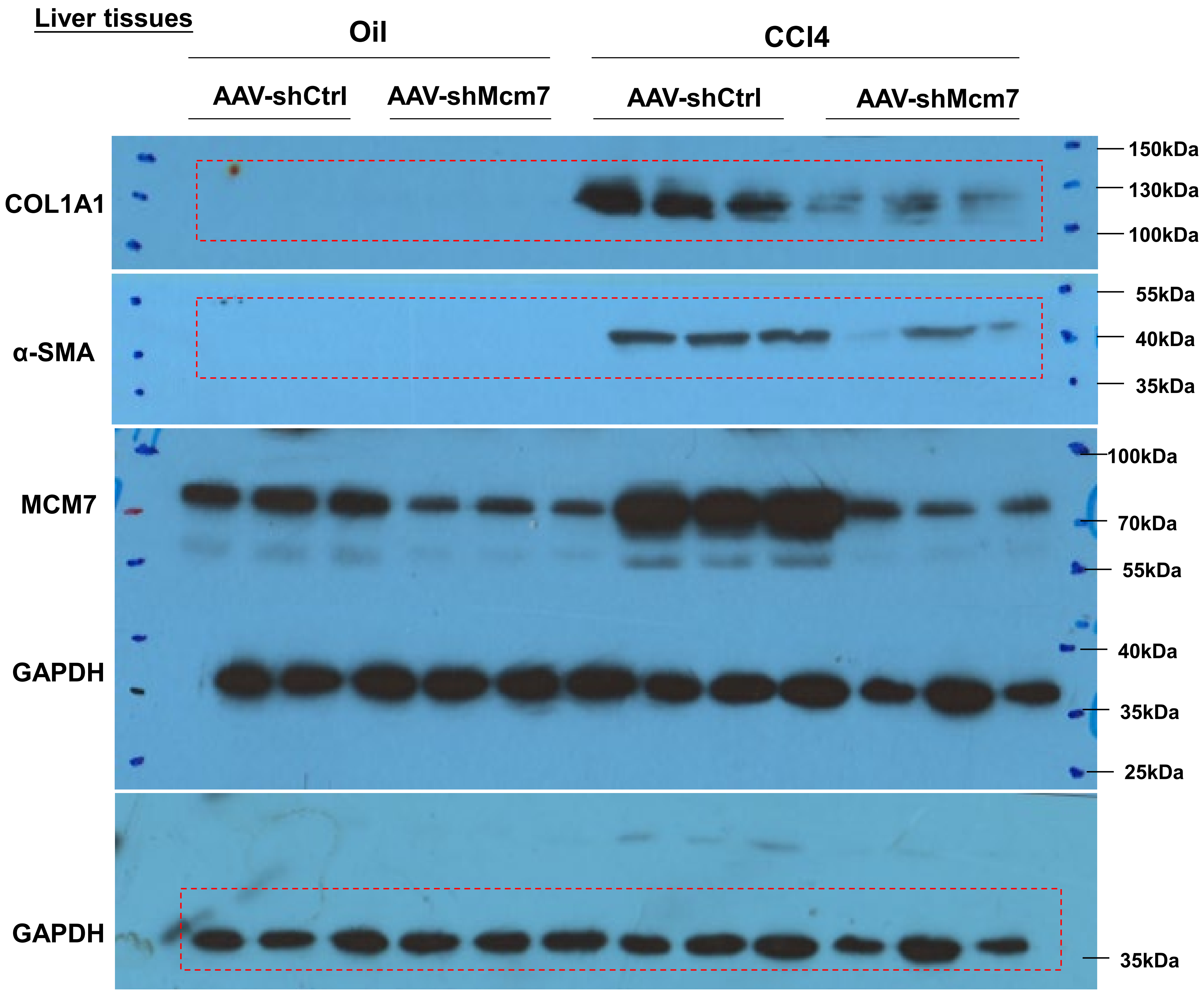

The original protein Western blot images for Figure 4，The red dashed boxes indicate the protein bands that are referenced in the main text.

Fig. 4D

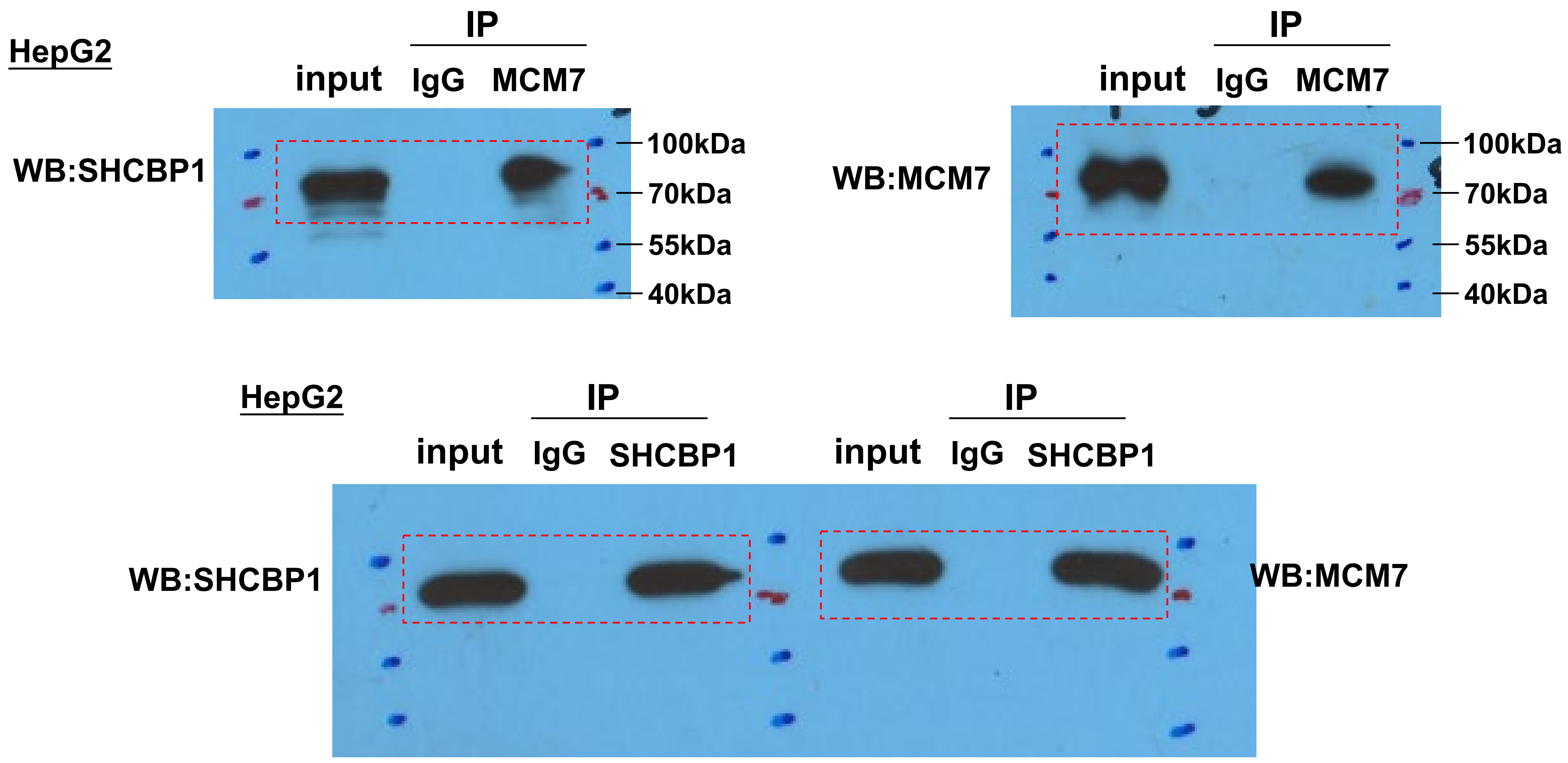

Fig. 4E

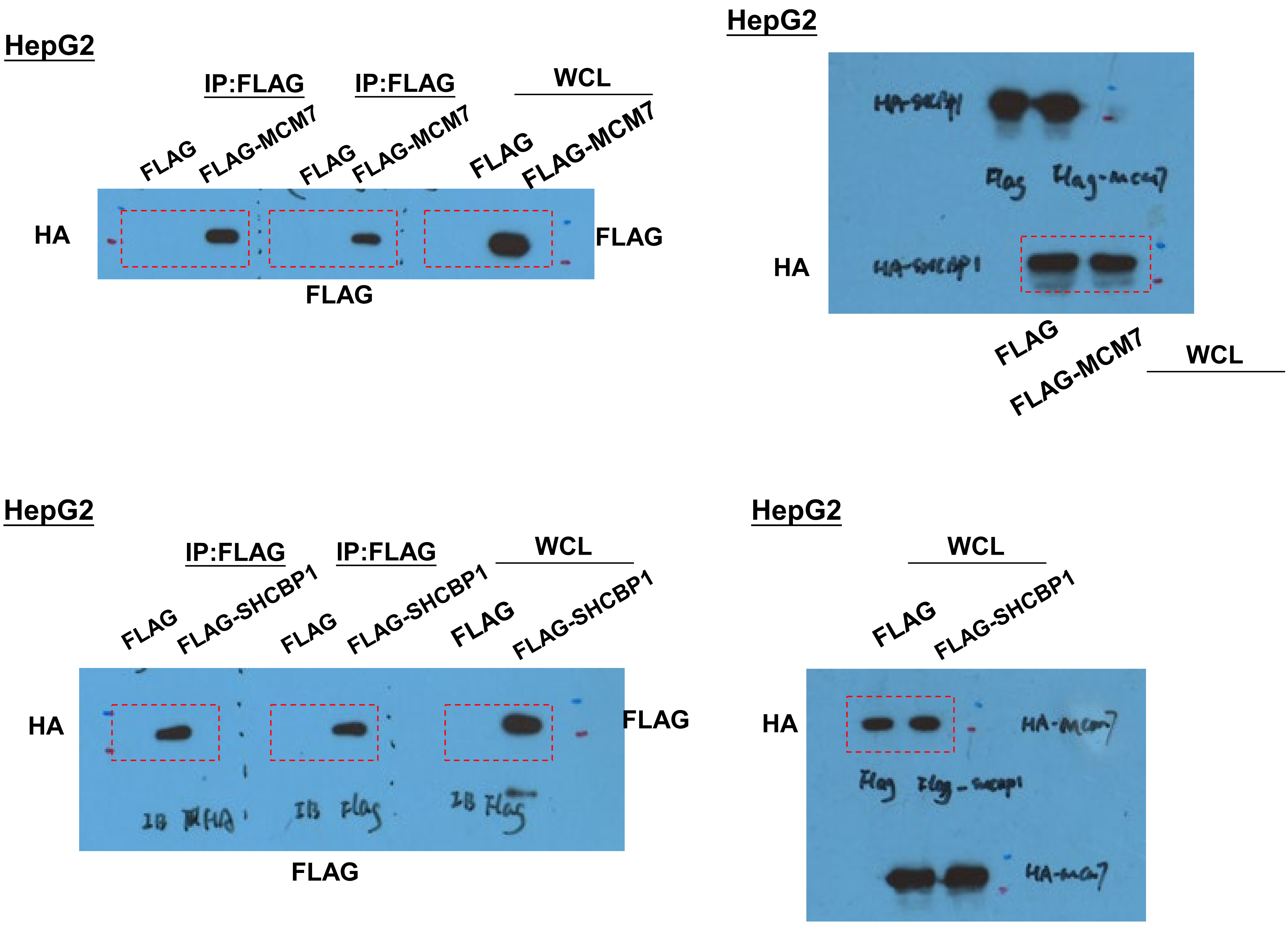

The original protein Western blot images for Figure 4, The red dashed boxes indicate the protein bands that are referenced in the main text.

Fig. 4G

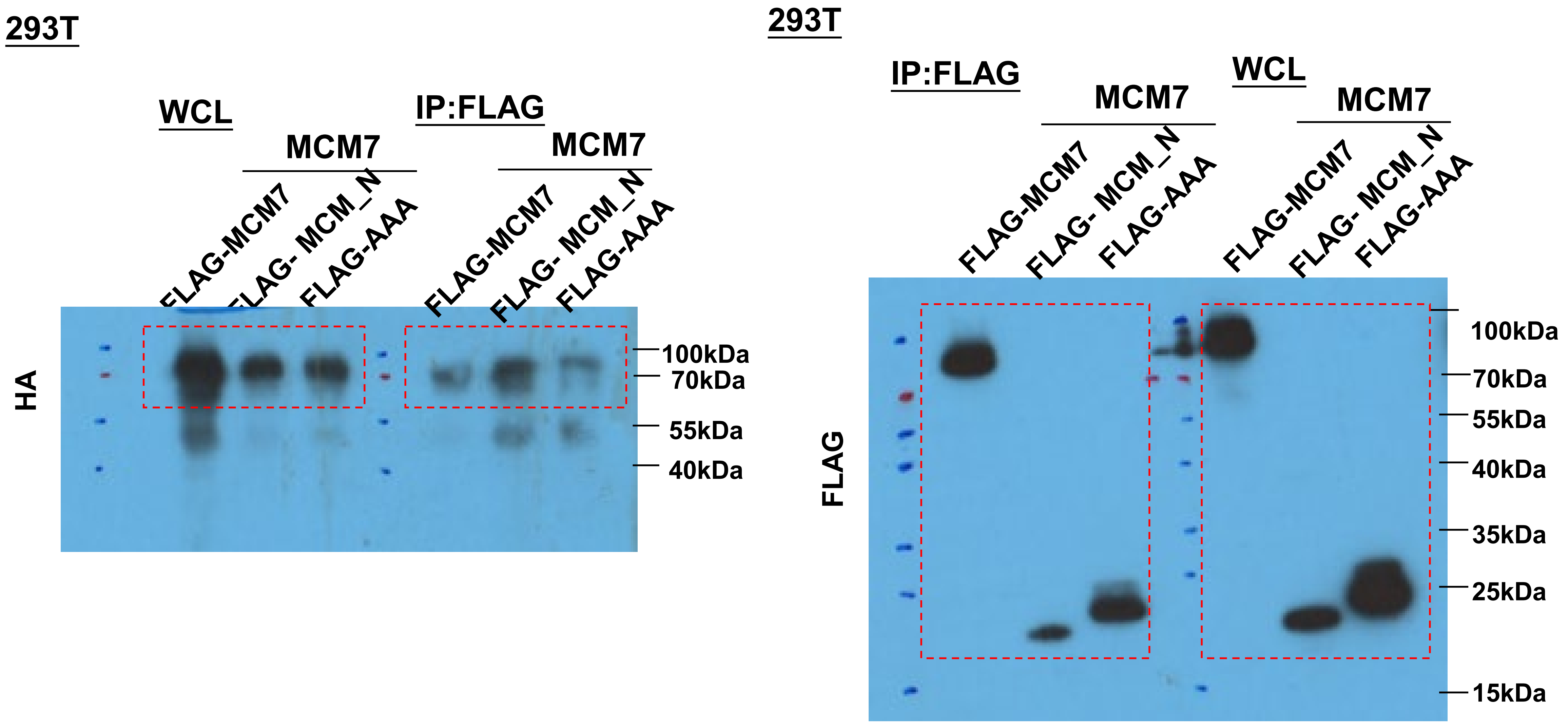

Fig. 4G

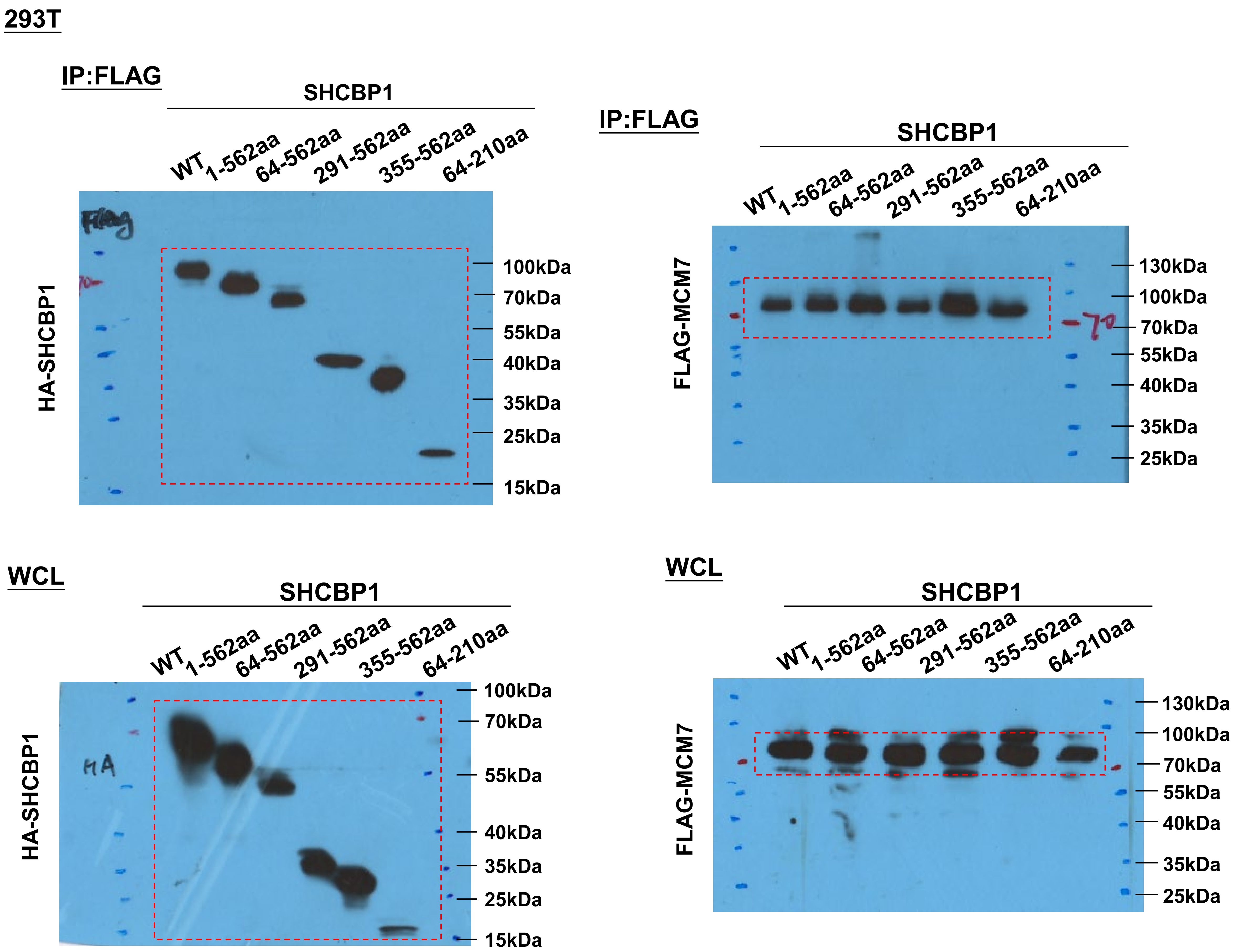

The original protein Western blot images for Figure 5, The red dashed boxes indicate the protein bands that are referenced in the main text.

Fig. 5F

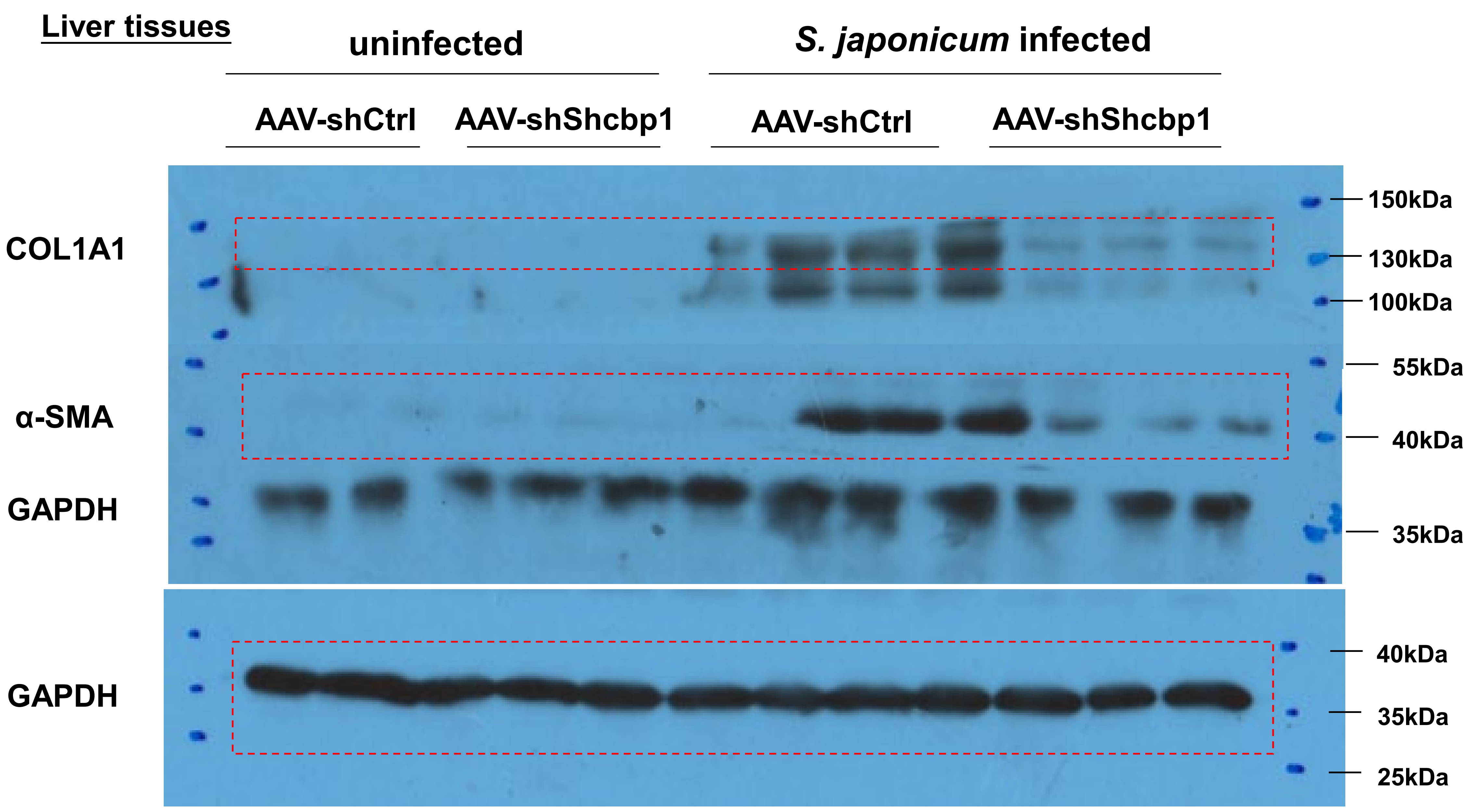

The original protein Western blot images for Figure 7, The red dashed boxes indicate the protein bands that are referenced in the main text.

Fig. 7C

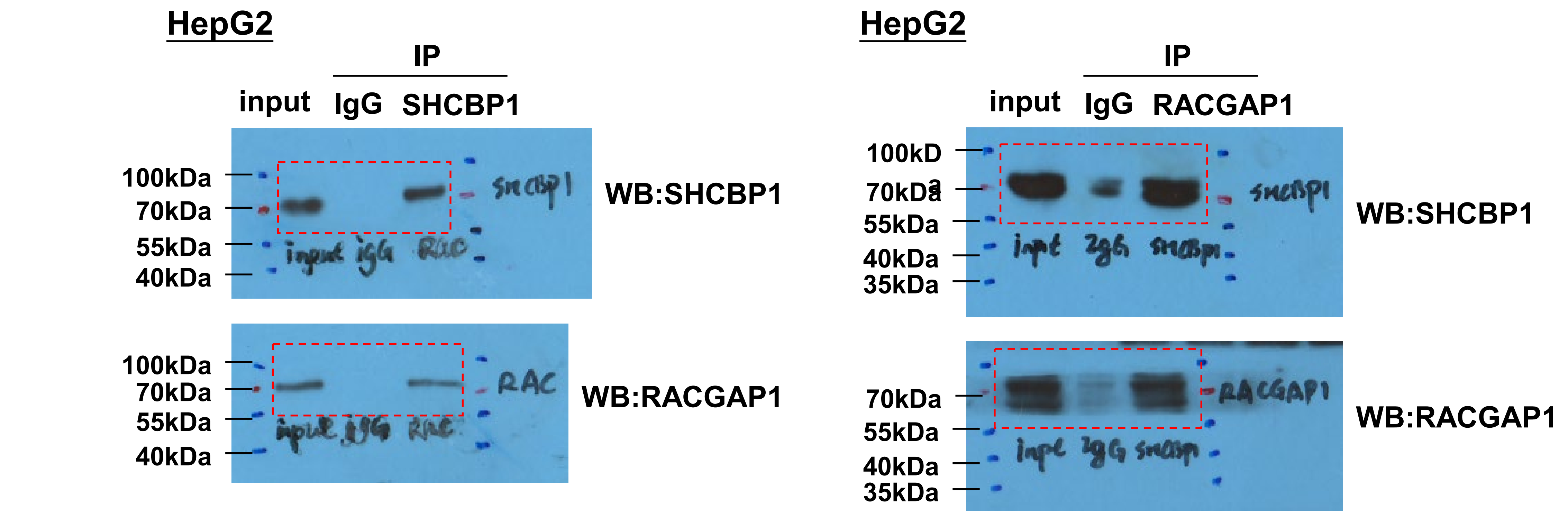

Fig. 7D

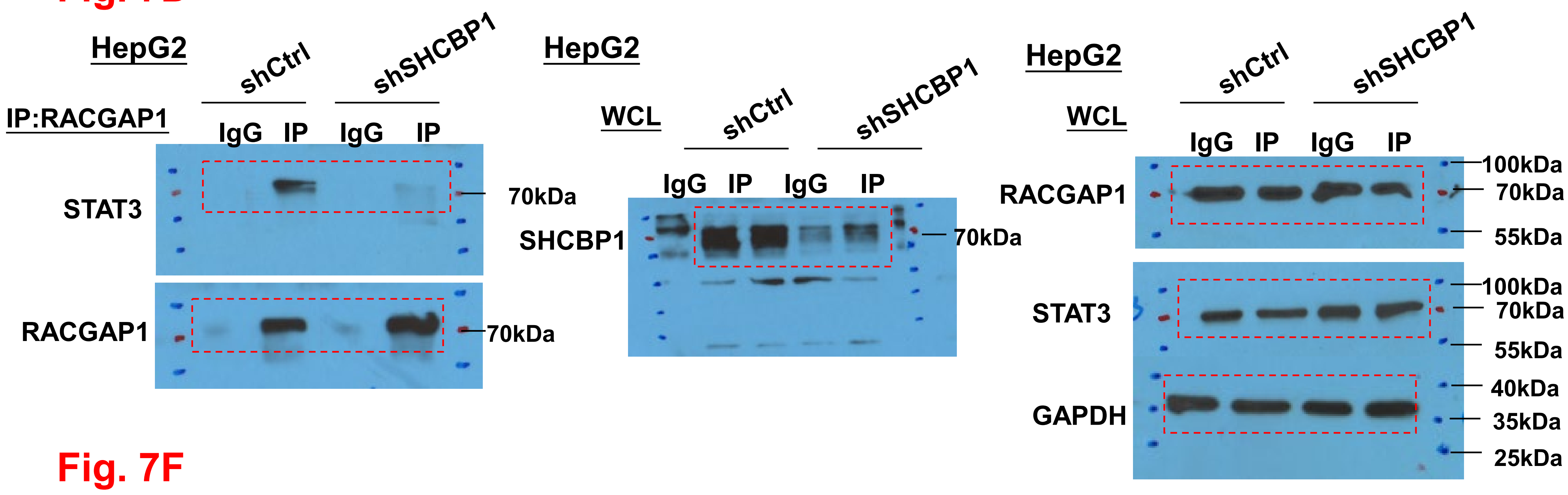

Fig. 7F

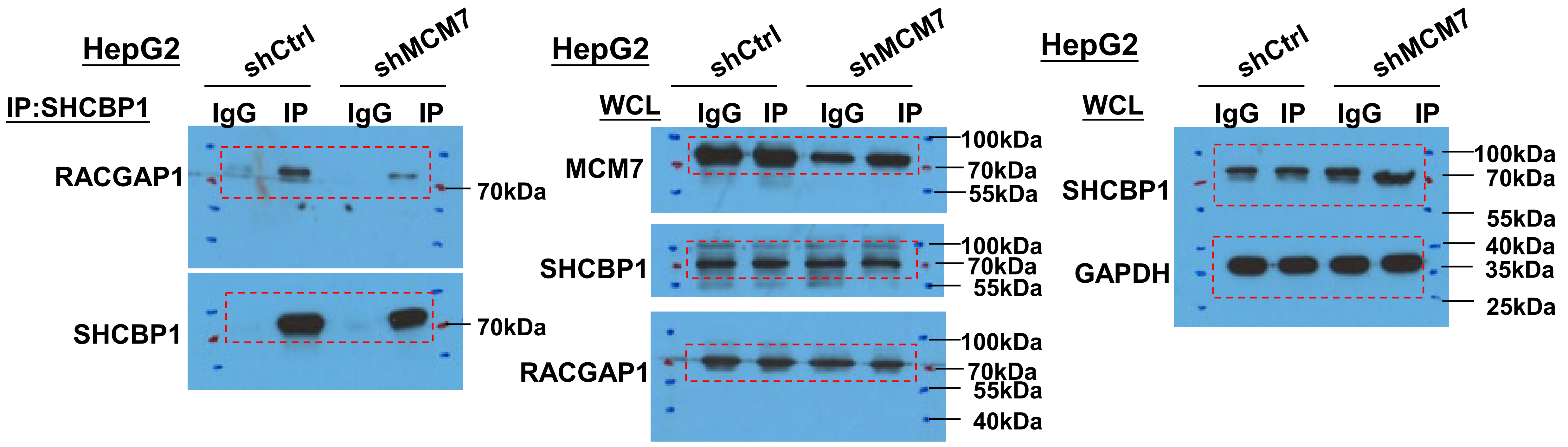

Fig. 7G

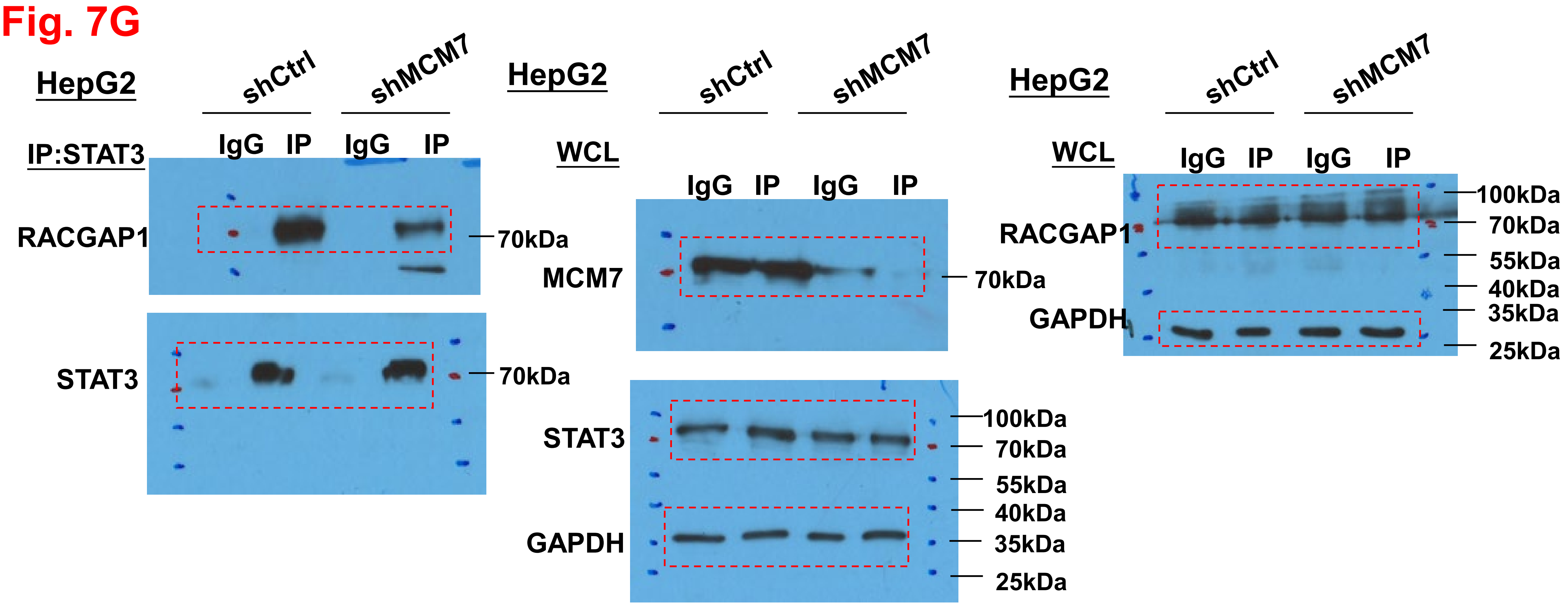

The original protein Western blot images for Figure 7, The red dashed boxes indicate the protein bands that are referenced in the main text.

Fig. 7I

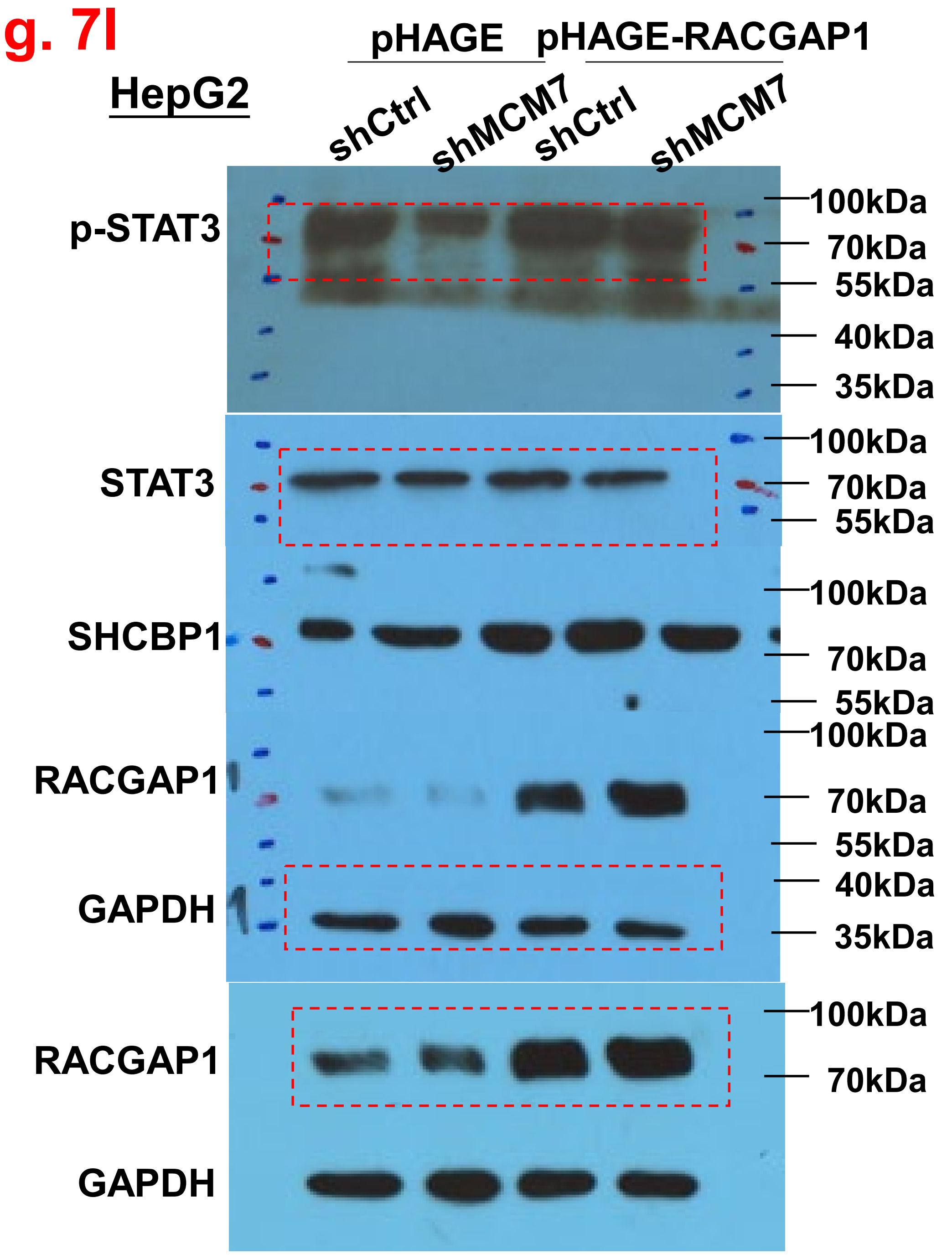

Fig. 7J

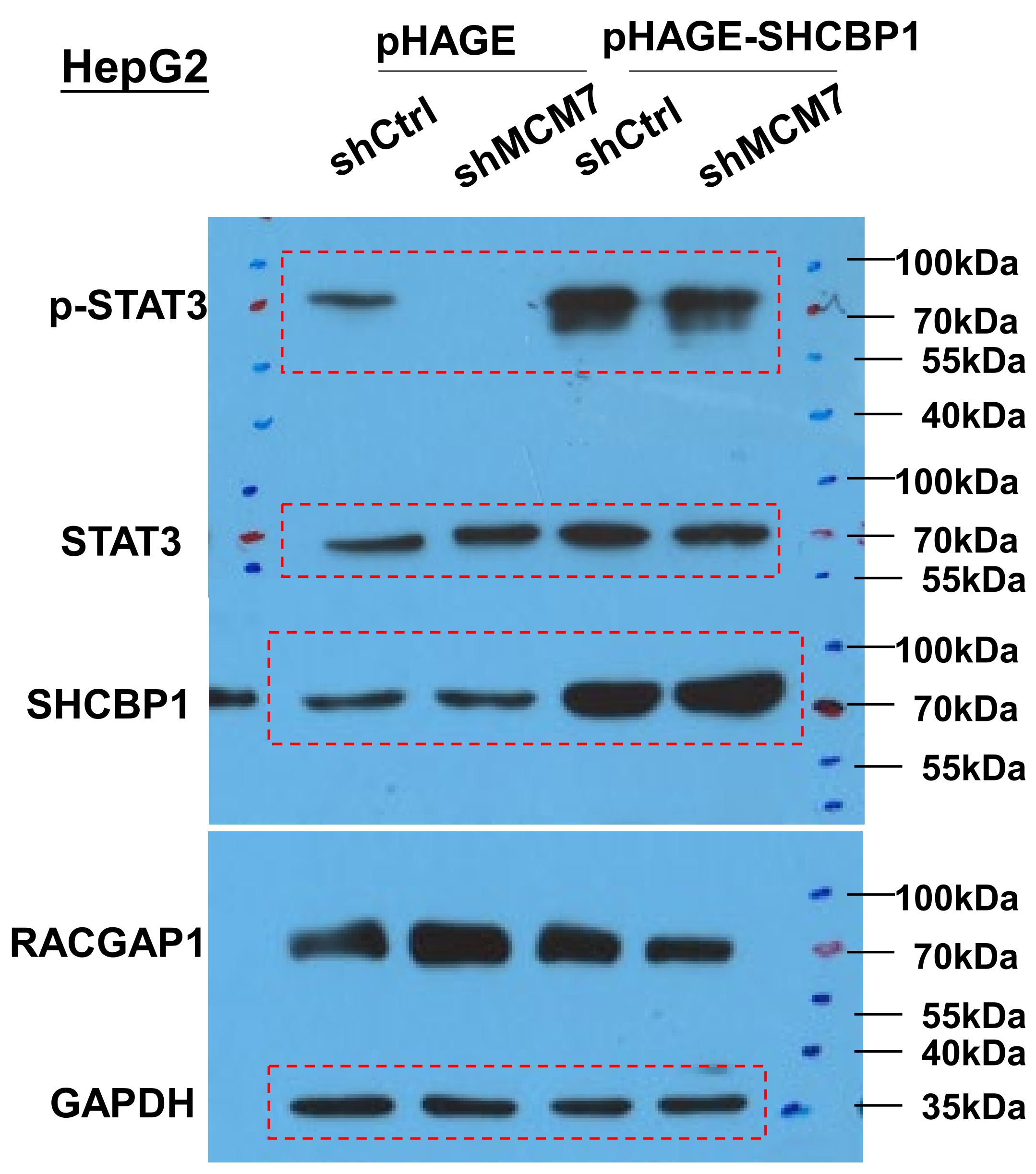

Fig. 7K

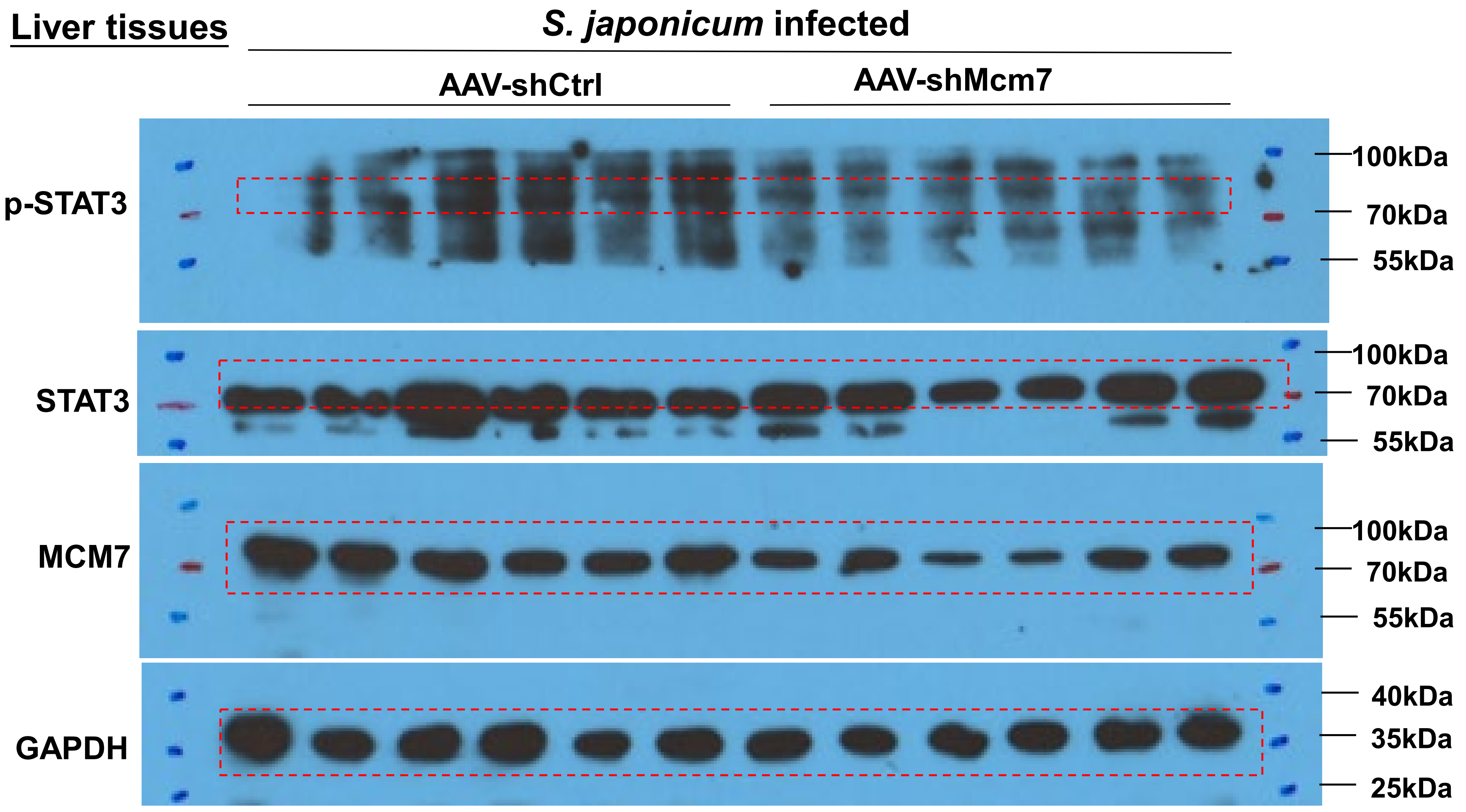

Fig.7M

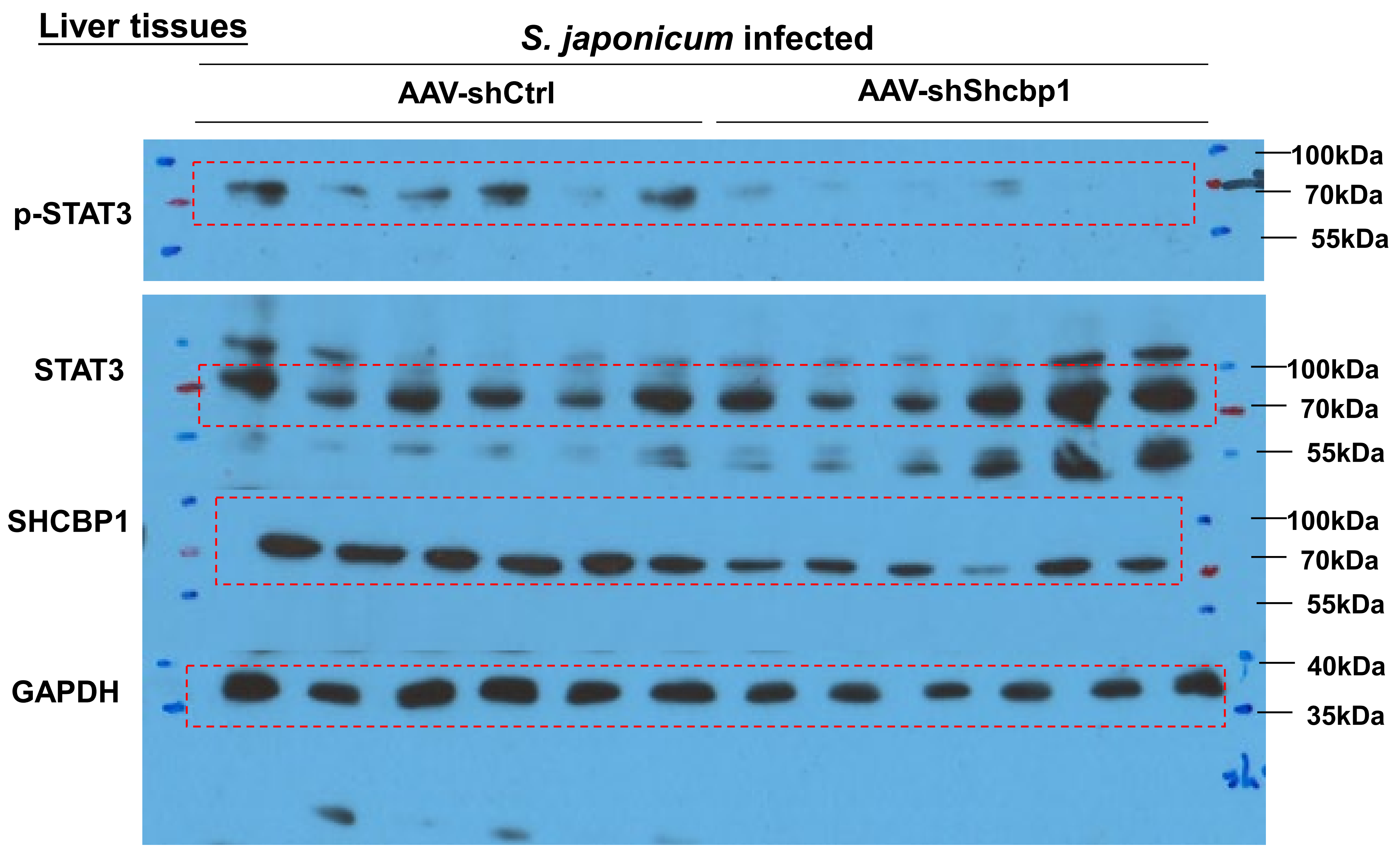

The original protein Western blot images for Figure 8, The red dashed boxes indicate the protein bands that are referenced in the main text.

Fig. 8H

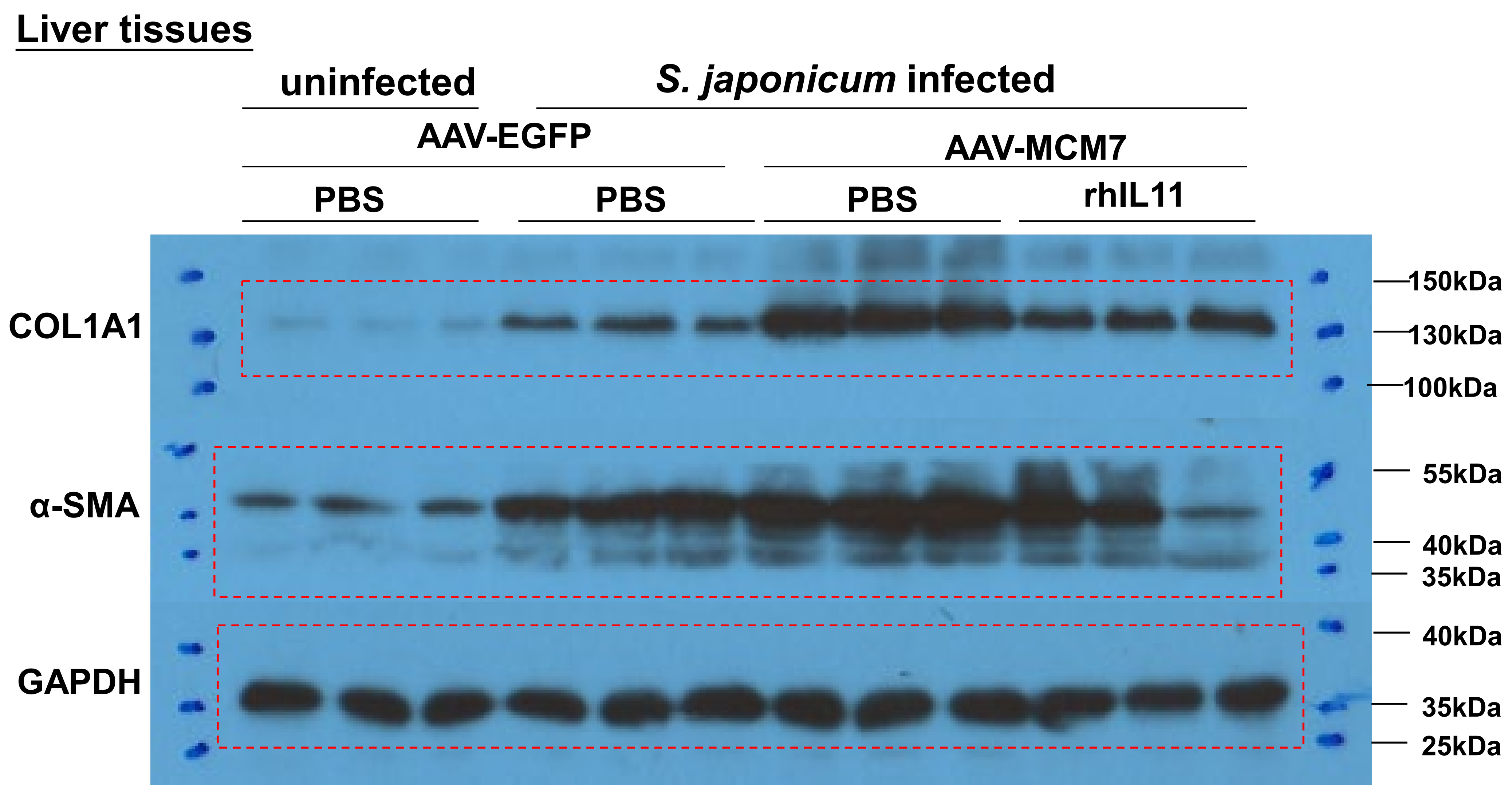

The original protein Western blot images for Figure S1， The red dashed boxes indicate the protein bands that are referenced in the main text.

Fig. S1E

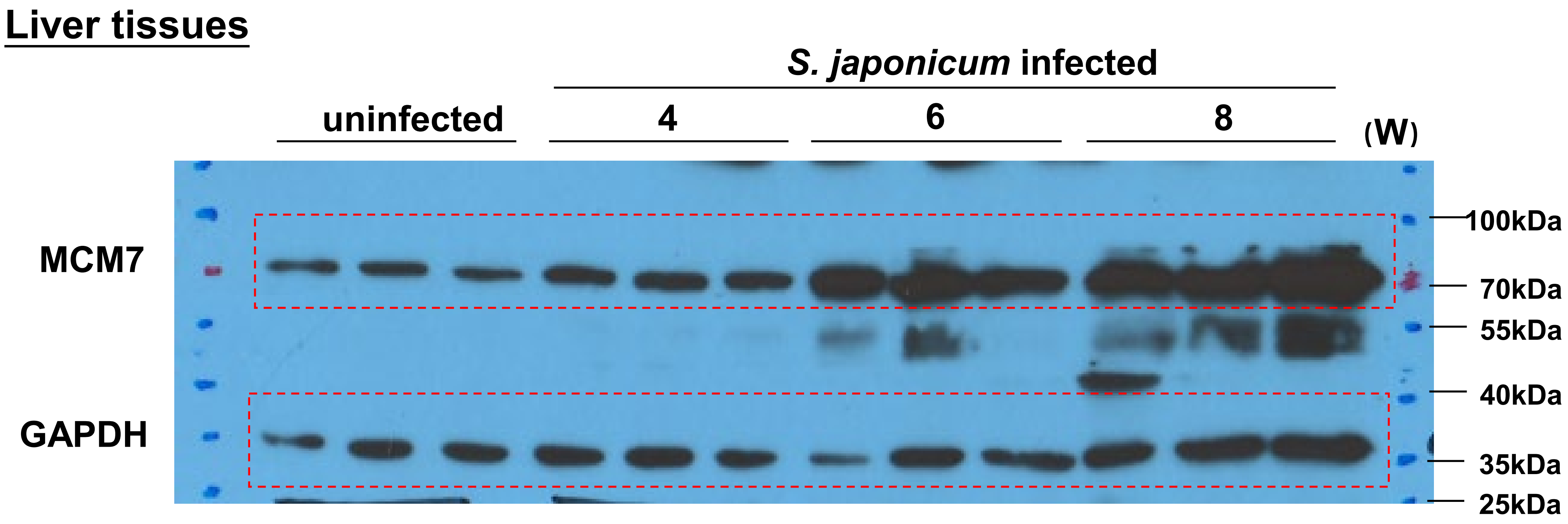

Fig. S1G

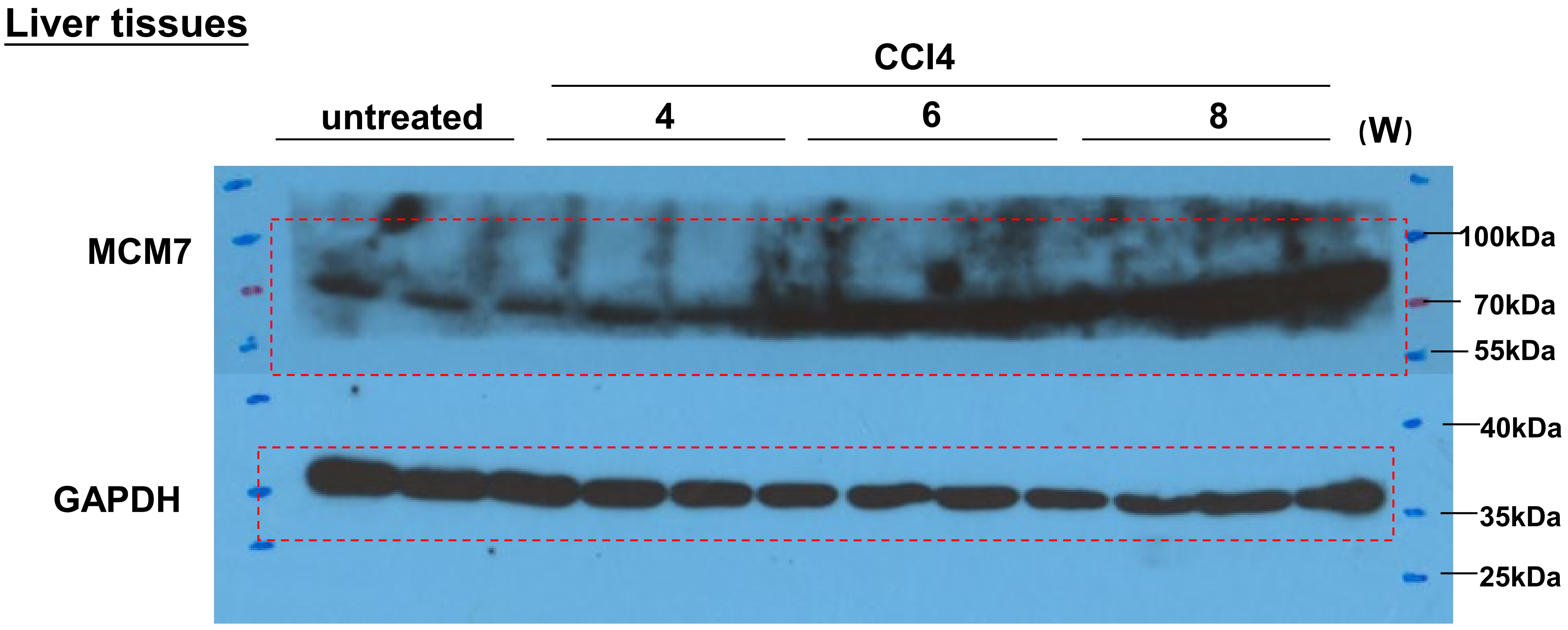

Fig. S1K

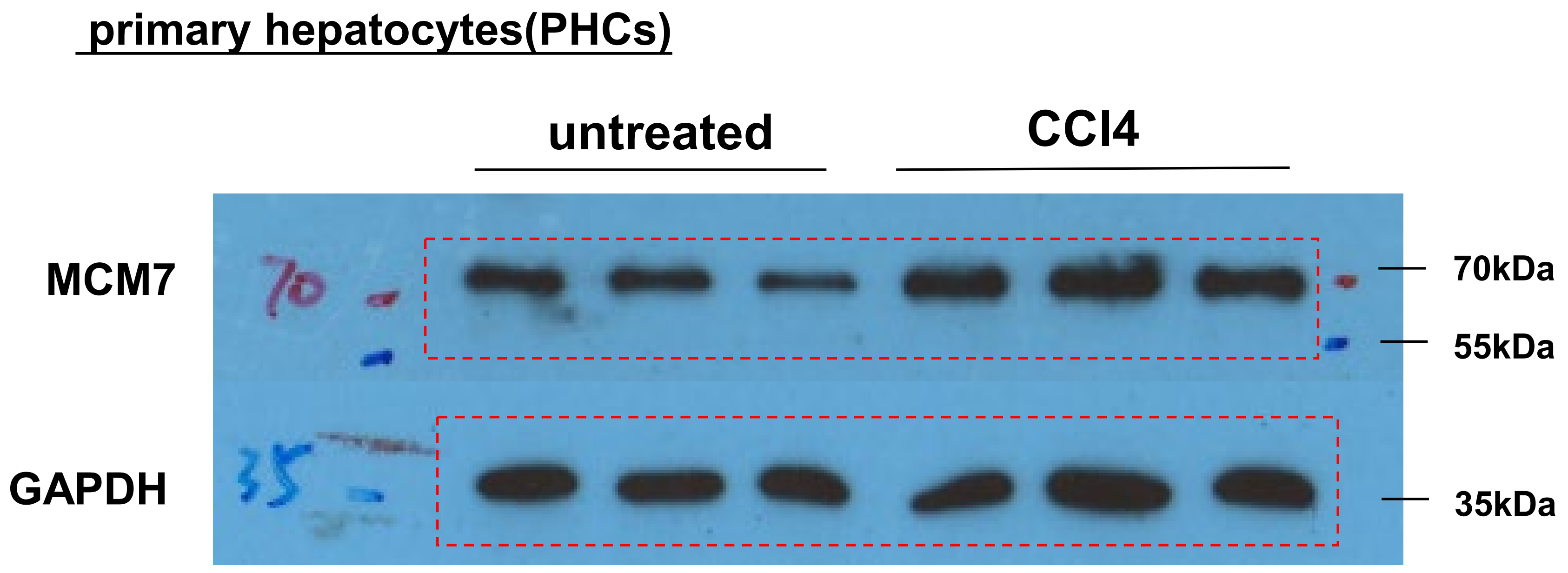

The original protein Western blot images for Figure S2, The red dashed boxes indicate the protein bands that are referenced in the main text.

Fig. S2A

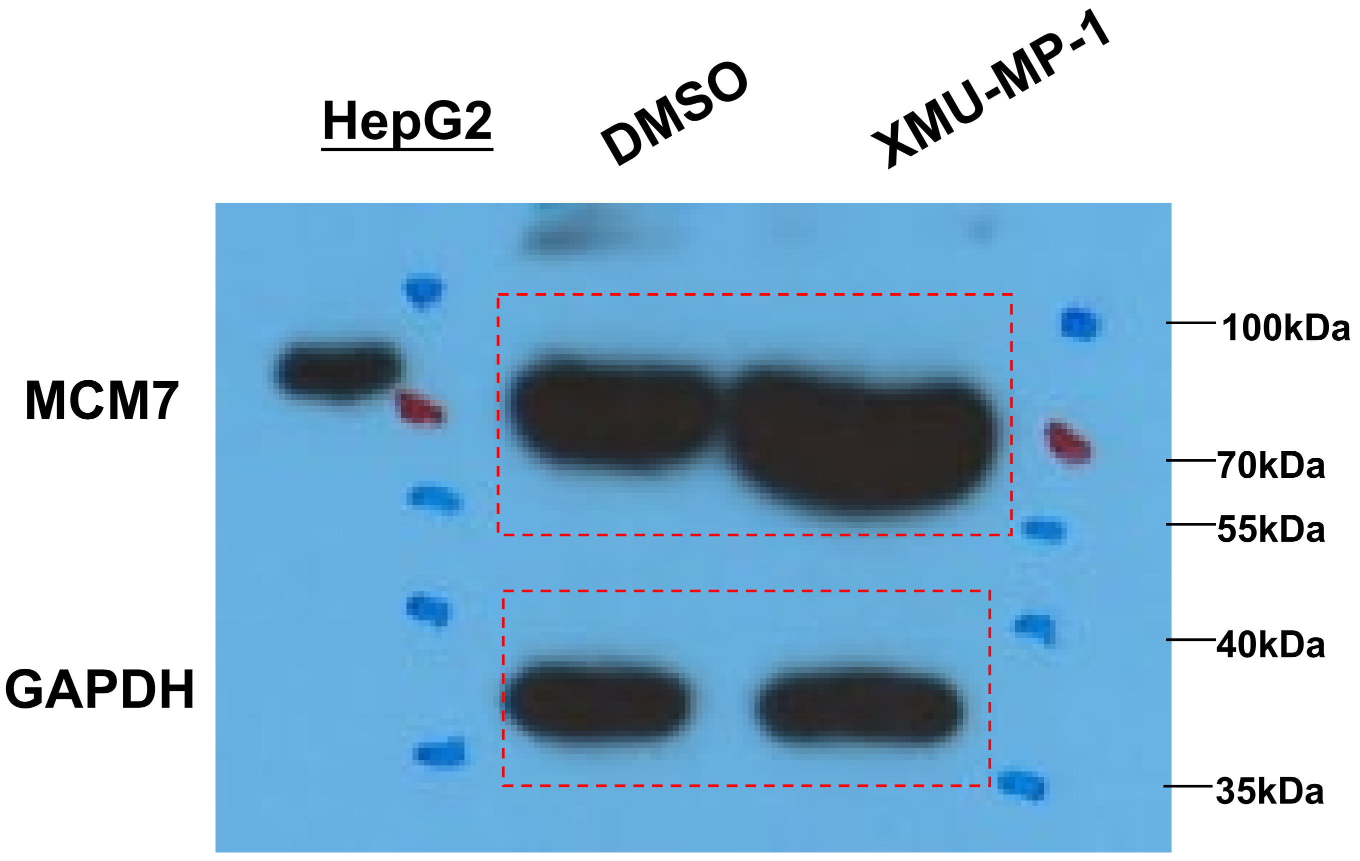

Fig. S2C

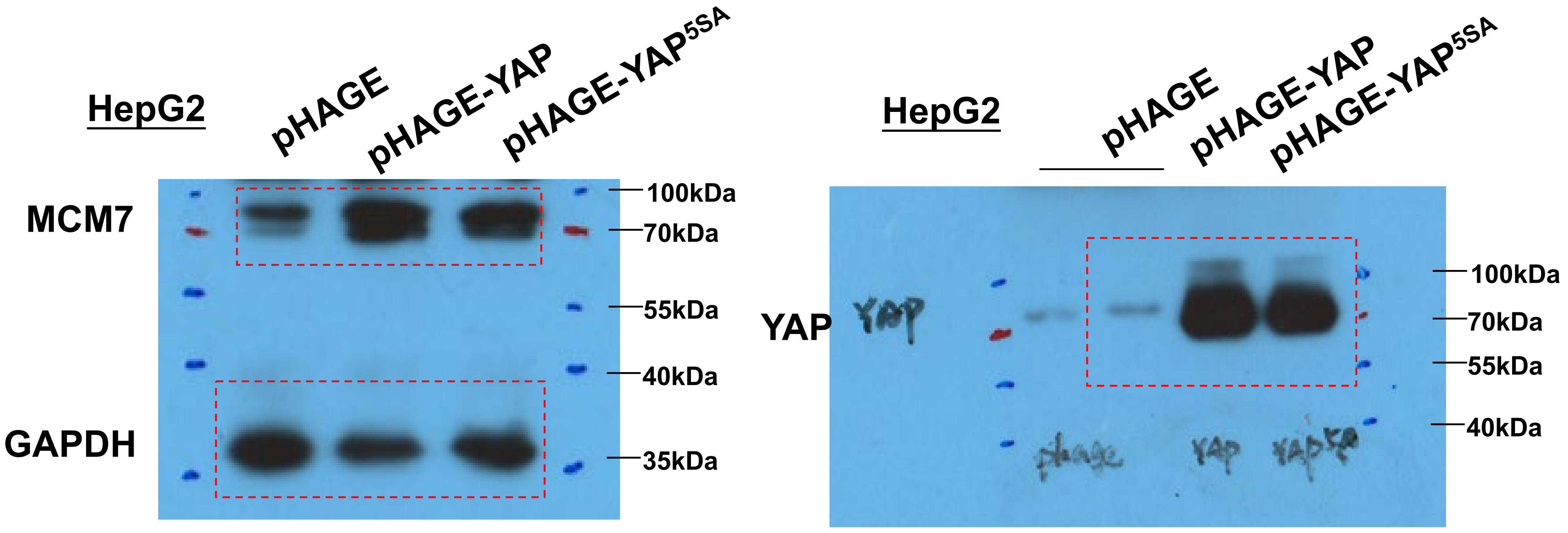

The original protein Western blot images for Figure S3, The red dashed boxes indicate the protein bands that are referenced in the main text.

Fig. S3B

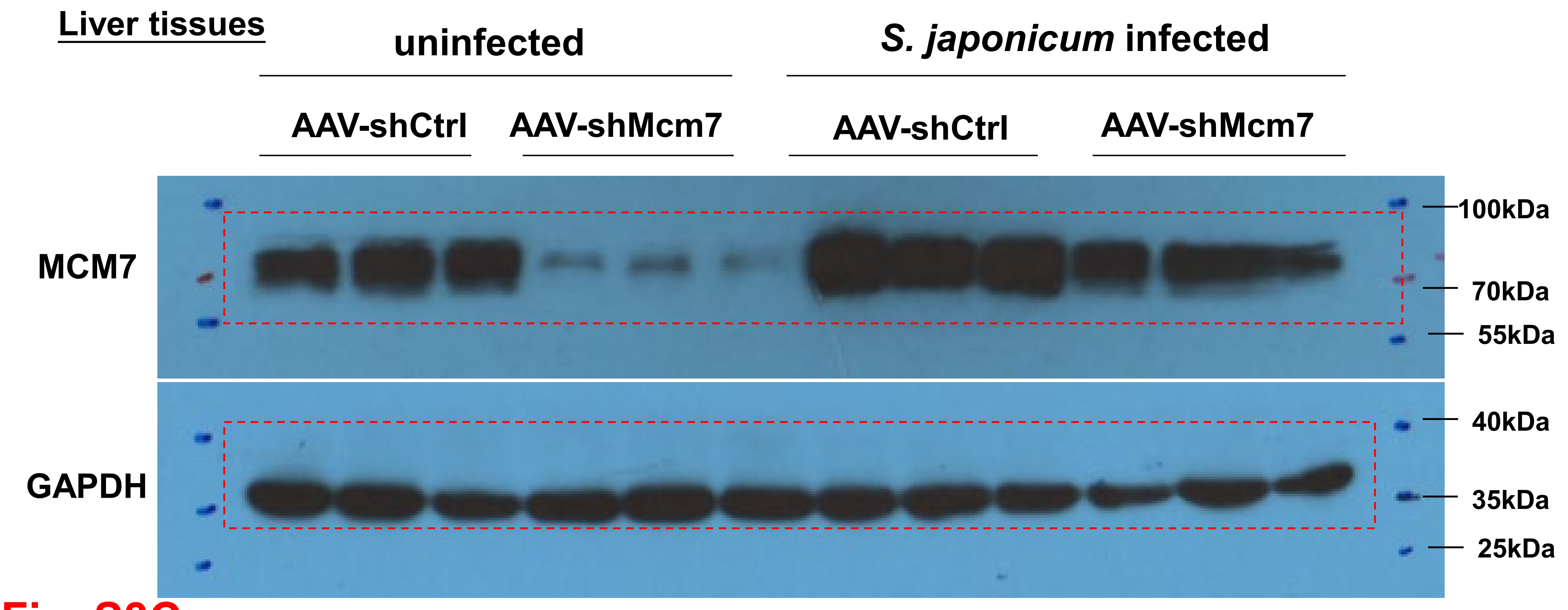

Fig. S3C

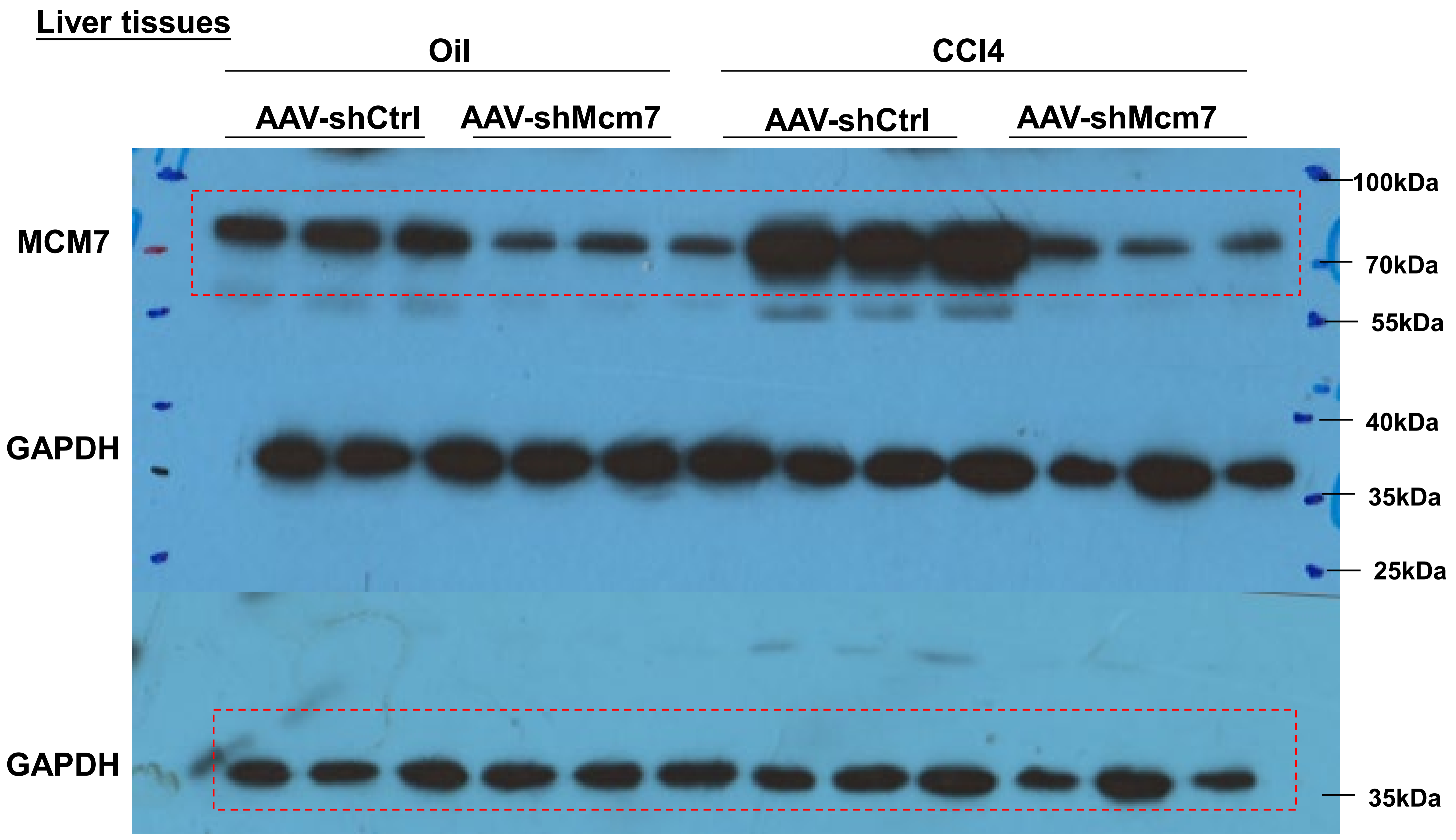

Fig. S3D

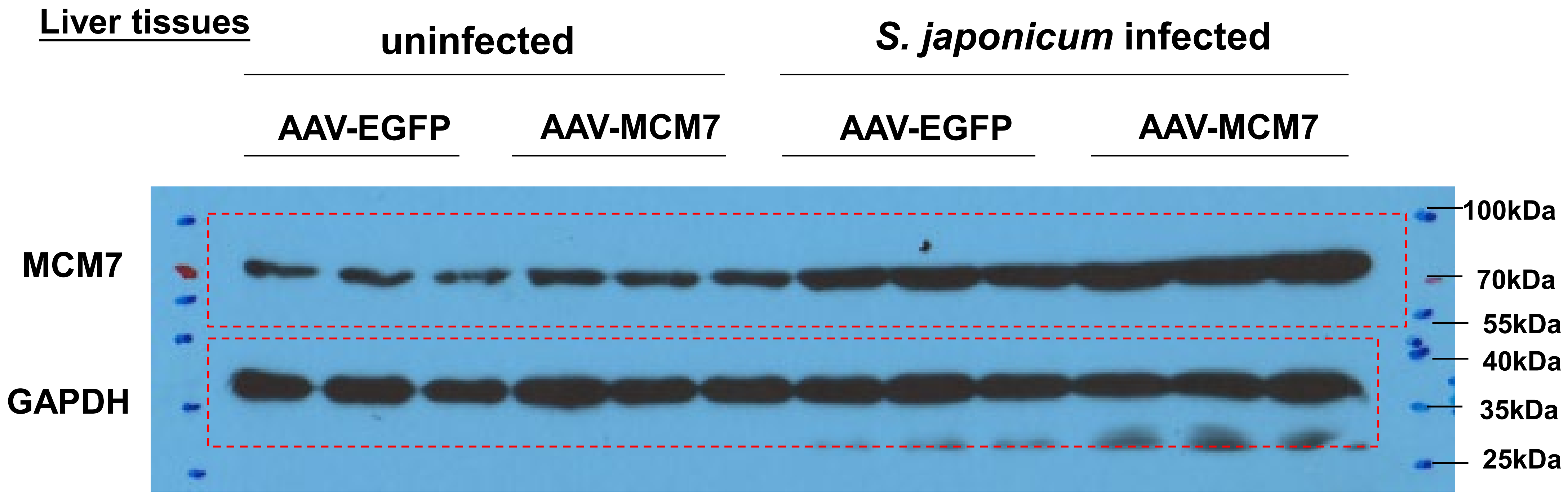

Fig. S3E

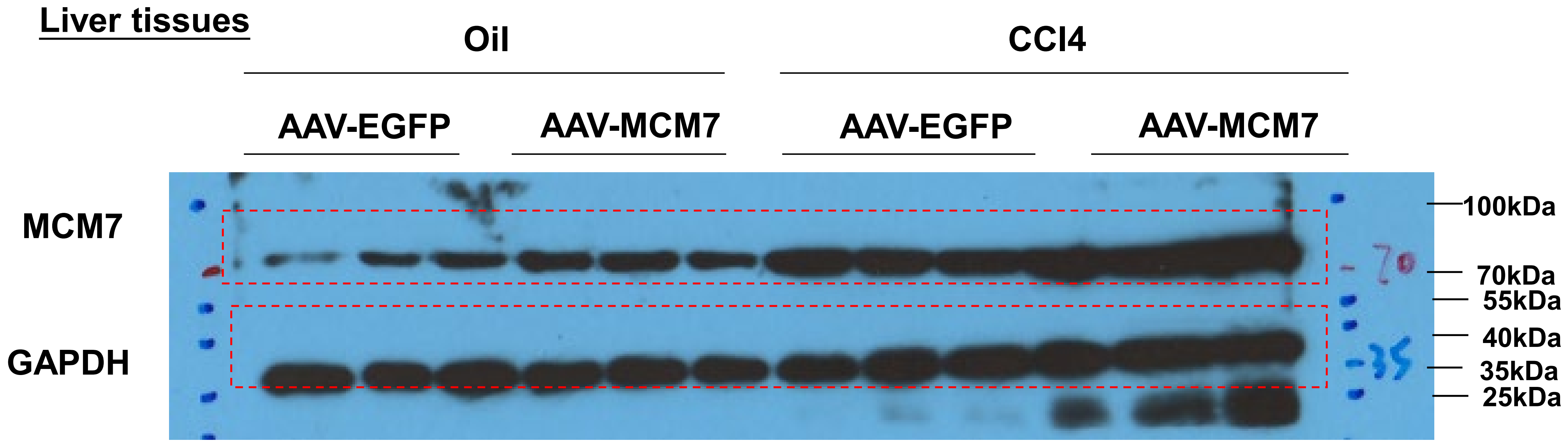

The original protein Western blot images for Figure S4 and S5， The red dashed boxes indicate the protein bands that are referenced in the main text.

Fig. S4F

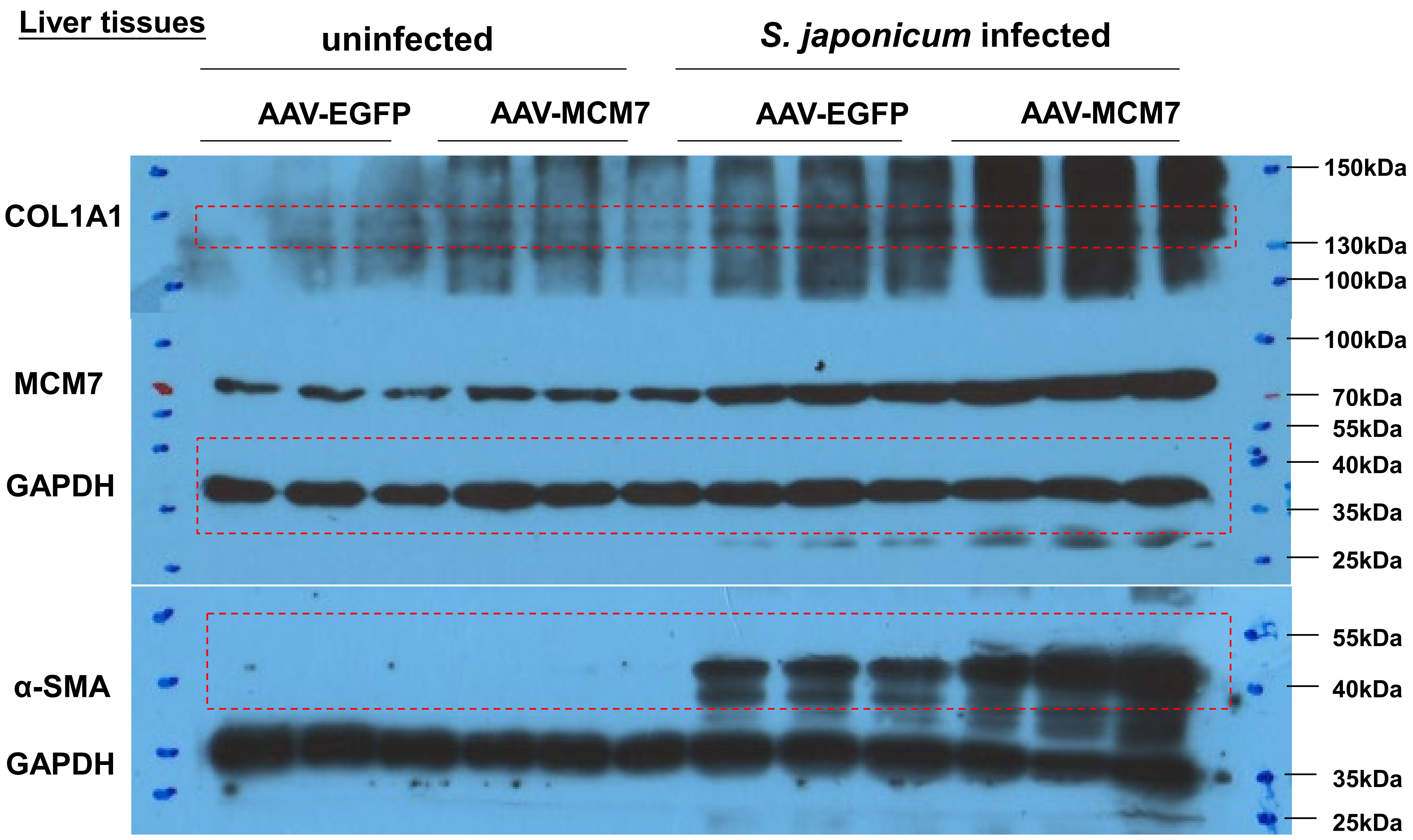

Fig. S5E

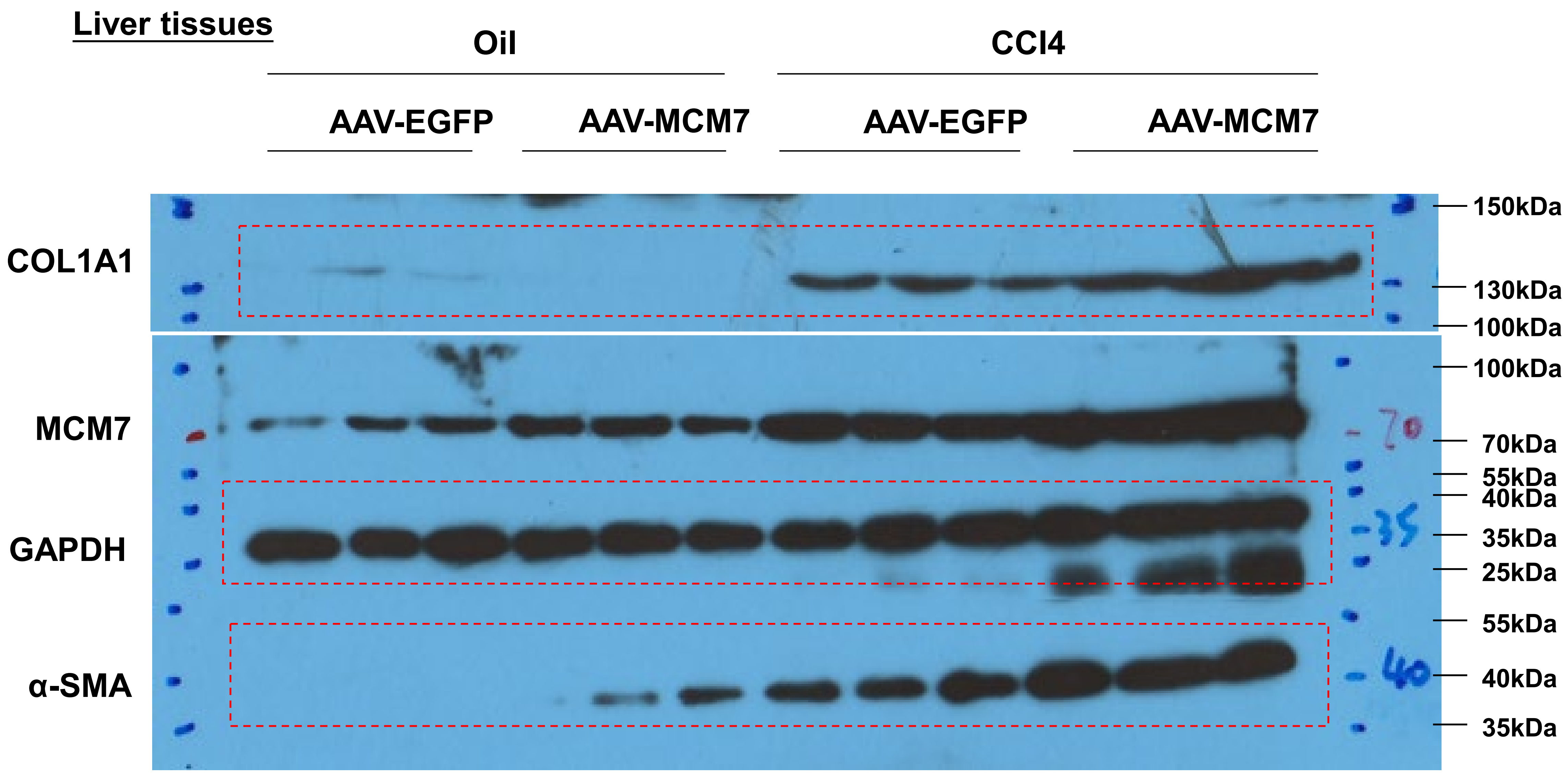

The original protein Western blot images for Figure S6, The red dashed boxes indicate the protein bands that are referenced in the main text.

Fig. S6A

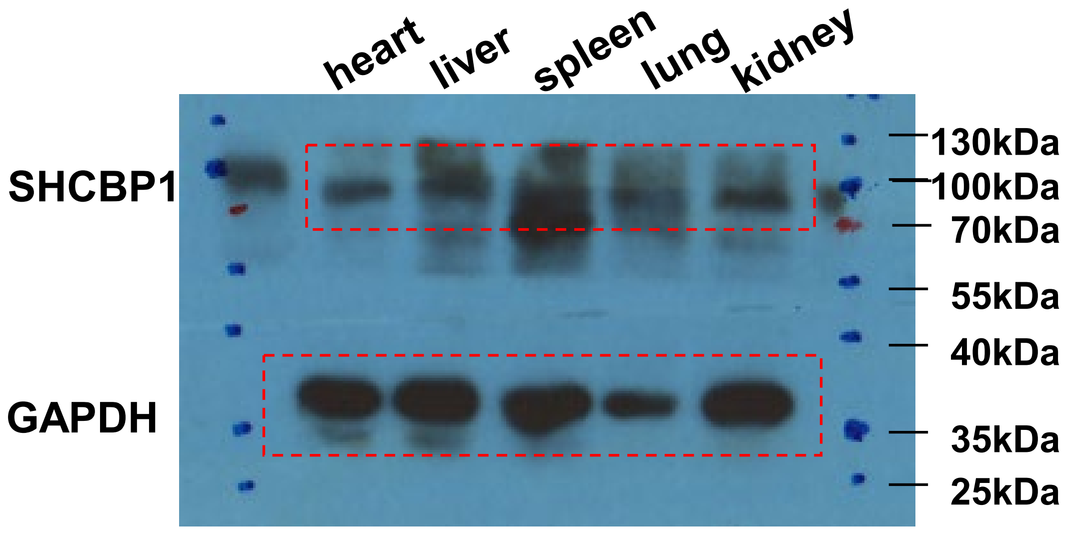

Fig. S6B

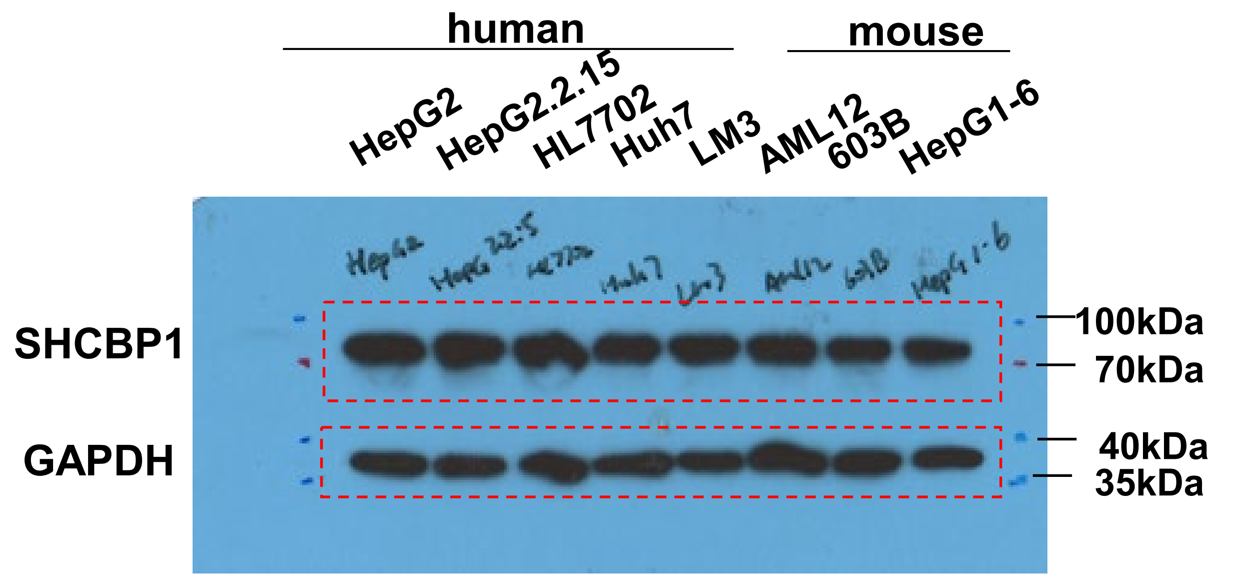

Fig. S6C

HepG2

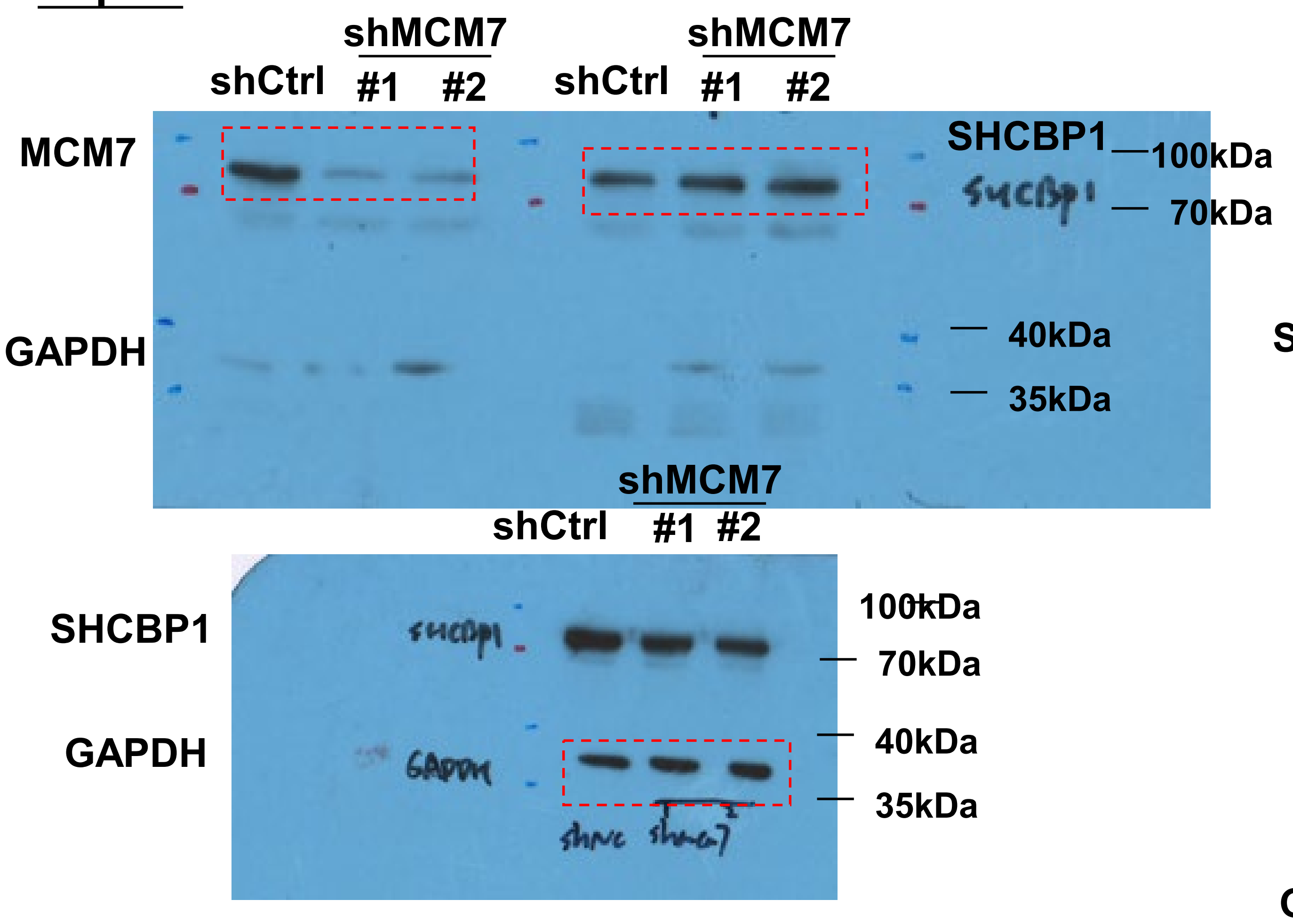

HepG2

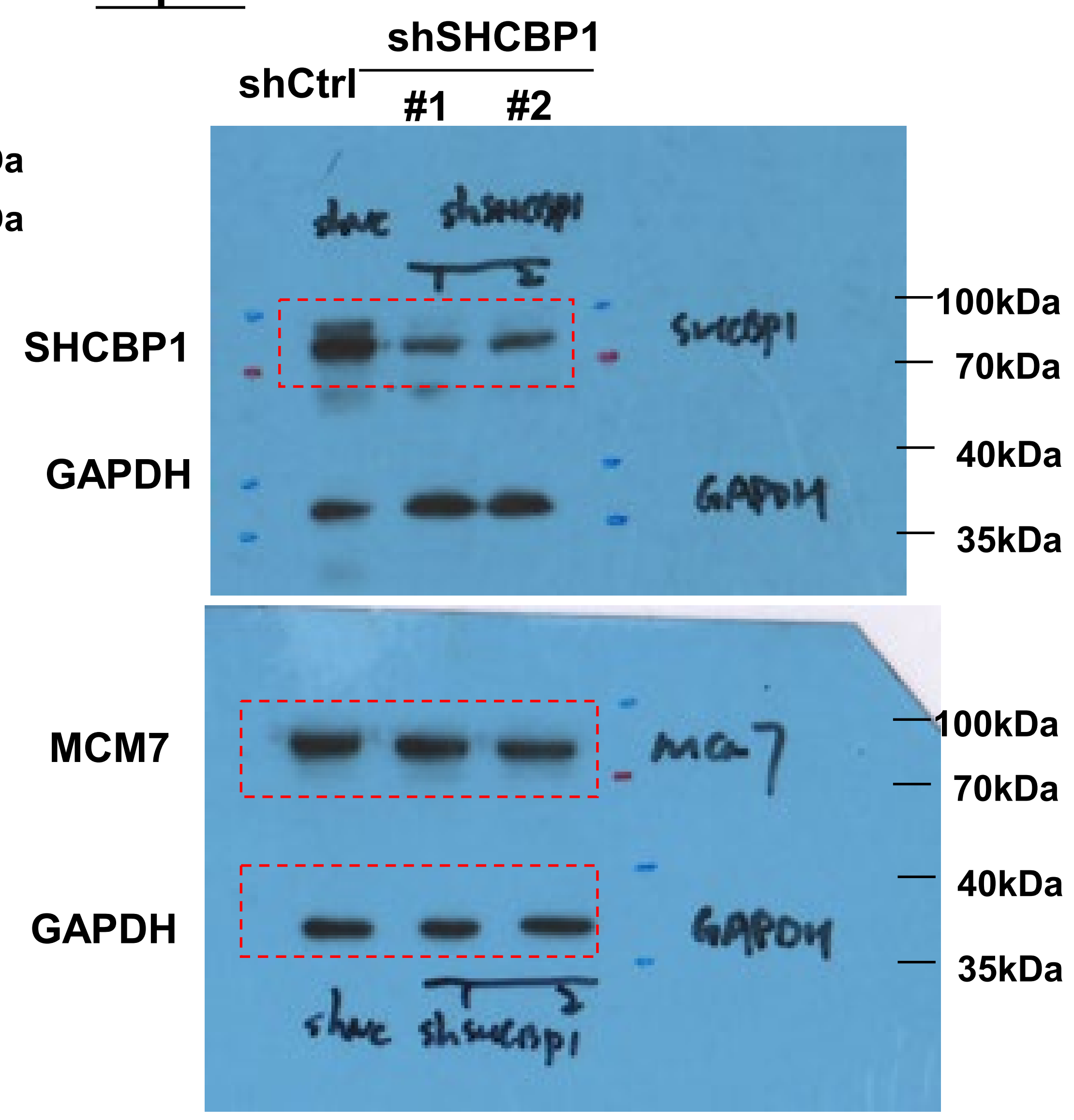

Fig. S6D

293T

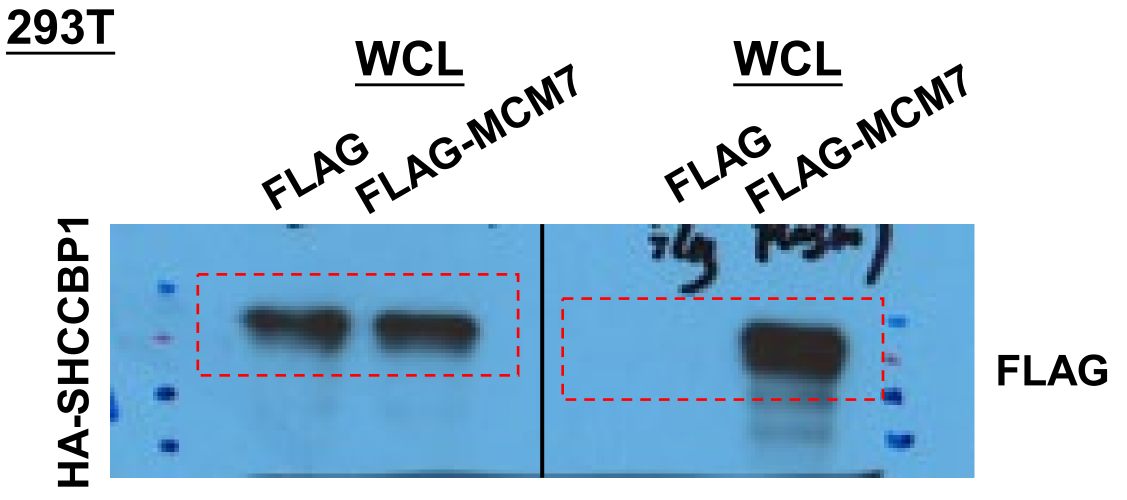

293T

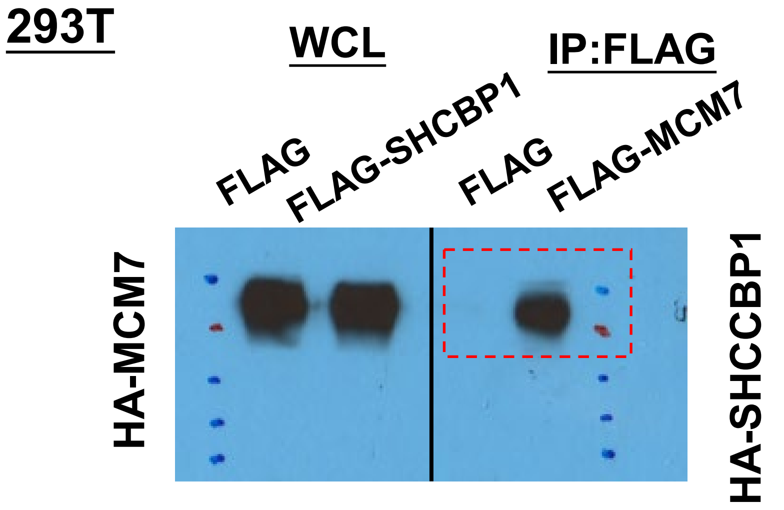

293T

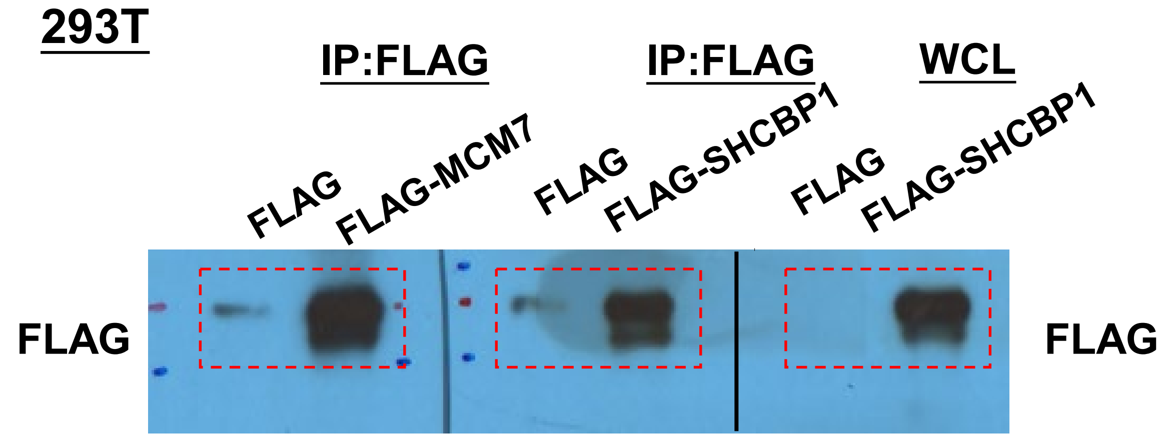

293T

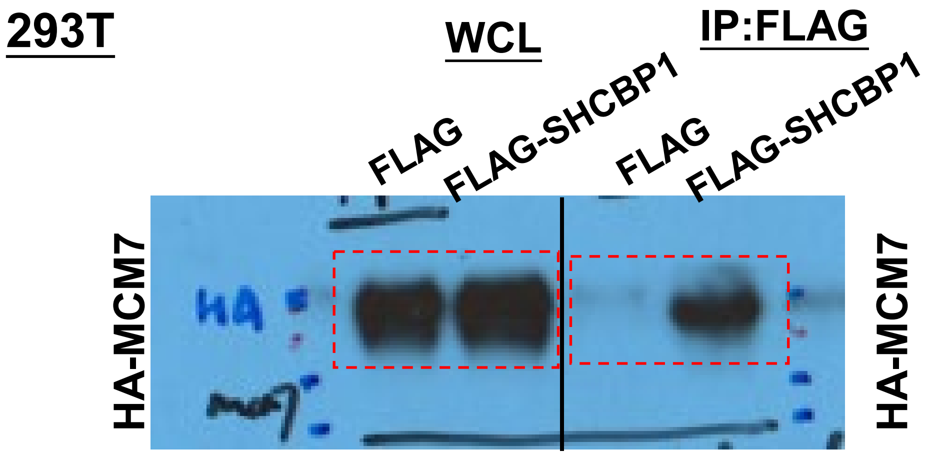

The original protein Western blot images for Figure S6, The red dashed boxes indicate the protein bands that are referenced in the main text.

Fig. S6E

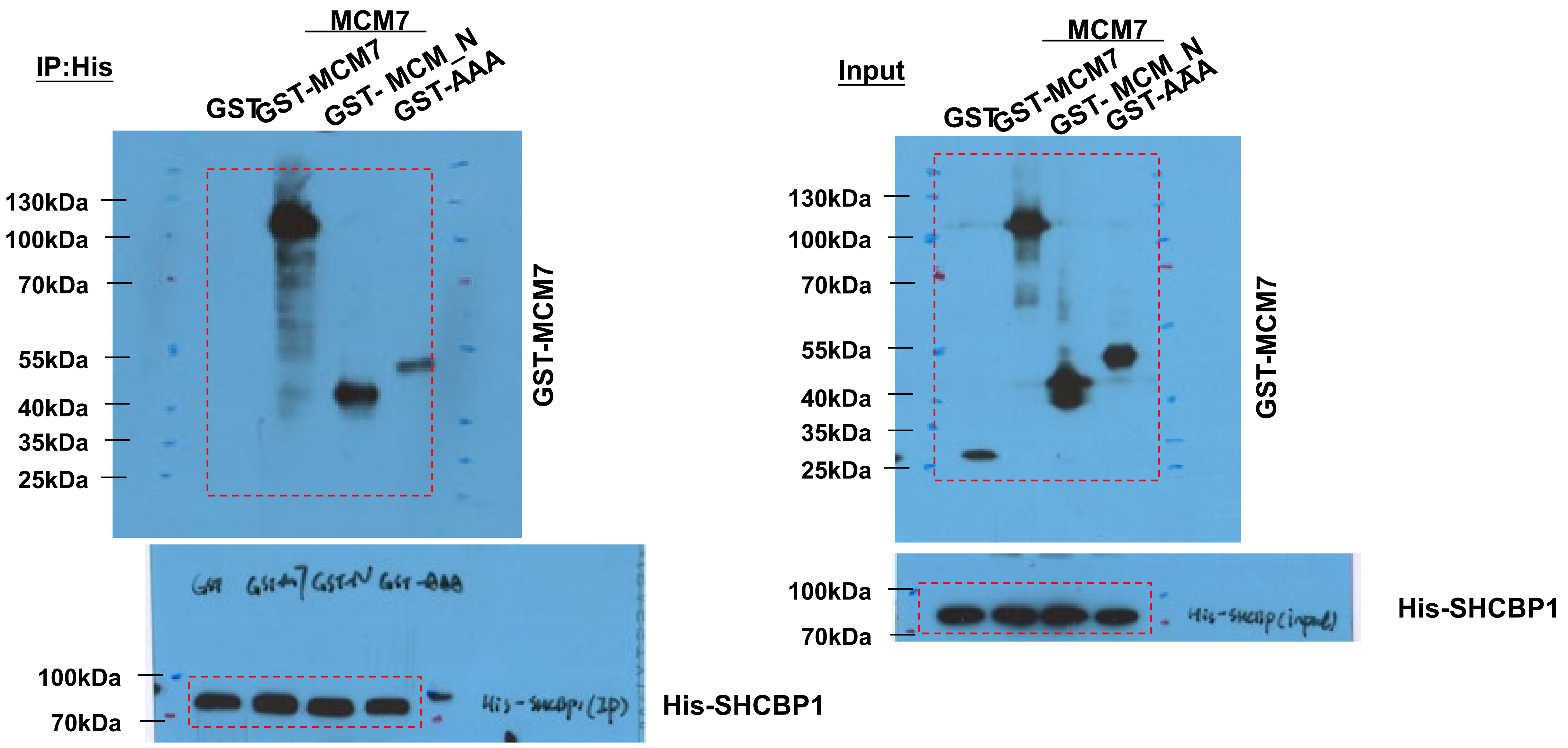

Fig. S6F

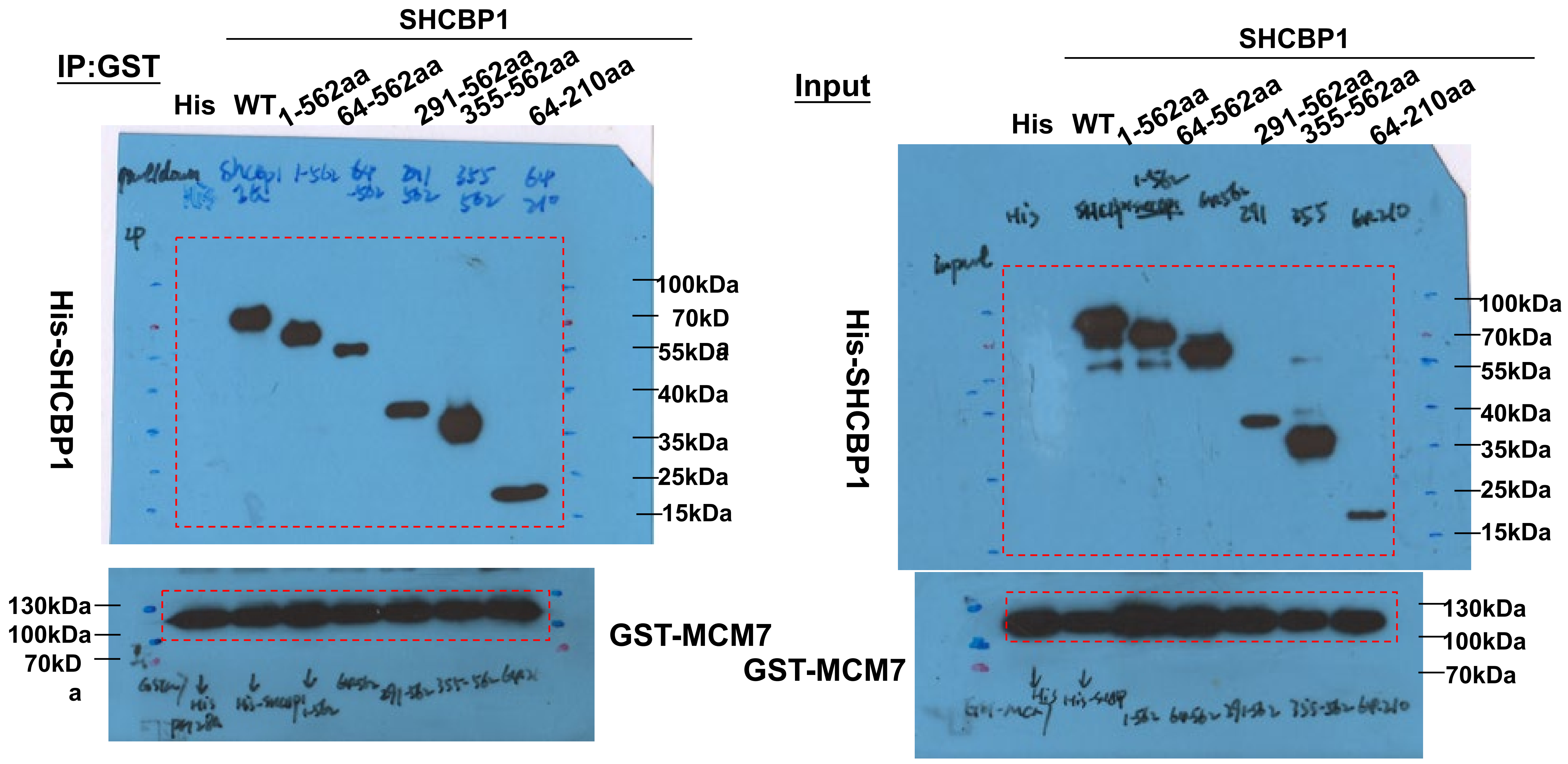

The original protein Western blot images for Figure S7, The red dashed boxes indicate the protein bands that are referenced in the main text.

Fig. S7I

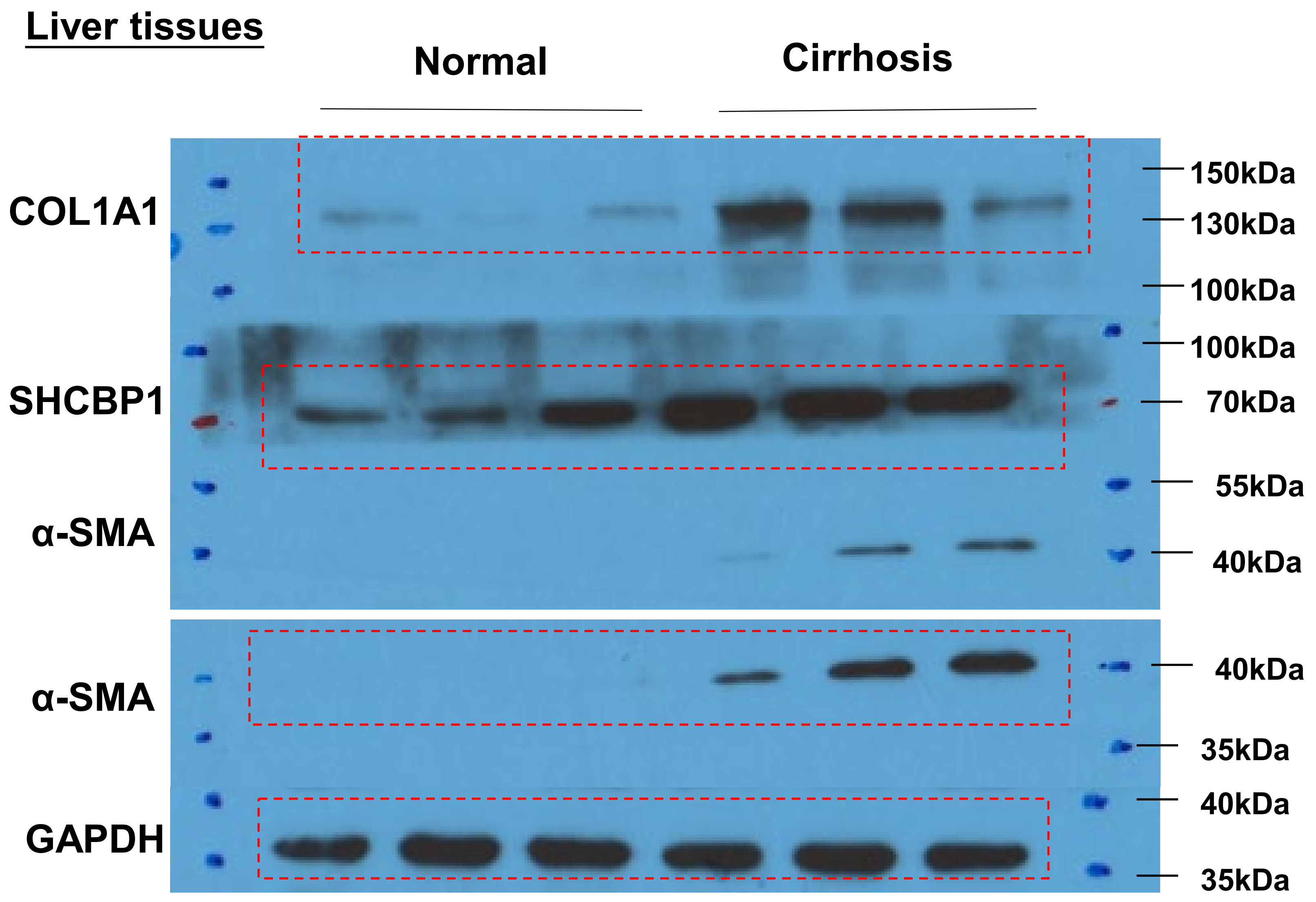

Fig. S7L

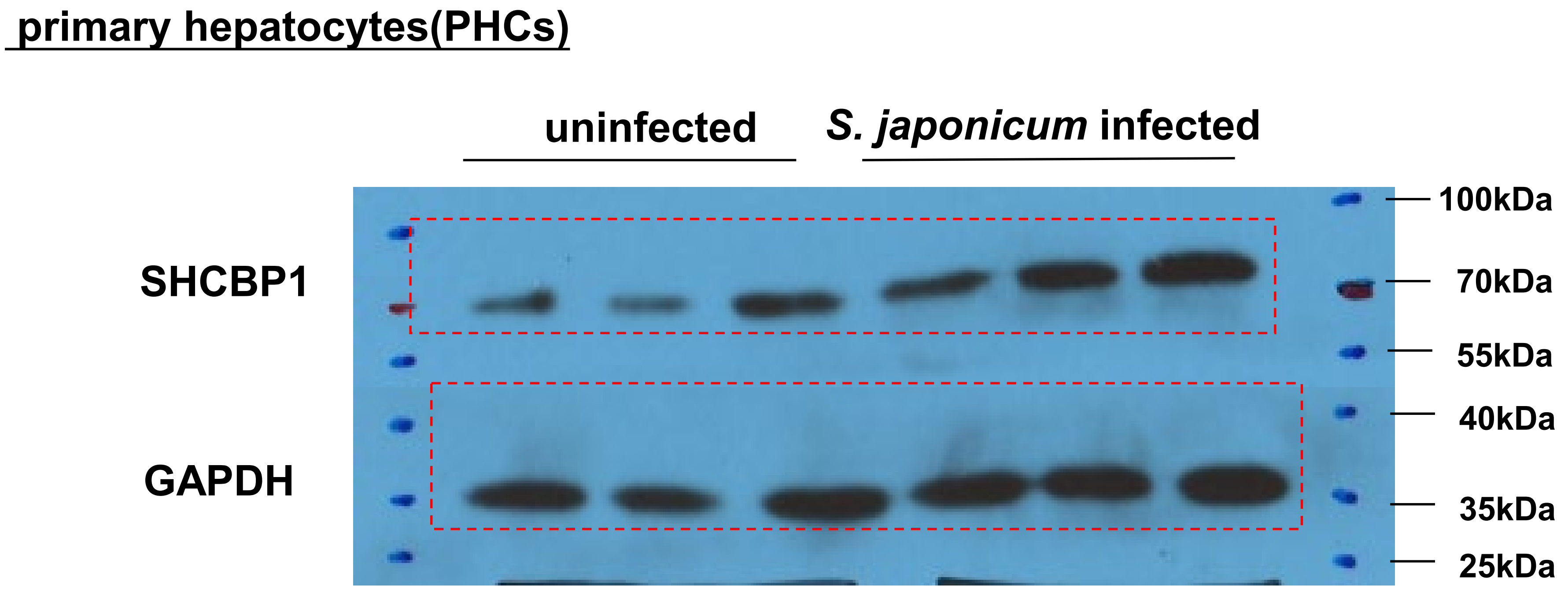

Fig. S7N

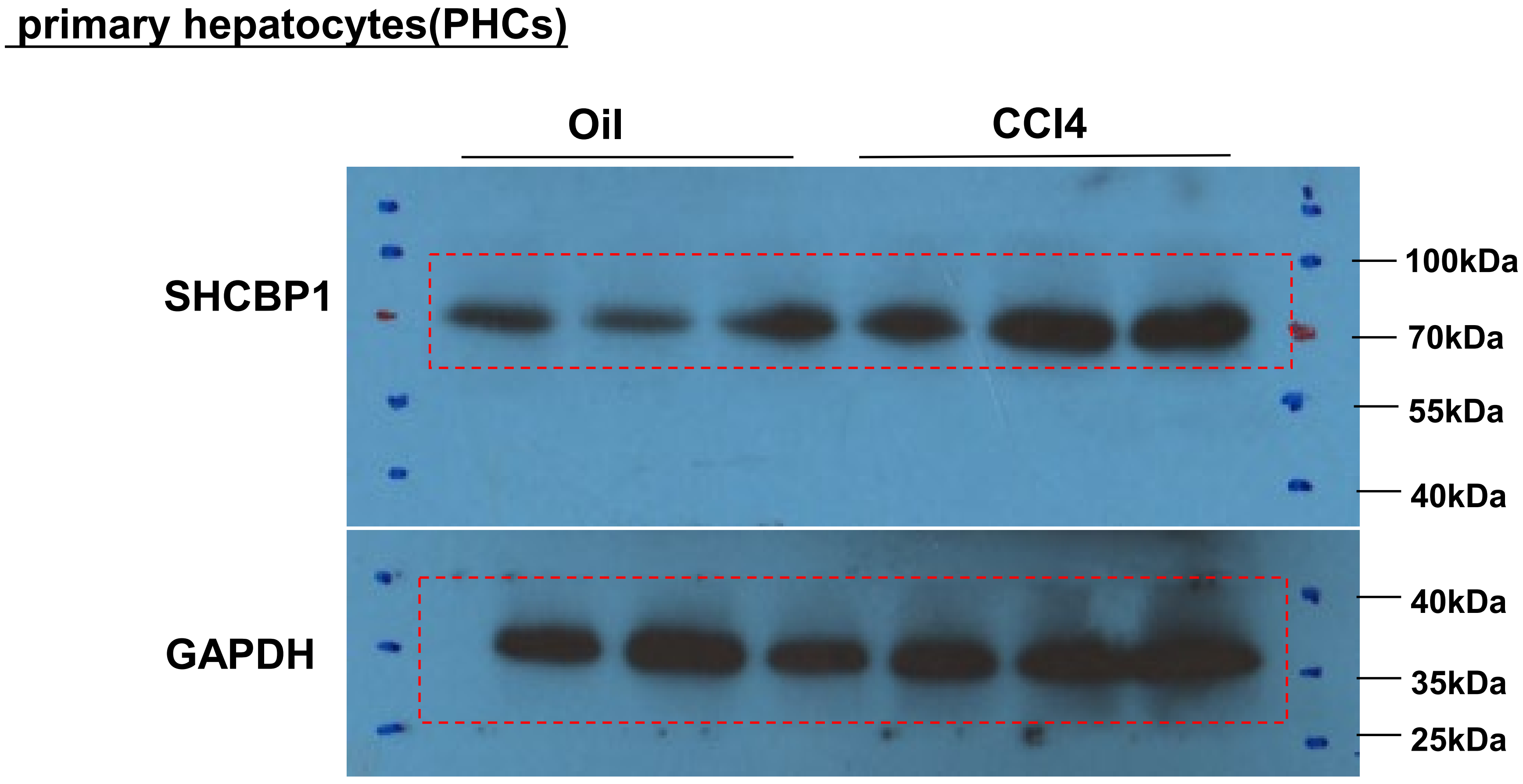

The original protein Western blot images for Figure S8, The red dashed boxes indicate the protein bands that are referenced in the main text.

Fig. S8A

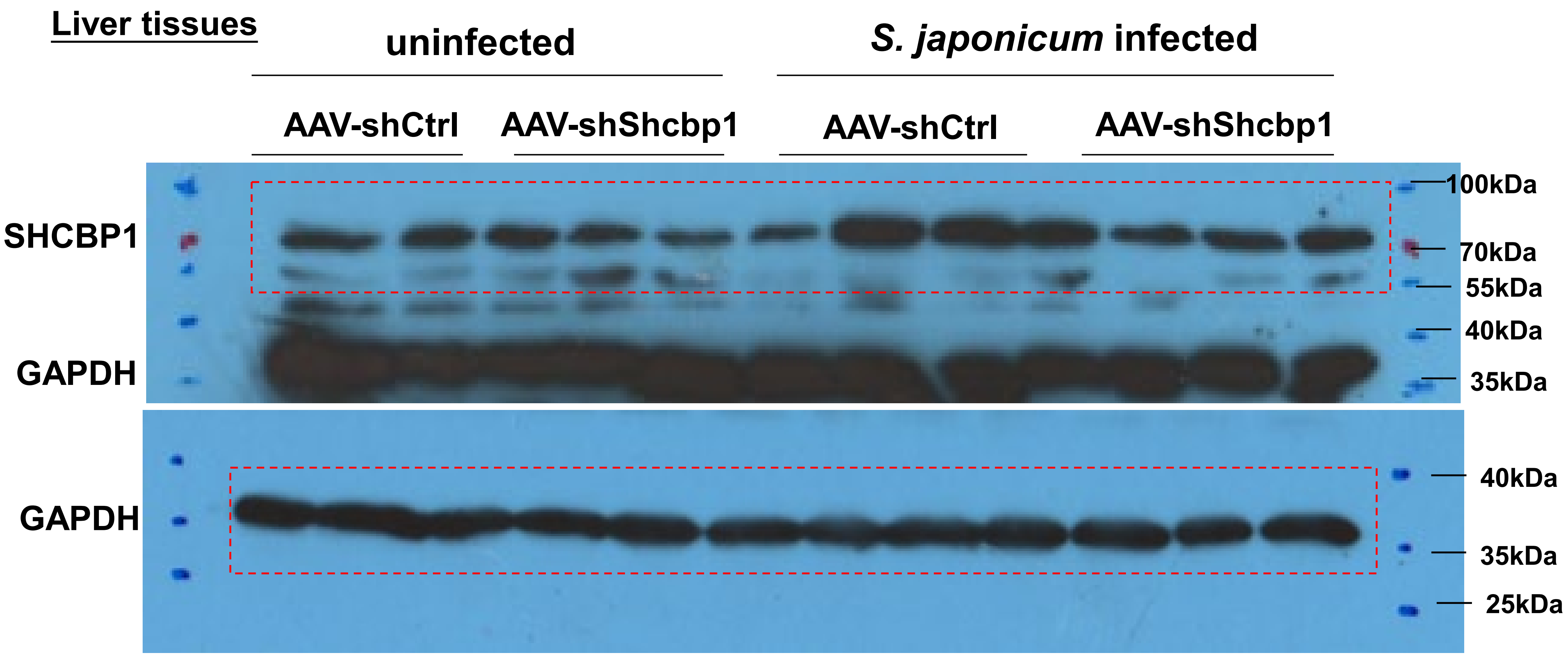

Fig. S8B

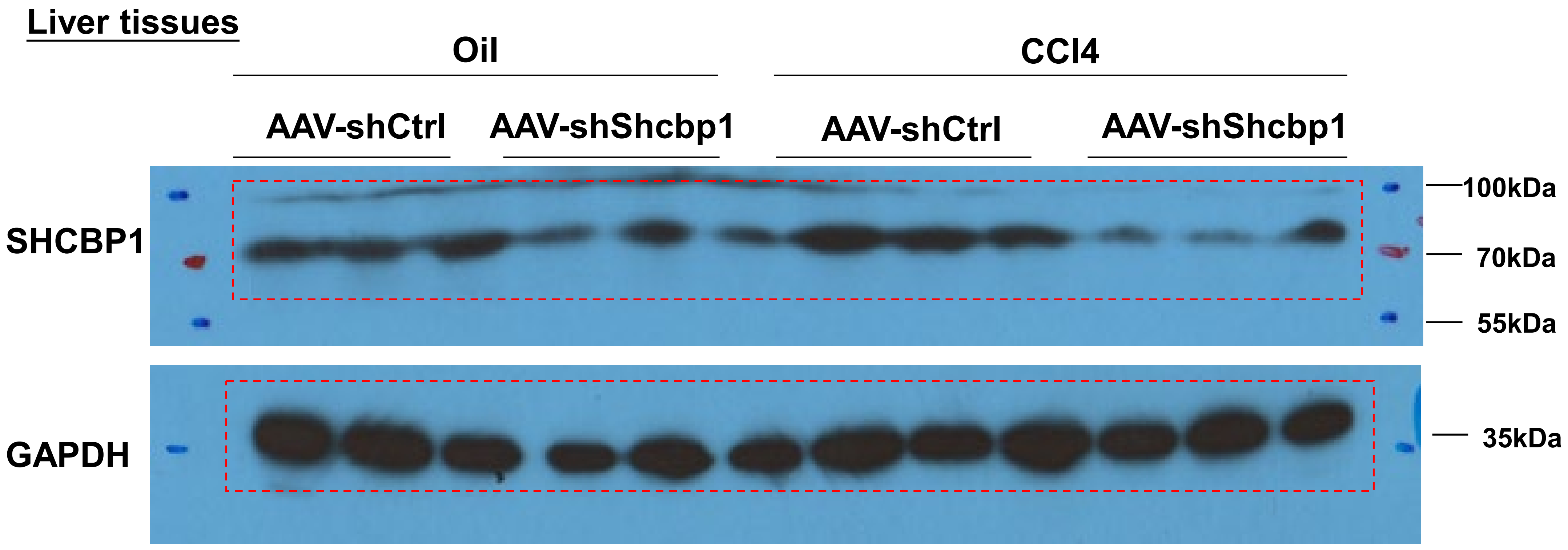

Fig. S8C

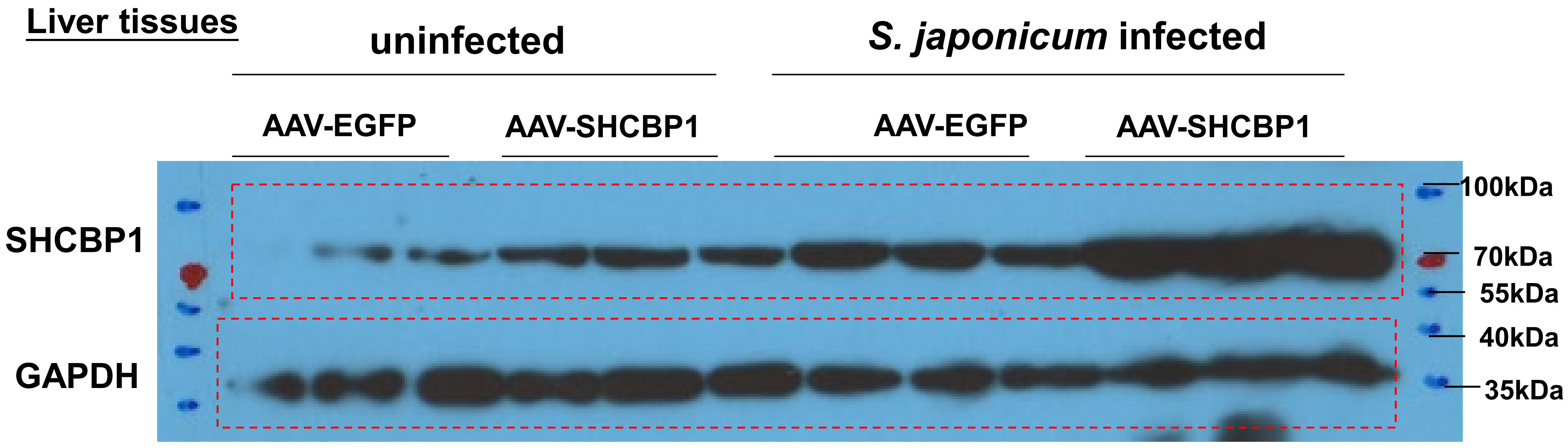

Fig. S8D

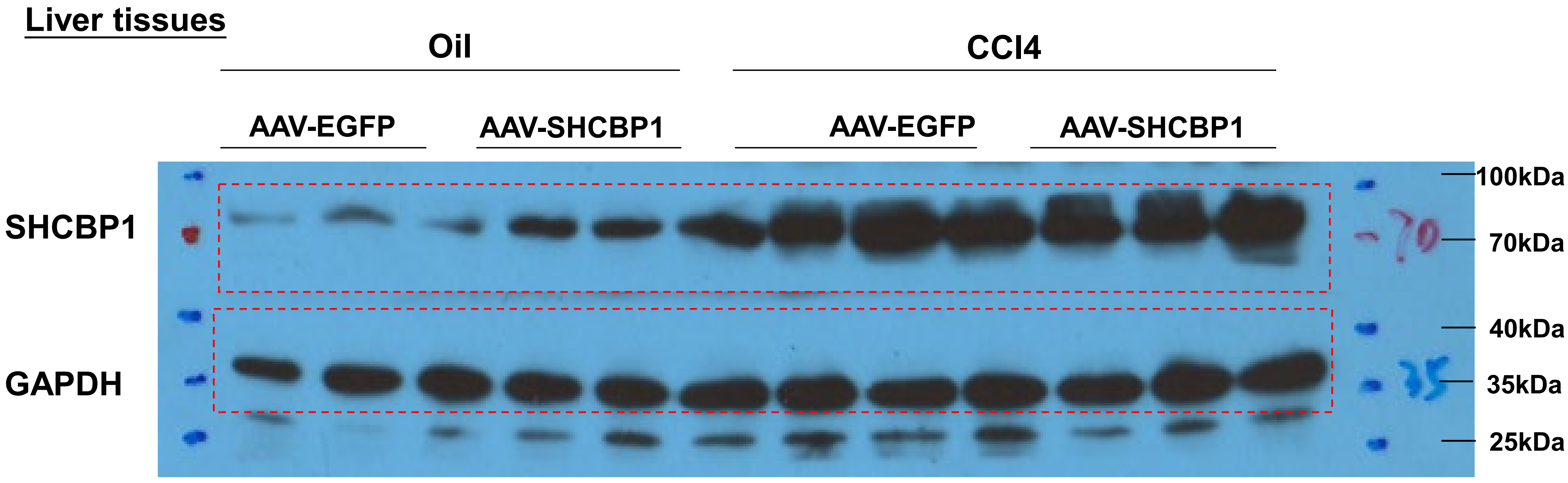

The original protein Western blot images for Figure S9，S10, and S11，The red dashed boxes indicate the protein bands that are referenced in the main text.

Fig. S9E

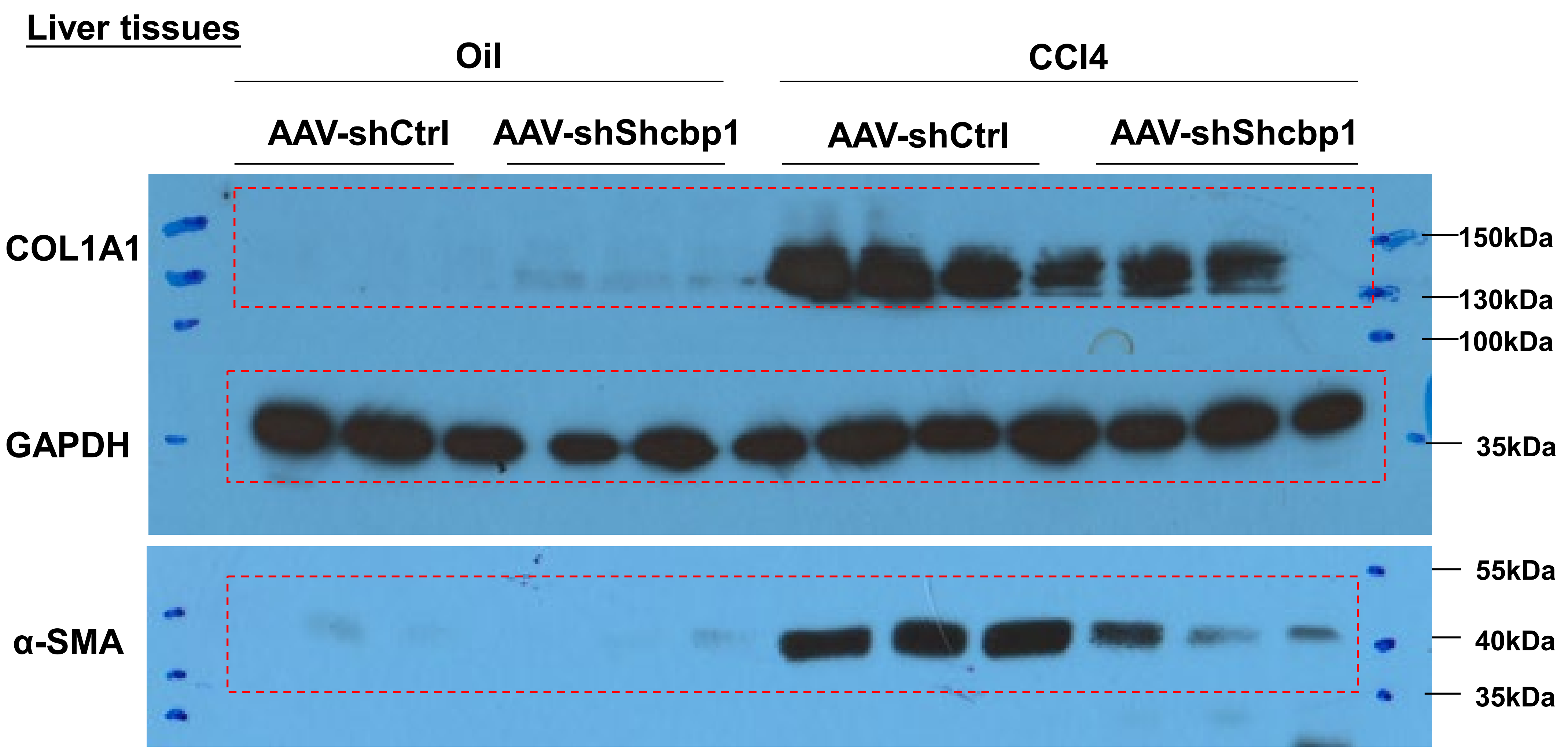

Fig. S10F

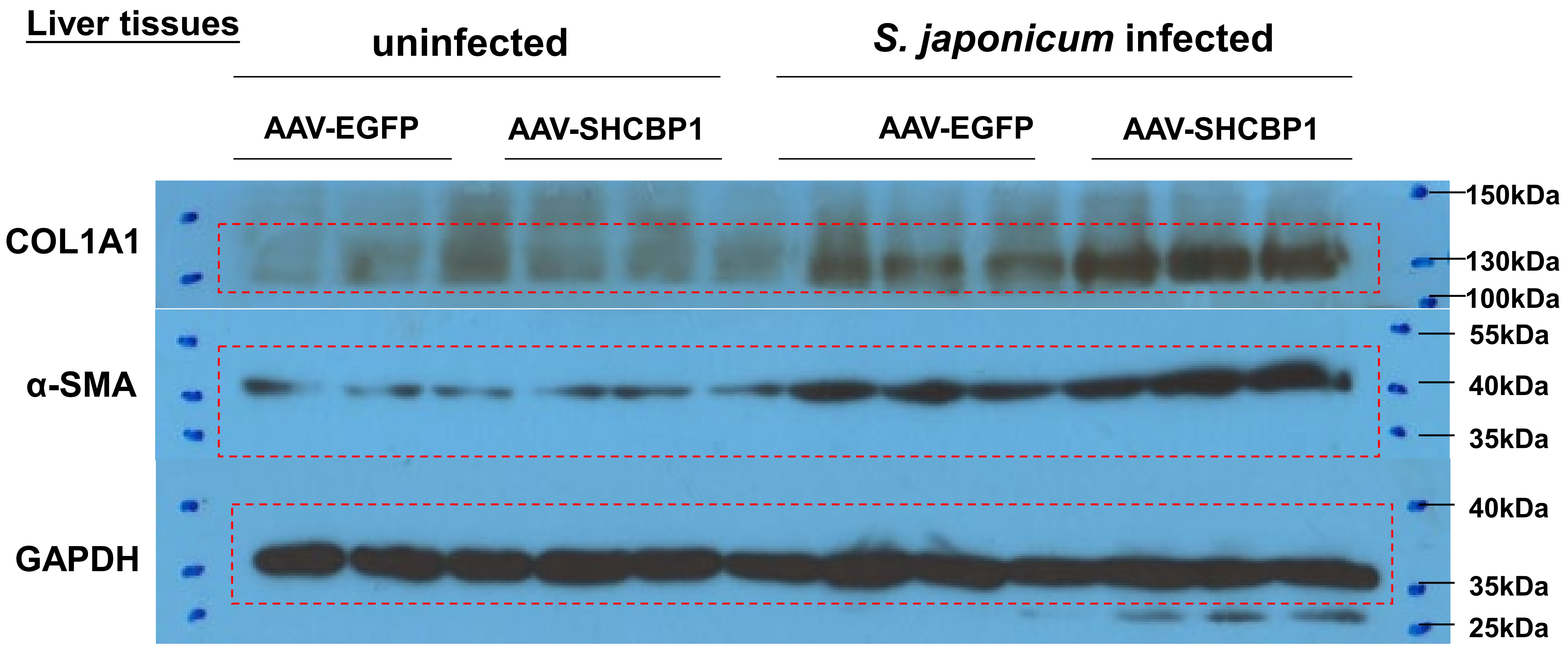

Fig. S11E

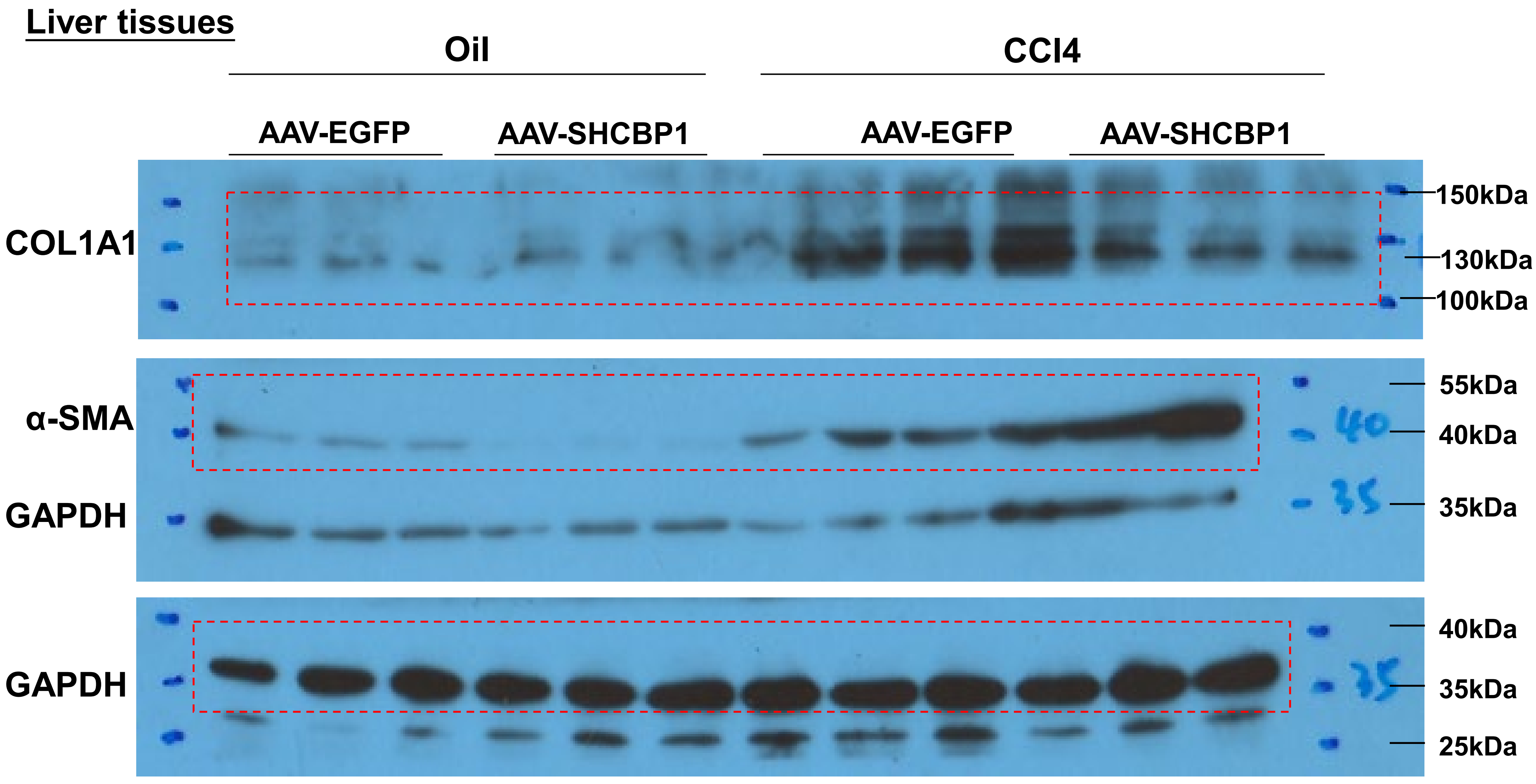

The original protein Western blot images for Figure S12，The red dashed boxes indicate the protein bands that are referenced in the main text.

Fig. S12A

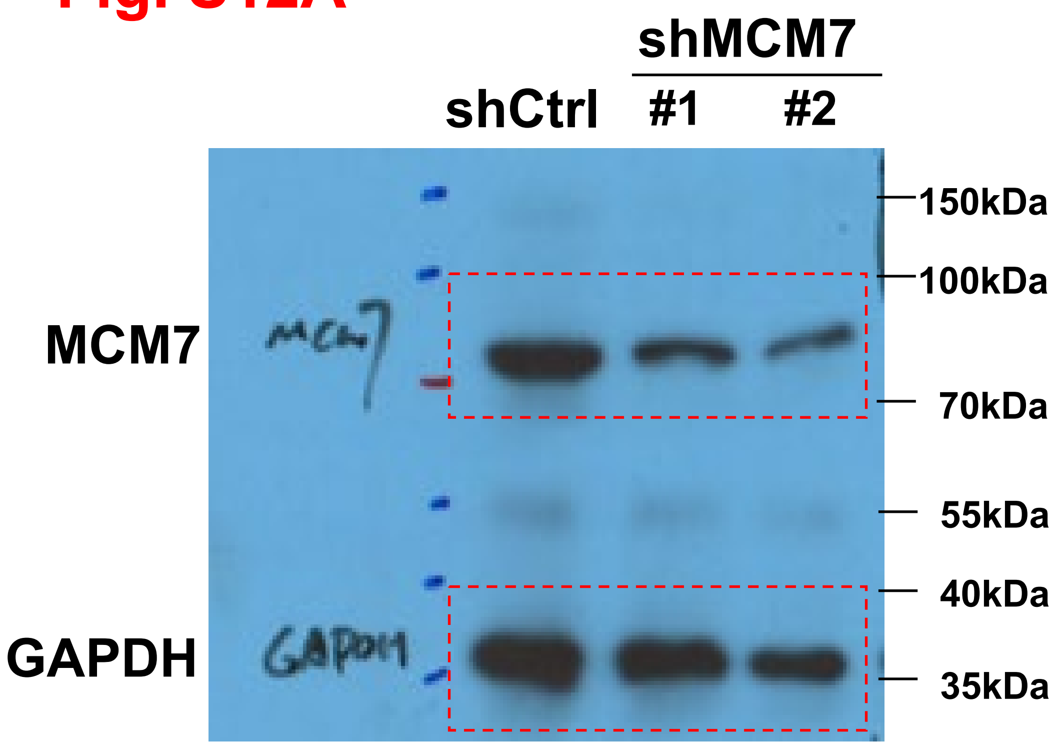

Fig. S12B

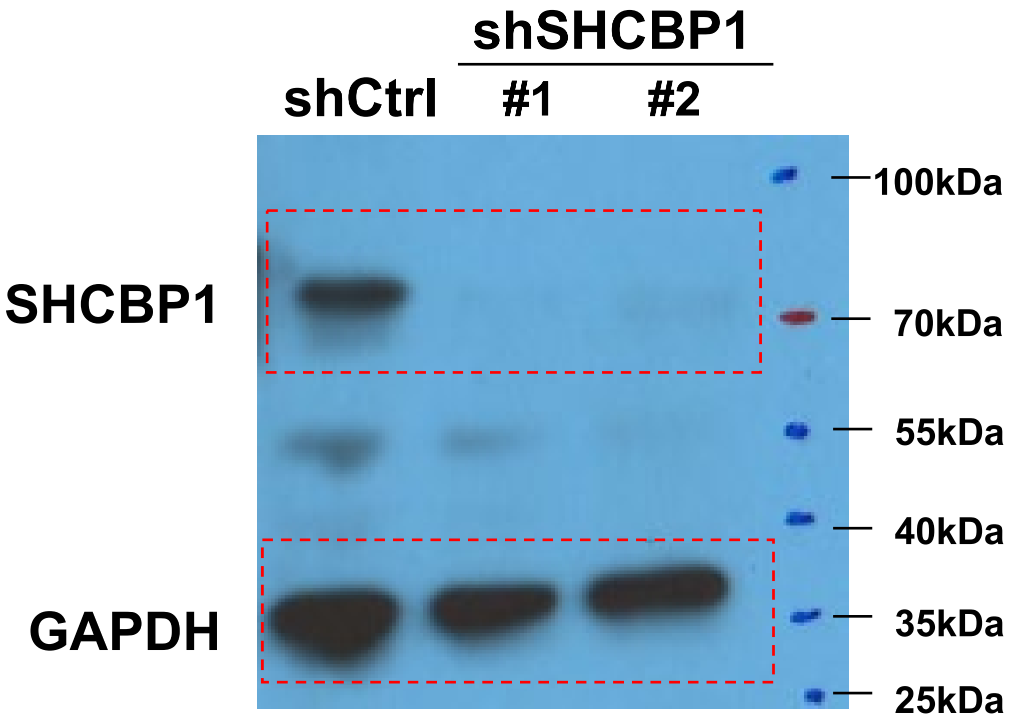

The original protein Western blot images for Figure S16, The red dashed boxes indicate the protein bands that are referenced in the main text.

Fig. S16A

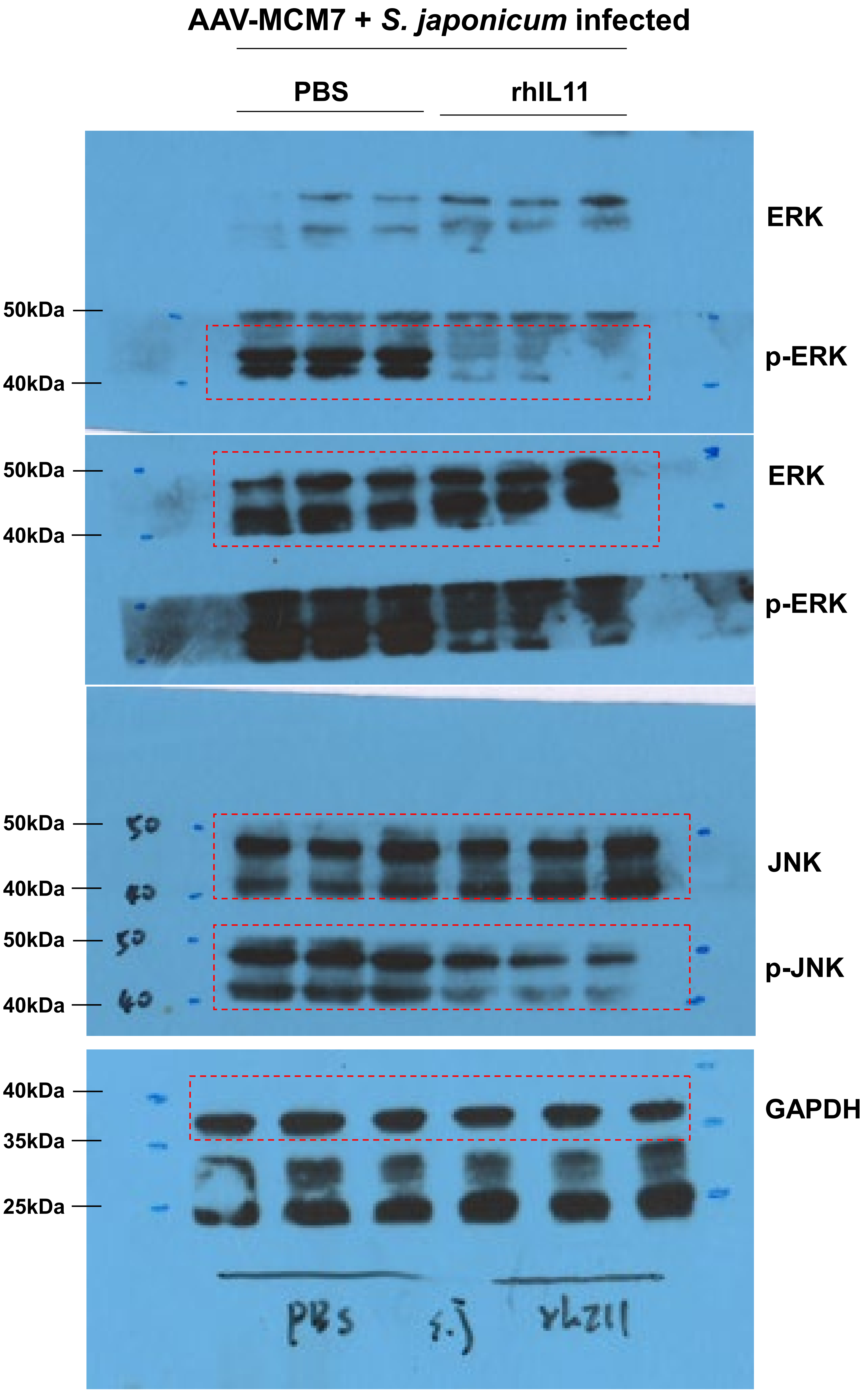

The original protein Western blot images for Figure S16, The red dashed boxes indicate the protein bands that are referenced in the main text.

Fig. S16B

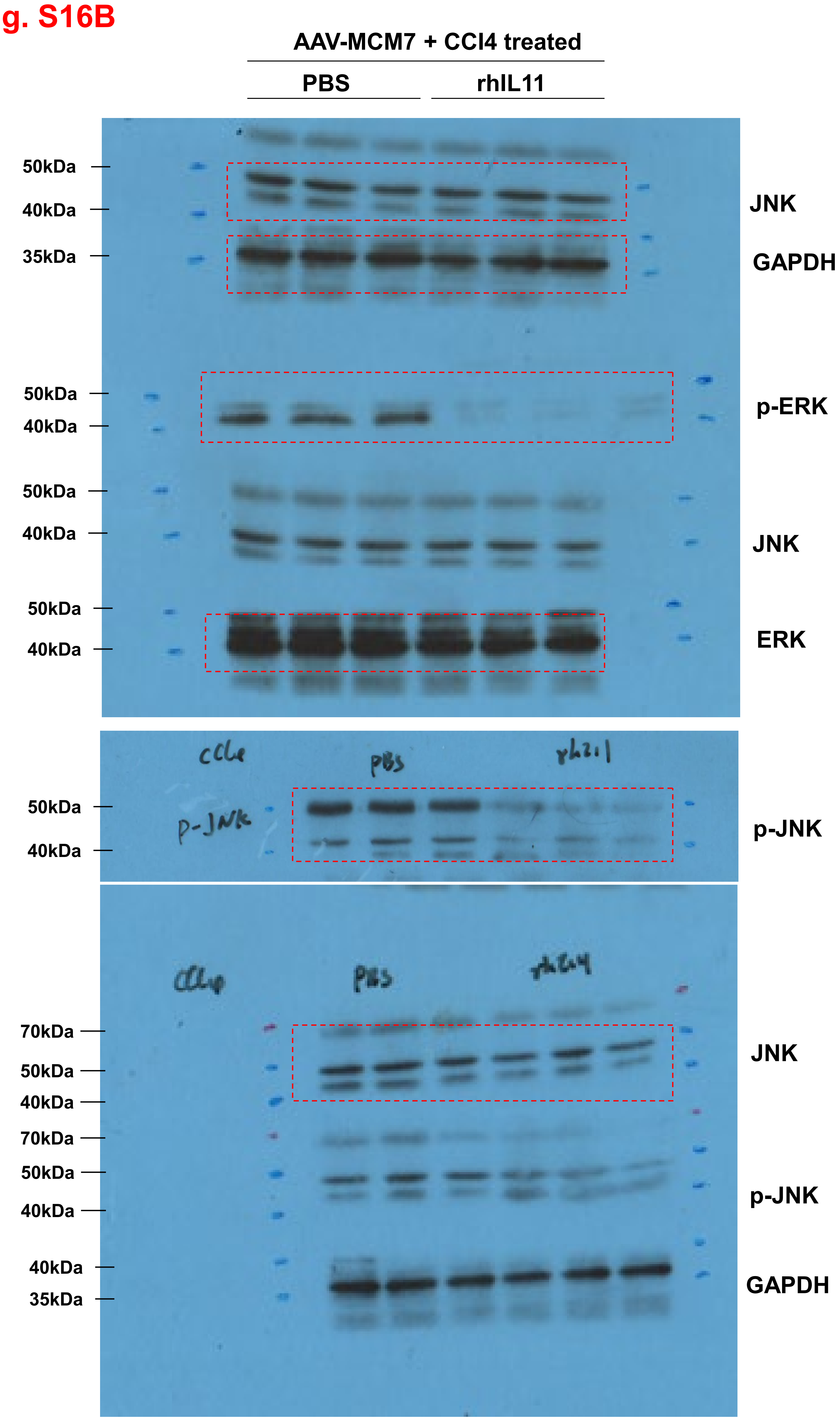

The original protein Western blot images for Figure S17, The red dashed boxes indicate the protein bands that are referenced in the main text.

Fig. S17E

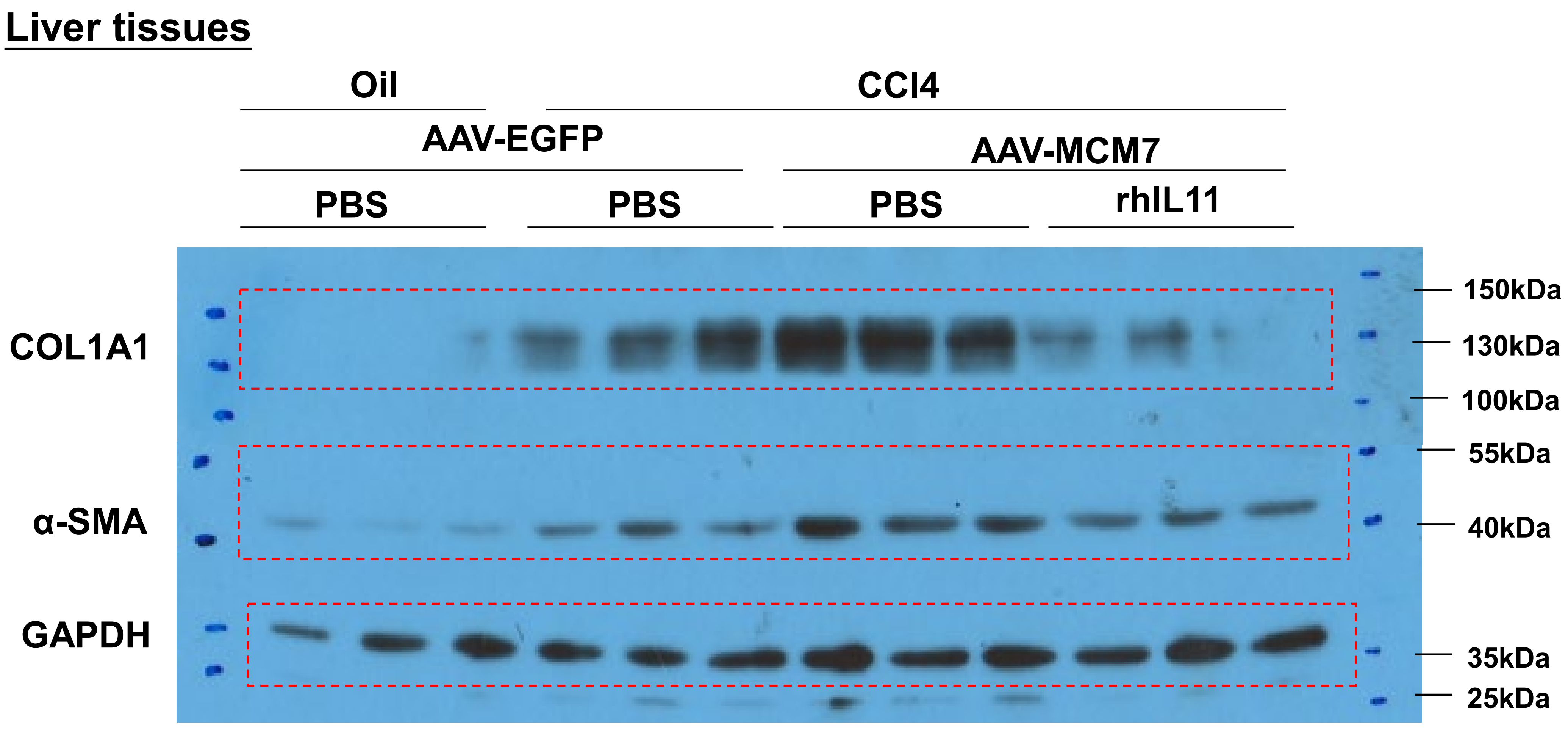

Supplement: Supplementary file 2 — original western blots [file 41419_2025_7937_MOESM2_ESM.pdf]
